# Mechanistic Study of NT5E in Reg3β-Induced Macrophage Polarization and Cooperation with Plasma Proteins in Myocarditis Injury and Repair

**DOI:** 10.3390/biology14081017

**Published:** 2025-08-07

**Authors:** Shichao Zhang, Peirou Zhou, Fanfan Zhu, Yingying Wang, Xuesong Wang, Jingwen Chen, Yumeng Li, Xiaoyi Shao

**Affiliations:** Department of Immunology, Medical School, Nantong University, Nantong 226019, China

**Keywords:** myocarditis, NT5E, Reg3β, plasma proteins, macrophage polarization

## Abstract

**Simple Summary:**

Myocarditis is a cardiac disease characterized by the destruction of myocardial cells and localized or diffuse inflammatory lesions in the myocardium. It is especially common in children and adolescents. Previous studies have reported that Reg3β, produced by cardiomyocytes, recruits macrophages to the heart and plays a crucial role in myocardial tissue repair following myocardial infarction. In this study, we found that NT5E is involved in Reg3β-induced macrophage polarization. Using Mendelian randomization (MR) analysis, we further identified that NT5E interacts with multiple plasma proteins influencing myocarditis progression through immune cells. Additionally, we explored the interactions among these proteins and screened several potential drugs for myocarditis treatment. Finally, phenome-wide association studies (PheWASs) were employed to evaluate the safety of ten proteins as therapeutic targets.

**Abstract:**

**Background:** We aimed to explore the mechanism by which extracellular-5′-nucleotidase (NT5E) regulates macrophage polarization via regenerating islet-derived protein 3 beta (Reg3β) and other plasma proteins that mediate immune-cell effects on myocarditis. Methods: The involvement of NT5E in Reg3β-induced macrophage polarization was first analyzed using RNA sequencing, Western blotting, and quantitative polymerase chain reaction. Mendelian randomization was employed to identify NT5E and various plasma proteins as potential therapeutic targets for myocarditis. Mediation analysis, enrichment analysis, protein–protein interaction network analysis, drug prediction, molecular docking, and single-cell RNA sequencing were integrated to further evaluate the biological functions and pharmacological potential of the identified targets. Finally, phenome-wide association studies were conducted to assess the safety of targeting these proteins. **Results:** NT5E expression was elevated in Reg3β-stimulated M2 macrophages. The expression of Arg-1, a marker of M2 macrophages, decreased upon NT5E knockdown, suggesting that NT5E is involved in the Reg3β-mediated polarization of macrophages to the M2 phenotype. Mendelian randomization analysis identified NT5E and 80 other plasma proteins as being causally associated with myocarditis. Mediation analysis revealed 12 immune-cell types were mediators of the effects of plasma protein on myocarditis progression. Drug prediction identified candidates such as ICN 1229 and chrysin, which showed strong binding affinities in molecular docking analyses. These findings may contribute to the development of effective treatments for myocarditis. **Conclusions**: NT5E plays a dual role in Reg3β-induced macrophage polarization and in interacting with plasma proteins that influence the onset and progression of myocarditis through immune-cell pathways.

## 1. Introduction

Myocarditis is a cardiac disease characterized by myocardial cell destruction and limited or diffuse inflammatory lesions in the myocardium [1]. It is common in children and adolescents [2,3]. The disease may be asymptomatic in mild cases, but in severe cases, it can lead to serious complications such as arrhythmias, cardiac insufficiency, cardiogenic shock, heart failure, or even sudden cardiac death [4,5,6]. The most common causes of myocarditis are viral infections, with Coxsackie group B viruses being predominant, followed by bacterial and fungal infections [7]. Although some progress has been made in the diagnosis and treatment of myocarditis in recent years, its specific mechanisms of onset and progression still require further research. Exploring the inflammatory damage caused by myocarditis, understanding the mechanisms of myocardial function remodeling, and identifying specific therapeutic targets are crucial for preventing the onset of myocarditis and improving patient outcomes.

In myocarditis, inflammation leads to necrosis of cardiomyocytes, the release of damage-associated molecular patterns such as high-mobility group protein B1, and the activation of cardiac-resident immune cells, including mast cells. This results in mass infiltration of monocytes and macrophages into myocardial tissue, contributing to the development and progression of the disease [8,9]. Myocardial infiltrating macrophages participate in the stages of myocardial injury, cardiomyocyte regeneration, and functional remodeling by undergoing reprogramming within the local microenvironment [10,11]. Regenerating islet-derived protein 3 beta (Reg3β) was initially identified as a C-type lectin-like protein expressed during the regeneration of pancreatic islets after pancreatitis and injury. Its homologs Reg3α and Reg3y have also been identified in mice [12,13]. Produced by cardiomyocytes, Reg3β recruits macrophages to the heart, playing an important role in myocardial tissue damage and repair after myocardial infarction [14,15]. In autoimmune myocarditis induced by MyHC-α, Reg3β expression is also upregulated in mouse hearts [16], where it promotes macrophage phagocytosis and proliferation, induces M2, and facilitates the repair of injured myocardium. However, the specific mechanism by which Reg3β regulates macrophage polarization involved in myocardial tissue repair remains to be further investigated.

The human plasma proteome consists of proteins secreted or shed into the circulation, where they perform functions such as mediating intercellular communication [17]. Plasma proteins are important biomarkers of cardiovascular disease and are used clinically for diagnosis and risk stratification [18]. In addition to being disease markers, plasma proteins contribute to systemic homeostasis through roles in immune response, vascular and endothelial function, tissue remodeling, fluid exchange, and nutrient absorption [19]. For instance, while protein biomarkers such as troponin I and CK-MB significantly change in blood levels after myocardial injury and aid in early diagnosis, they may not be the direct cause of disease. Genome-wide association studies (GWASs) have identified genetic variants associated with plasma protein levels, referred to as protein quantitative trait loci (pQTLs) [20,21,22]. These pQTLs allow for the causal assessment of potential drug targets for human diseases using Mendelian randomization (MR) [23]. Therefore, exploring the role of the plasma proteome in myocarditis is warranted.

The aim of this study was to experimentally verify, through RNA sequencing (RNA-seq) and other analyses, that NT5E is an important factor in Reg3β-mediated macrophage polarization during myocarditis injury repair. We also systematically identified NT5E, multiple plasma proteins, and potential drug targets causally associated with myocarditis through an integrated MR framework. The pharmacological activity of these targets was validated through a combination of drug prediction and molecular docking analyses, thereby expanding the clinical applicability of the drug candidates. In addition, mediation analysis was used to identify immune cells that mediate the effects of plasma protein on myocarditis. Single-cell sequencing was utilized to analyze changes in cell subpopulations and the expression of myocarditis-associated proteins. Finally, phenome-wide association studies (PheWASs) were conducted to investigate the safety of targeting these proteins.

## 2. Materials and Methods

### 2.1. Data Sources

The Gene Expression Omnibus (GEO) is a publicly accessible database http://www.ncbi.nlm.nih.gov/geo/, 2025 (accessed on 20 January 2025) that houses a vast collection of high-throughput sequencing and microarray datasets relevant to various diseases [24]. In this study, we retrieved the gene expression dataset GSE142564, which is associated with myocarditis, from the GEO database. The GWAS dataset used in this study was sourced from the publicly available IEU GWAS Database (GWAS ID: ebi-a-GCST90018882), https://api.opengwas.io/, 2025 (accessed on 20 January 2025). The GWAS data on myocarditis included 427,911 individuals of European descent, comprising 633 cases and 427,278 controls. We examined 4907 pQTLs [25] in a dataset of 35,553 Icelanders, and single-nucleotide polymorphisms (SNPs) linked to plasma protein levels at genome-wide significance (*p* < 5 × 10^−8^) were selected as instrumental variables (IVs) from the deCODE GWAS study, https://www.decode.com/summarydata/, 2025 (accessed on 20 January 2025) (Table S1). Additionally, data encompassing 731 immune-cell characteristics were procured from the GWAS catalog (https://api.opengwas.io/ [accessed on 20 January 2025]), ranging from Ebi-a-GCST0001391 to Ebi-a-GCST0002121 (Table S2). Immune phenotypes were classified using flow cytometry into absolute cell counts (ACs), relative cell counts (RCs), median fluorescence intensities (MFIs), and morphological parameters (MPs), and were grouped into seven panels: B cells (*n*  =  190), Tregs (*n * =  167), TBNKs (*n * =  124), T-cell maturation stages (*n*  =  79), dendritic cells (*n * =  64), myeloid cells (*n*  =  64), and monocytes (*n*  =  43) [25].

### 2.2. Cell Culture and RNA-Seq Technical Analysis

Macrophages were derived from the RAW264.7 macrophage line (purchased from the National Experimental Cell Resource Sharing Platform). RAW264.7 monocytes were cultured in DMEM (Gibco, Grand Island, NY, USA) supplemented with 10% fetal bovine serum (FBS) (Gibco, USA) and 1% penicillin–streptomycin (Gibco, USA) and maintained at 37 °C in a 5% CO_2_ incubator. Cell seeding was subsequently performed, and once the cells were fully adherent, they were pretreated with 500 ng/mL lipopolysaccharide (LPS, Sigma-Aldrich, St. Louis, MO, USA) for 6 h to induce differentiation into M1-type macrophages (LPS group). They were then cultured with 100 ng/mL Reg3β (R&D, Minneapolis, MN, USA) for 24 h to promote differentiation into M2-type macrophages (LPS + Reg3β group). Untreated RAW264.7 cells served as the blank control group (control group). Transcriptome analysis of M1 and M2 macrophages was conducted using the RNA-seq technology before and after Reg3β treatment, and the differentially expressed genes were identified.

### 2.3. Cell Transfection and Grouping

A knockdown plasmid targeting NT5E (sh-NT5E) was constructed. When RAW264.7 cells cultured in six-well plates reached approximately 60% confluence, sh-NT5E was transfected into the cells using Lipofectamine™ 2000 (Invitrogen Life Technologies, Carlsbad, CA, USA), following the manufacturer’s instructions. Six sterile centrifuge tubes were prepared and divided into two groups: A and B. Each tube was filled with 250 μL of DMEM basal medium. Then, 5 μL of Liopofectamine™ 2000 was added to each tube in group A, which were allowed to stand for 5 min. Separately, 5 μL of sh-NT5E plasmid was added to each tube in group B. The contents of each tube in group B were then added dropwise to the corresponding tubes in group A and incubated for 20 min. Meanwhile, the culture medium in the six-well plates was discarded, and the wells were washed twice with basal medium. Thereafter, 2 mL of fresh basal medium was added to each well. After the incubation period, the A + B mixture was slowly added to the wells, mixed thoroughly, and incubated in a CO2 incubator. Eight hours later, the medium was replaced with complete medium without antibiotics. Cells were divided into six groups: normal control (control), sh-NT5E + normal control (sh-control), LPS + normal control (LPS), LPS + sh-NT5E + normal control (LPS + sh-NT5E), LPS + Reg3β + normal control (LPS + Reg3β), and LPS + Reg3β + sh-NT5E + normal control (LPS + Reg3β + sh-NT5E).

### 2.4. Single-Cell RNA-Seq Analysis

Following a comprehensive transcriptomic analysis, we further investigated the potential cellular mechanisms underlying myocarditis using scRNA-seq data from the GSE142564 dataset [26]. This analysis was conducted with the Seurat package (v5.1.0) [27]. Standard workflows were applied for normalization, identification of variable features, and data scaling, all using default parameters. Clusters were identified using the “FindClusters” function in Seurat v5, and dimensionality reduction was performed via Uniform Manifold Approximation and Projection [28]. Marker genes were identified using the “FindAllMarker” function. Cells were annotated based on known lineage-specific marker genes, including those for M1 macrophages, M2 macrophages, NK cells, and B cells. Afterward, stacked bar graphs were used to illustrate the proportion of each cell type across different time points, whereas dot plots were used to display the expression of myocarditis-related genes in different cell types over time.

### 2.5. Western Blot Detection of Relevant Protein Expression Levels

Cells from each treatment group were harvested and lysed in protein-extraction buffer, and the samples were centrifuged at 4 °C and 12,000 rpm for 5 min. Sample volumes were adjusted, and the proteins were subjected to 10% and 7.5% sodium dodecyl sulfate–polyacrylamide gel (SDS–PAGE) electrophoresis, wet-transferred onto PVDF membranes, and incubated with 5% skimmed milk for 2 h. Samples were then incubated overnight at 4 °C with primary antibodies (1:1000), including anti-GAPDH (mouse, 1:5000; Proteintech, Rosemont, IL, USA), anti-Arg1 (rabbit, 1:1000; Cell Signaling Technology, Danvers, MA, USA), anti-iNOS (rabbit, 1:1000; Cell Signaling Technology), and anti-NT5E (rabbit, 1:1000; Cell Signaling Technology). After the cells were incubated with the corresponding secondary antibodies (1:5000), they were incubated for two hours at room temperature. Membranes were developed using ECL luminescent substrate (ECL, Pierce Corporation, Appleton, WI, USA); signal detection was performed with a luminescent imaging system; and grayscale quantification was conducted using ImageJ software (v1.53).

### 2.6. qRT–PCR Assay (Q-PCR)

Total RNA was extracted from each treatment group using the TRIzol method (Vazyme, Shanghai, China). cDNA synthesis was performed using a reverse-transcription kit (Thermo Fisher Scientific, Carlsbad, CA, USA), following the manufacturer’s instructions. cDNA amplification was then performed via quantitative PCR (qPCR) using a fluorescence-based kit (Thermo Fisher Scientific, USA), following the manufacturer’s instructions. Primer sequences are listed below in Table 1. The relative gene expression levels were calculated using the 2^−ΔΔct^ method.

### 2.7. MR Analysis

For both forward and reverse MR analyses, we employed the inverse-variance weighted (IVW) method as the principal analysis to assess the causal relationship between plasma protein levels and myocarditis [29]. For plasma proteins instrumented by a single SNP, the Wald ratio method was applied to generate effect estimates [30]. For those instrumented by two or more SNPs, the IVW method was primarily used. pQTLs were selected based on the following criteria: (1) genome-wide significance threshold of *p* < 5 × 10^−8^ to identify highly correlated SNPs with plasma proteins; (2) LD clumping using the 1000 Genomes Project European reference panel to identify independent pQTLs for each protein (r2 < 0.001); and (3) R2 and F-statistics (R2 = 2 × EAF × (1 − EAF) × beta2; F  =  R2 × (N − 2)/(1 − R2)) to assess instrument strength. Instruments with F-statistics less than 10 were considered weak and excluded [31].

In the reverse analysis, SNPs linked to myocarditis were identified using a genome-wide significance threshold (*p* < 5 × 10^−5^). LD clustering (r2 < 0.001) was applied to minimize correlations among instruments. All analyses were performed in R (v4.4.2) using the TwoSampleMR (v0.5.7) software package. This study strictly adhered to the Strengthening the Reporting of Observational Studies in Epidemiology using Mendelian Randomization (STROBE-MR) guidelines (Table S3). All data were publicly available, and ethical approval and informed consent were obtained from the original studies.

### 2.8. Mediation Analysis

Using GWAS summary data, a two-step mediated MR analysis was conducted to determine whether immune cells act as the intermediate factors through which plasma proteins affect myocarditis progression. The MR analysis followed the same methodology as described above. Mediated MR was used to estimate the proportions of protein effects on myocarditis through immune cells. Indirect effects were calculated using the formula β1*β2, while direct effects were determined by subtracting indirect effects from the total effects [32]. This method accounts for potential measurement errors in the estimates. Mediation and total effects were clearly distinguished in all analyses [33].

### 2.9. GO and KEGG Enrichment Analysis

The ClusterProfiler(v4.10.0) package [34] was used to perform Gene Ontology (GO) and Kyoto Encyclopedia of Genes and Genomes (KEGG) enrichment analyses on proteins with a *p*-value < 0.01.

### 2.10. Protein–Protein Interaction (PPI) Network Construction and Core Gene Screening

The STRING database, a free online resource for identifying and predicting protein interactions, was used to construct a PPI network of shared myocarditis-related proteins [35,36]. Cytoscape software (v3.9.0) was used to visualize the network, and hub genes were identified using the CytoHubba plugin (v0.1) [37].

### 2.11. Drug Screening

Evaluating interactions between proteins and drugs is a critical step in determining whether a target protein can serve as a viable drug target. In this study, we used the Drug Signatures Database (DSigDB), http://dsigdb.tanlab.org/DSigDBv1.0/, 2025 (accessed on 1 February 2025) [38]. DSigDB includes over 22,500 gene sets, more than 17,000 compounds, and nearly 20,000 genes, enabling comprehensive gene–drug association analysis. It is a valuable resource for drug–target research and drug discovery. Core gene information was uploaded to DSigDB to predict candidate drugs targeting those genes, providing a theoretical basis for gene-targeted therapy. Building on this, we conducted drug enrichment analysis using the R ClusterProfiler package. Significance thresholds were set at *p* > 0.05 and adjusted *p* (*p*.adjust) > 0.05. The enrichment analysis employed the hypergeometric test method to evaluate whether core genes were significantly enriched in the drug–target gene sets. Significant results (*p* > 0.05) were visualized using bar charts and gene–drug interaction network diagrams.

### 2.12. Molecular Docking Analysis

To better understand the potential of candidate drugs to bind target proteins, molecular docking was performed to evaluate binding affinity and interaction modes. Based on previous drug enrichment analysis results, six candidates were selected for docking. Protein structures were retrieved from the Protein Data Bank [39] https://www.uniprot.org/, 2025 (accessed on 1 February 2025) with ligands removed. Drug structures were obtained from ZINC15 [40] http://zinc20.docking.org/, 2025 (accessed on 1 February 2025) and PubChem Small-Molecule Ligand Database https://pubchem.ncbi.nlm.nih.gov/, 2025 (accessed on 1 February 2025). PyMol software (v3.0.5) [41] was used for docking and visualization, and the binding free energy was calculated.

### 2.13. PheWAS Analysis

To investigate the potential side effects of the 10 proteins associated with myocarditis, we performed PheWAS for various diseases [42]. We used the UK Biobank [43] to recruit a sample size of approximately 500,000 participants aged 40 to 70 years from across the UK and used summary statistics to analyze the impact of SNPs and their outcomes. The database contains detailed demographic data (sex, age, height, weight) as well as electronic medical records covering biomarkers, imaging data, hospitalization records, and healthcare interactions. Detailed information on phenotype sources, questionnaires, and measurement protocols is available from the UK Biobank official website https://biobank.ndph.ox.ac.uk/showcase/search.cgi, 2025 (accessed on 1 February 2025).

## 3. Results

### 3.1. RNA-Seq Analysis

**Figure 1:**
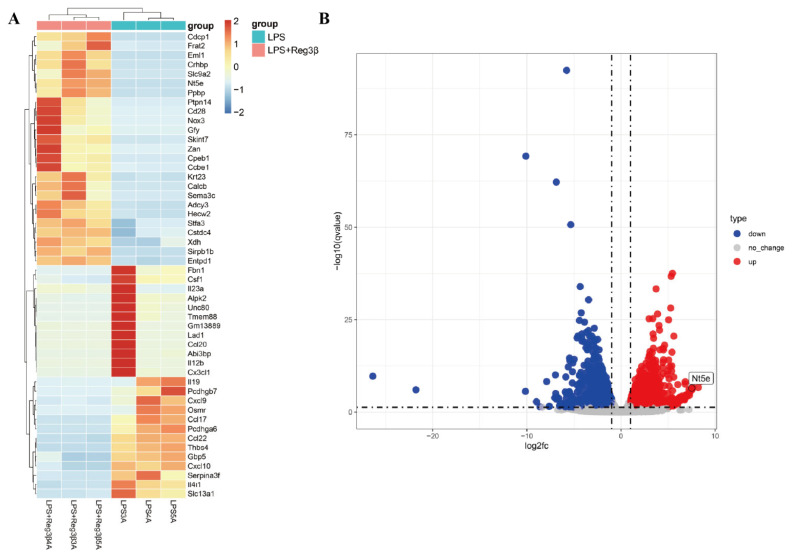
(**A**): Heatmap showing differentially expressed genes between the LPS group and the LPS + Reg3β group (*n* = 3); darker red indicates higher gene expression levels. (**B**): Volcano plot showing upregulated (red) and downregulated (blue) genes (*p*-value < 0.05).

A total of 3010 differentially expressed genes were detected in the transcriptome of RAW264.7 cells treated with or without Reg3β, among which 1623 genes were upregulated and 1387 were downregulated (Tables S4–S6). The heatmap (Figure 1A) shows the 50 genes with the most significant expression differences. The upregulated gene NT5E was particularly notable and prominently positioned in the volcano plot, as shown in Figure 1B.

### 3.2. Results of Single-Cell RNA-Seq Analysis

**Figure 2:**
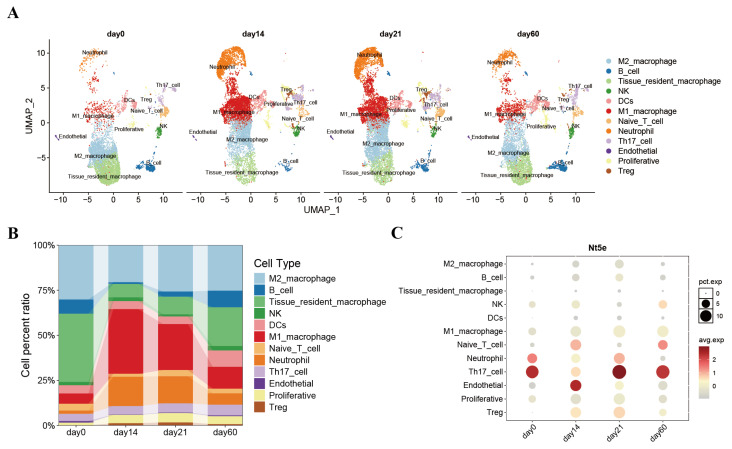
Cell clustering, composition dynamics, and NT5E expression over time. (**A**): UMAP visualization of single-cell transcriptomes on days 0, 14, 21, and 60. Cells are colored by annotated cell types. Distinct clusters correspond to immune and stromal populations, including macrophages, neutrophils, NK cells, T cells, B cells, and endothelial cells. (**B**): Stacked bar plot showing the proportion of each cell type over time. The colors of the cell types correspond to panel A. (**C**): Dot plot showing Nt5e expression in different cell types across time points.

We performed scRNA-seq analysis using GSE142564 to investigate myocarditis at the cellular level and to explore NT5E expression in M1 and M2 macrophages. Clustering analysis revealed 26 distinct cell clusters (Figure 2A). Macrophages were categorized into two clusters and, based on cell markers, annotated as M1 and M2 macrophages (Figure 2B). The development of myocardial inflammation in mice involved two phases: an inflammatory injury phase on day 14, characterized by increased M1 macrophages, and an inflammatory remission phase on day 21, characterized by increased M2 macrophages. NT5E expression increased in M2 macrophages during the inflammatory abatement phase, as shown in Figure 2C. Expression profiles of other core genes across the 26 cell populations are detailed in Figures S1 and S2.

### 3.3. Validation of Biomarkers by Western Blot (WB) and Quantitative Polymerase Chain Reaction (Q-PCR)

**Figure 3:**
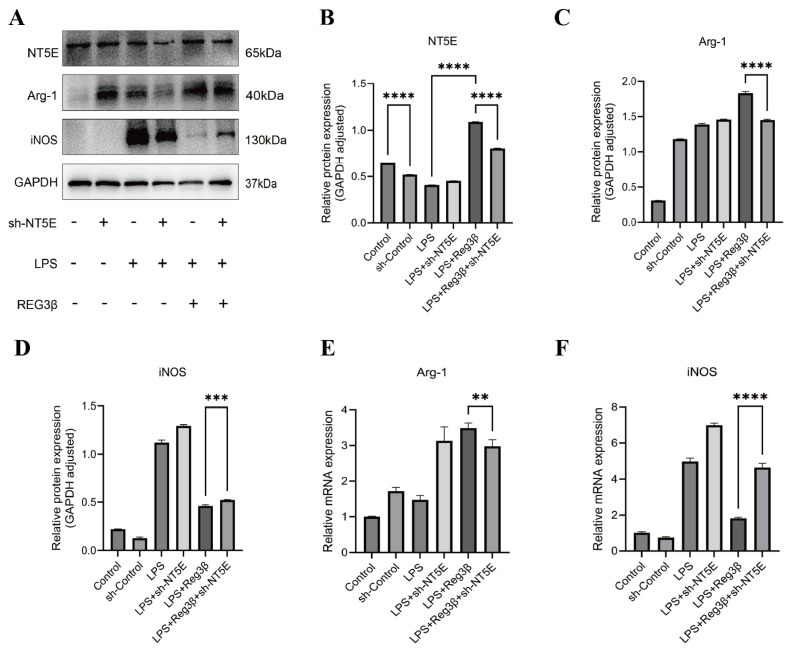
(**A**): Western blot analysis of NT5E, Arg-1, and iNOS protein expression in RAW264.7 cells post-NT5E knockdown. (**B**): Bar graph of NT5E expression. (**C**): Bar graph of Arg-1 expression. (**D**): Bar graph of iNOS expression. (**E**): RT-qPCR results for Arg-1. (**F**): RT-qPCR results for iNOS. Data were presented as mean ± SEM, *n* = 3; ** *p* < 0.01, *** *p* < 0.001, **** *p* < 0.0001.

The protein expression of NT5E, Arg-1, and iNOS following transfection with an NT5E knockdown plasmid in RAW267.4 cells is shown in Figure 3A. WB analysis demonstrated that Reg3β treatment increased NT5E expression (Figure 3B) and confirmed successful NT5E knockdown (Figure 3B). After NT5E knockdown, Reg3β failed to induce the M2 macrophage marker Arg-1 (Figure 3C) and attenuated the LPS-induced expression of the M1 macrophage marker iNOS (Figure 3D). RT-qPCR results were consistent with the protein data for Arg-1 (Figure 3E) and iNOS (Figure 3F) (Table S7). These findings suggest that NT5E plays a critical role in Reg3β-induced M2 polarization and that NT5E inhibition interferes with macrophage reprogramming from M1 to M2.

### 3.4. Identification of Candidate Proteins Associated with Myocarditis

**Figure 4:**
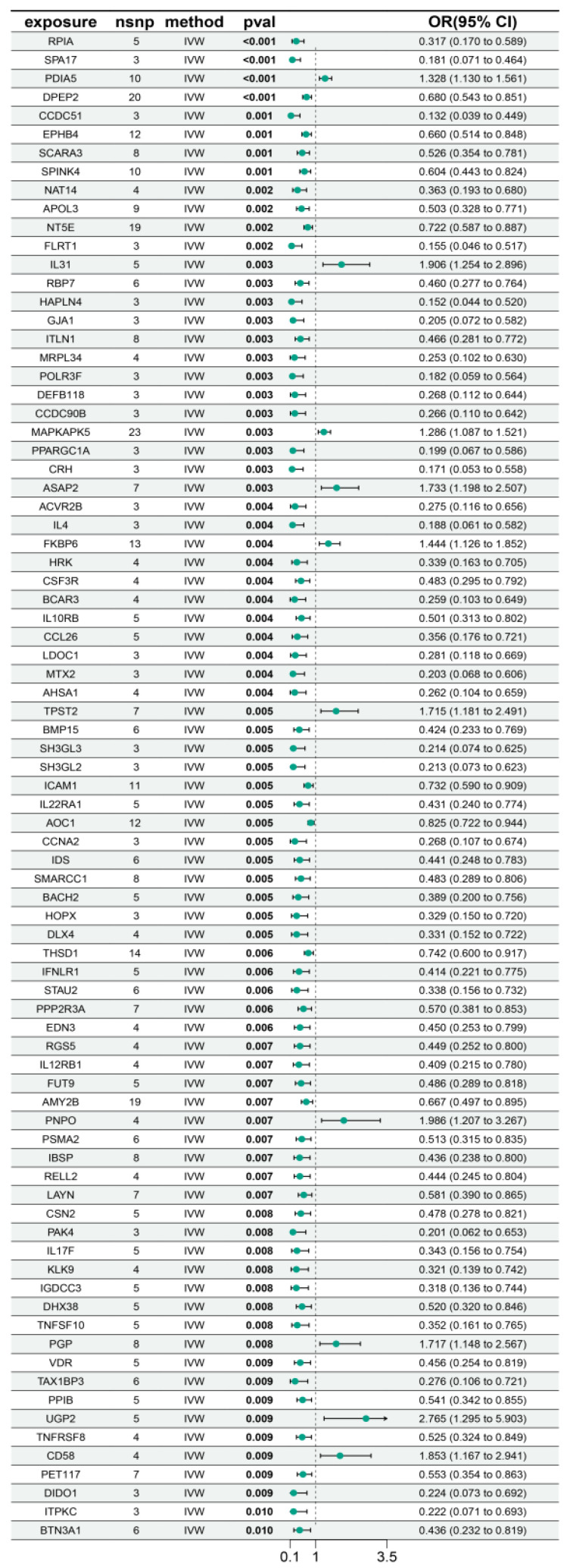
MR analysis results of pQTL in myocarditis.

**Figure 5:**
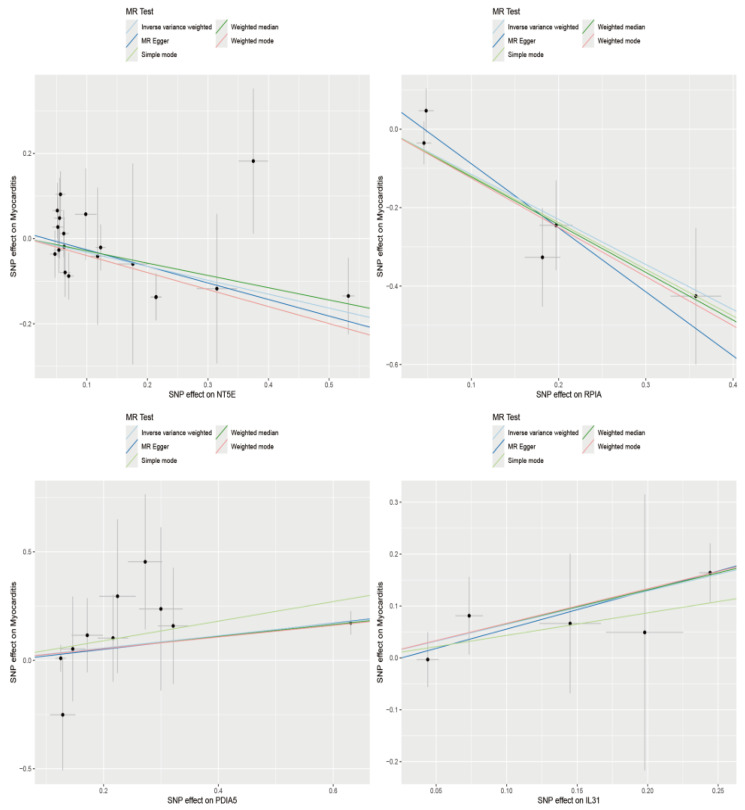
Scatter plot and regression curve from Mendelian randomization analysis showing causal associations between plasma proteins and myocarditis.

As shown in Figure 4, 81 proteins (*p* < 0.01) were identified by MR as causally associated with myocarditis development. Among them, NT5E [OR (95% CI): 0.722 (0.587–0.887), *p* = 0.002] showed a protective effect against myocarditis. Similarly, RPIA [OR (95% CI): 0.317 (0.170−0.589), *p* = 2.84 × 10^−4^] was also protective, whereas PDIA5 [OR (95% CI): 1.328 (1.130−1.561), *p* = 5.88 × 10^−4^] and IL31 [OR (95% CI): 1.906 (1.254−2.896), *p* = 0.003] were risk factors for myocarditis (Figure 5). These findings suggest that altered expression of certain proteins may serve as biomarkers for myocarditis. The detailed results of the MR analysis are presented in Table S8 and Figures S3–S6.

### 3.5. Mediation Analysis Results

**Figure 6:**
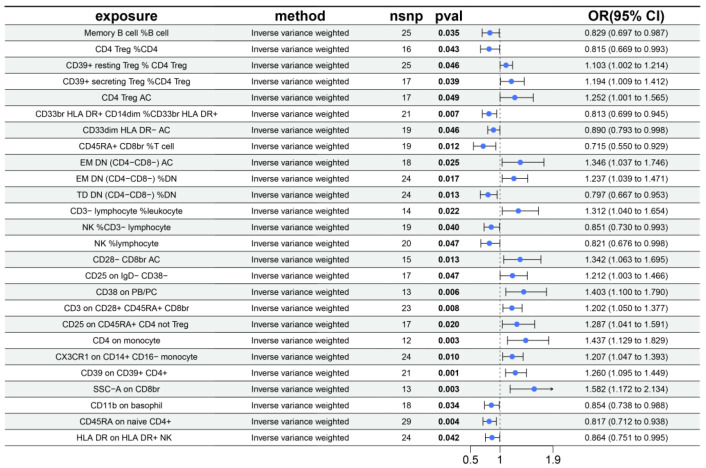
MR analysis results of immune cells in myocarditis.

**Figure 7:**
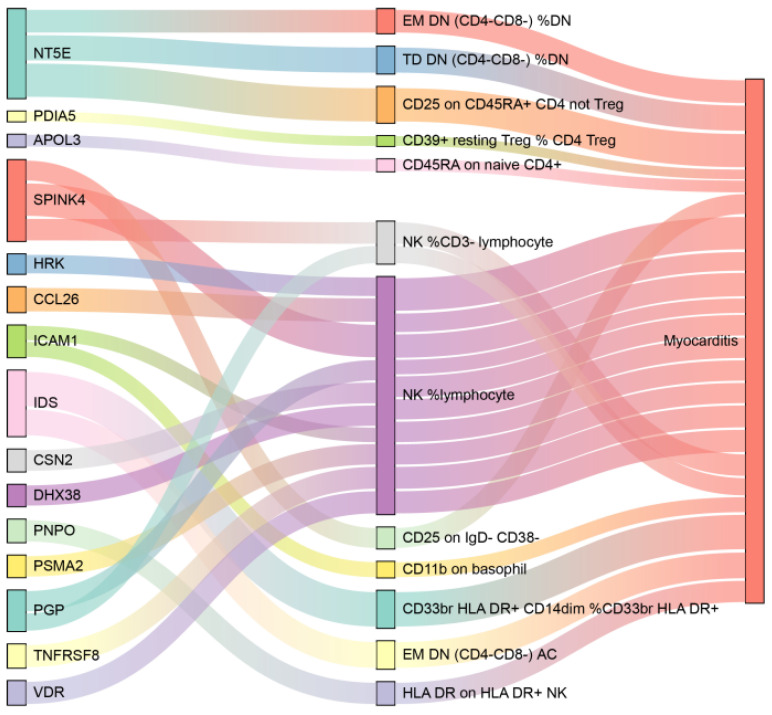
Proteins affect myocarditis through immune cells.

Eighty-one proteins with strong causal associations with myocarditis were selected for mediation analysis. Reverse MR analysis excluded 5 proteins due to evidence of reverse causality, focusing the subsequent analyses on 76 proteins (Table S9). Using the IVW approach, 26 immune-cell phenotypes were identified as associated with myocarditis (Figure 6). The detailed results of the MR analysis are presented in Table S10 and Figures S7–S10. Twenty mediation relationships involving 15 proteins and 12 immune-cell phenotypes were identified (Figure 7). Three immune cells mediated the effect of NT5E on myocarditis. Notably, the proportion of NK cells among lymphocytes mediated the effects of ten plasma proteins, including SPINK4, HRK, and CCL26. The results showed that the mediating effect of plasma proteins through immune cells accounted for their effect on myocarditis, as detailed in Tables S11 and S12.

### 3.6. Enrichment Analysis

**Figure 8:**
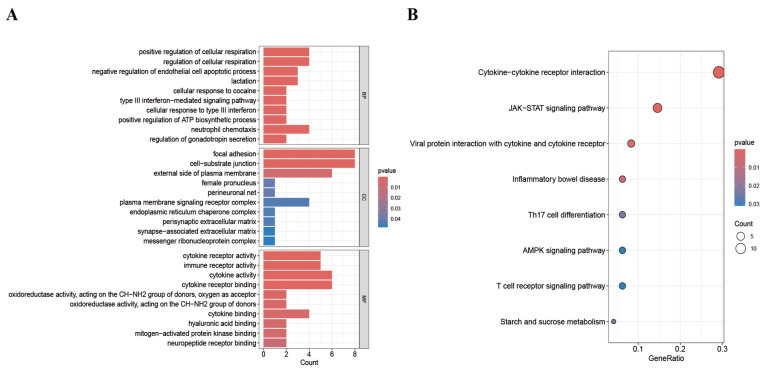
(**A**): GO enrichment results for three terms. (**B**): KEGG enrichment results.

Gene Ontology (GO) and Kyoto Encyclopedia of Genes and Genomes (KEGG) analyses were performed on the 81 MR-identified proteins to elucidate the molecular mechanisms underlying their roles. GO enrichment analysis using the Metascape database yielded 1223 significant entries (*p* < 0.01), including 1005 for biological processes (BPs), 77 for cellular components (CCs), and 141 for molecular functions (MFs). The top 10 GO terms, ranked by *p*-value, were selected for visualization. The results revealed that, in terms of biological processes, proteins were mainly enriched in the positive regulation of cellular respiration and biosynthetic processes. For cellular components, proteins were mainly enriched in focal adhesion and cell–substrate junctions. Regarding molecular functions, enrichment was mainly observed in cytokine activity and cytokine receptor binding (Figure 8A). Eight EKGG pathways were similarly selected based on their *p*-values for visualization (Figure 8B). The KEGG analysis revealed that the target proteins were primarily enriched in the following pathways: cytokine–cytokine receptor interaction, the JAK-STAT signaling pathway, and viral protein interaction with cytokines and cytokine receptors.

### 3.7. PPI Network Construction and Core Gene Screening

**Figure 9:**
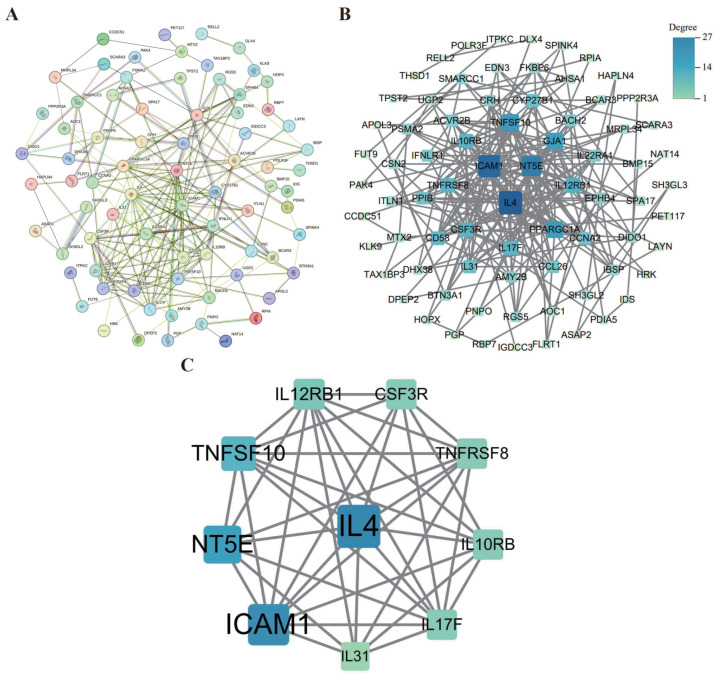
PPI network construction diagram. (**A**): PPI network constructed using STRING. (**B**): Full PPI network visualization. Key clusters with hub genes are highlighted in blue. (**C**): Core subnetwork highlighting top hub genes.

We used the STRING database—the widely recognized database for its precision in mapping PPIs—to construct a comprehensive PPI network for 81 identified proteins. The network was visualized in Cytoscape, and node degree (gene) was used to rank and select the top 10 hub genes: IL4, ICAM1, NT5E, IL17F, IL12RB1, TNFSF10, TNFRSF8, CSF3R, IL10RB, and IL31 (Figure 9).

### 3.8. Screening for Candidate Drugs

**Figure 10:**
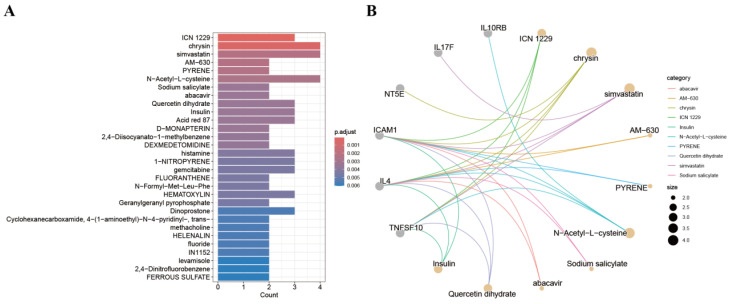
(**A**): Bar chart of drug prediction results. (**B**): Gene–drug interaction network diagram.

Potential therapeutic compounds targeting the core genes were predicted using DSigDB. Enrichment analysis (adjusted *p* < 0.05) identified candidate drugs including ICN 1229, chrysin, simvastatin, AM-630, pyrene, and N-acetyl-L-cysteine (Table S13, Figure 10A,B).

### 3.9. Molecular Docking

**Figure 11:**
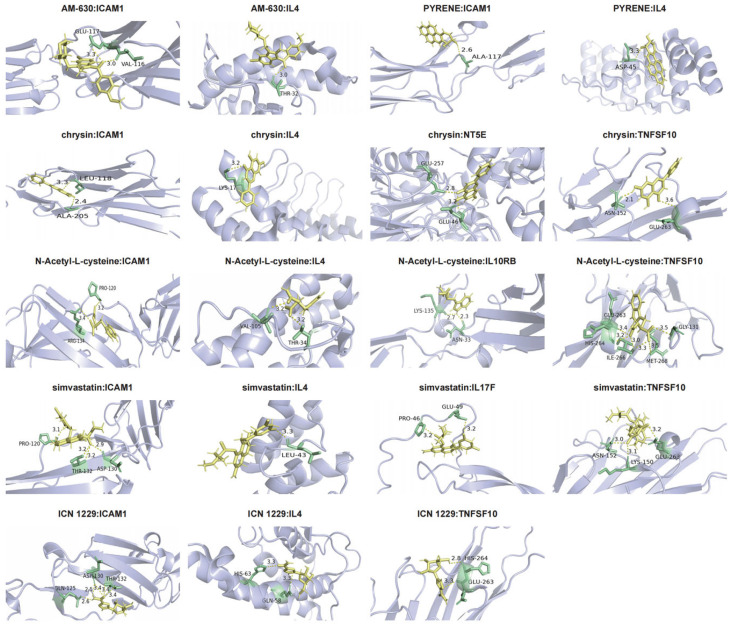
Molecular docking visualization between target proteins and drugs.

To assess drug–target binding affinities, molecular docking was performed for the six candidate drugs (Figure 11). In this analysis, chrysin demonstrated a stable interaction with NT5E (binding energy: −23.028145). Overall, drug–target binding energies ranged from −17.221935 to −27.784307 (Table 2), indicating favorable binding.

### 3.10. PheWAS Reveals the Potential Side Effects of Drugs Targeting Myocarditis-Related Proteins

**Figure 12:**
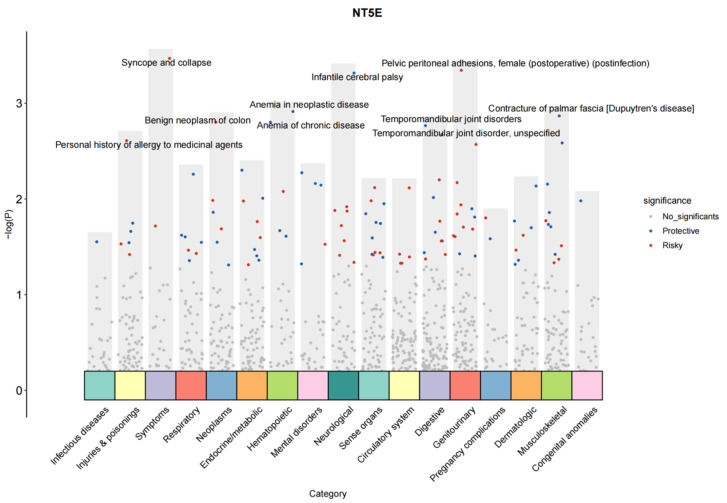
PheWAS analysis of NT5E proteins and other disease outcomes in UKB.

To assess the effects of ten proteins associated with myocarditis on various phenotypes, a PheWAS was conducted using the UK Biobank Database Phenotype Bank. A *p*-value < 0.05 was considered indicative of a potential causal association. Higher genetically determined plasma levels of NT5E were linked to a reduced risk of myocarditis risk but were associated with increased risks of other genitourinary diseases, such as pelvic peritoneal adhesions, female sex (postoperative; postinfection), and neoplasm-related conditions, including benign neoplasms of the colon (Figure 12). Plasma IL4 was identified as a protective factor against myocarditis and also showed associations with endocrine/metabolic diseases. For example, IL4 appeared to be protective against type 1 diabetes with ketoacidosis, diabetic polyneuropathy, and type 2 diabetes with neurological manifestations. In contrast, higher genetically determined plasma levels of IL31 were positively associated with an increased risk of myocarditis, as well as sensory organ disorders such as vitreous body disorders. In addition, IL31 was also found to be associated with pituitary hyperfunction, acromegaly, and gigantism (Figure S11).

## 4. Discussion

Myocardial inflammation occurs when immune cells [44], such as macrophages, T cells, B cells, and dendritic cells, accumulate in the heart. Two main types of macrophages exist in the heart: CCR2− and CCR2+ macrophages. CCR2−macrophages originate from the yolk sac during the embryonic period and are resident macrophages in the heart, where they play roles in processes such as phagocytosis and tissue repair and inhibit the aggregation of inflammatory cells. CCR2+ macrophages mainly originate from the bone marrow or the spleen and play a pro-inflammatory role [45]. As myocarditis progresses, the local microenvironment in the heart changes, and the macrophage phenotype shifts from inflammatory macrophages (M1 macrophages) to anti-inflammatory reparative macrophages (M2 macrophages) [10]. The release of Reg3β from injured cardiomyocytes at this time plays an important role in inducing the transition of M1 macrophages to M2 macrophages during programming. Studies have shown that NT5E can inhibit inflammation by promoting alternative macrophage activation (M2 phenotypic polarization). Both the WB and RT-qPCR results showed that, after NT5E was knocked down in RAW264.7 cells, Reg3β-induced M2-type macrophage polarization was inhibited. The expression of the M1-type macrophage marker iNOS was increased compared with that in the knockdown group, whereas the expression of the M2 macrophage marker Arg-1 was decreased. Single-cell analysis revealed that NT5E expression was increased in M2 macrophages.

These results indicate that Reg3β promotes M2 macrophage polarization through upregulation of NT5E, thereby inhibiting inflammation and promoting the repair of injured myocardium.

In this study, based on large-scale proteomics data, we identified multiple pathogenic proteins involved in myocarditis, including NT5E, via MR. NT5E and nine other proteins were further evaluated and identified as potential therapeutic targets. To explore the biological relevance of these targets, we performed enrichment analysis, mediator analysis, and PPI network analysis to identify core genes, as well as drug prediction and molecular docking. These results suggest that most of the predicted drugs play a role in inhibiting inflammatory factors, modulating immune cells, and antiviral processes, which are critical for myocarditis treatment. These findings extend the clinical applications of existing drugs and provide a theoretical basis for their use in the future treatment of myocarditis. In addition, the clinical diagnosis of myocarditis typically relies on the combined use of imaging, biopsy, and biomarkers. Cardiac magnetic resonance imaging (CMR) is widely used for non-invasive detection of myocardial inflammation and fibrosis, while endomyocardial biopsy (EMB) remains the gold standard, but its invasiveness limits its application. Cardiac troponin is commonly used to indicate myocardial injury, but it is not a specific marker for myocarditis. This study highlights the potential of NT5E and other plasma proteins as novel biomarkers that may reflect immune-regulatory mechanisms in myocarditis. Combining NT5E with existing tools such as CMR and troponin may improve diagnostic accuracy and provide additional insights into disease progression.

Extracellular-5′-nucleotidase (NT5E) is an immunosuppressive factor that establishes an adenosine-induced anti-inflammatory environment [46]. The above results suggest that NT5E participates in Reg3β-induced M2 macrophage polarization to promote myocardial injury repair [47]. Immune cells such as EM DN (CD4−CD8−) %DN, TD DN (CD4−CD8−) %DN, and CD25 on CD45RA+ CD4 non-Treg cells may also act as mediators influencing the onset and progression of myocarditis. Interleukin 4 (IL4) is a pleiotropic cytokine that binds to receptors to activate JAK-STAT6 to promote M2-type macrophage polarization [48]. Intercellular adhesion molecule-1 (ICAM-1) is a cell-surface glycoprotein and adhesion receptor expressed mainly in immune cells, endothelial cells, and epithelial cells; its expression is highly induced by several inflammatory factors [49]. Importantly, it has been reported in the literature that ICAM-1 promotes inflammation and that knocking it down reduces the symptoms of myocarditis; however, in this study, MR analysis revealed that ICAM-1 is a protective factor against myocarditis. A review of the relevant literature revealed that ICAM-1 may be related to the repair of damaged myocardium by recruiting endothelial progenitor cells in the late stage of myocarditis [50,51]. TNFSF10 (TRAIL) is a cytokine that plays a role in the regulation of apoptosis, and elevated TNFSF10 is associated with a favorable prognosis in myocarditis, which is also consistent with findings in cardiovascular disease [52,53]. Together with IL10RA, IL10RB constitutes the IL-10 receptor complex, which, upon binding to IL-10, activates Janus Kinase 1 (JAK1) and Tyrosine Kinase 2 (TYK2) and ultimately STAT3, inhibiting the production of pro-inflammatory cytokines and promoting anti-inflammatory responses [54]. TNFRSF8 (CD30) is a protein receptor located on the cell surface and belongs to the tumor necrosis factor receptor superfamily [55]. The TNFRSF8 molecule is a marker for the expression of Tregs, which are involved in suppressing inflammation [56]. Colony-Stimulating Factor 3 Receptor (CSF3R), also known as G-CSFR, is the gene encoding the G-CSF receptor [57]. The gene encodes a transmembrane protein called granulocyte colony-stimulating factor receptor, which provides proliferative signals to granulocytes and plays a key role in their proliferation and differentiation. CSF3R expression was positively correlated with IL22 and IL23 expression in patients with ulcerative colitis, and patients with higher CSF3R expression were also found to have enriched epithelial repair and regeneration gene profiles [58]. IL-31 is a helical cytokine belonging to the gp130/IL-6 cytokine family, which includes IL-6, viral IL-6, IL-11, IL-27, and leukemia inhibitory factor [59]. IL-31 induces pro-inflammatory effects in activated human monocytes and macrophages [60].

ICN 1229 (ribavirin) is a broad-spectrum antiviral drug that reduces viral replication by interfering with viral RNA synthesis [61]. It also modulates immune cells for anti-inflammatory effects [62]. Chrysin is a 5,7-dihydroxyflavone and a natural flavonoid found in plants [63]. It is present in large quantities in honey and propolis [64]. Chrysin inhibits the activity of Coxsackievirus B3 (CVB3) [65], the main virus that causes viral myocarditis. N-acetyl-L-cysteine is a potent antioxidant that inhibits inflammatory cardiovascular disease. It has shown promising results in alleviating both viral and autoimmune myocarditis [66,67]. The molecular docking results showed that the binding energy of chrysin to NT5E was −23.028145 kcal/mol, indicating that the binding interaction was highly stable.

Despite the significant progress made in this study, some limitations remain. First, the data in this study focused only on European populations, so data from other populations are needed for further validation, and the limited dataset is also a drawback of this study. Second, this study did not include subtype stratification analysis for myocarditis, which may have introduced a degree of confounding bias. Although ICAM1 and IL10RB have anti-inflammatory effects, they exhibit pro-inflammatory effects in a variety of diseases, which may be unfavorable for myocarditis. Therefore, their specific mechanisms of expression in myocarditis need to be further investigated. In addition, limitations of single-cell sequencing, such as dataset selection, may result in an incomplete representation of the phenotypic and functional characteristics of a particular cell type or subpopulation. Despite the identification of potential drug targets, their clinical efficacy remains uncertain and needs to be validated through experimental studies and clinical trials. Examples include in vivo validation using a mouse model of myocarditis to test top-ranked drug candidates. Addressing these limitations will enhance future research and improve our understanding of myocarditis and its therapeutic strategies.

## 5. Conclusions

We verified experimentally and via single-cell sequencing that NT5E is involved in the reg3β-induced polarization of M2-type macrophages and in the repair process of myocardial inflammatory damage. NT5E and various plasma proteins were identified as potential therapeutic targets for myocarditis by MR analysis, mediator analysis, and enrichment analysis. Through drug enrichment and molecular docking analyses, this study identified drug candidates, including those showing significant therapeutic potential in inhibiting inflammation and modulating signaling pathways. In addition, we utilized PheWAS to study protein-based related phenotypes, thereby deepening our understanding of their impact. However, further experimental studies and clinical trials are needed to validate the therapeutic potential of drugs targeting these proteins and to confirm their safety and efficacy in clinical settings. This study provides new insights and data to support future research on the precise diagnosis and treatment of myocarditis.

## Figures and Tables

**Table 1:** Primer sequences.

Primer Sequences	
GAPDH	Forward GGTCGGTGTGAACGGATTTG Reverse TGTAGACCATGTAGTTGAGGTCA
NT5E	Forward CAGCGATGACTCCACCAAGT Reverse CTCCGGCATCCAAAAACAGC
Arg-1	Forward CATTGGCTTGCGAGACGTAGAC Reverse GCTGAAGGTCTCTTCCATCACC
iNOS	Forward TGGAGCCAGTTGTGGATTGTC Reverse GGTCGTAATGTCCAGGAAGTAG

**Table 2:** Results of protein–drug molecular docking.

Drug	Target	Binding Energy (kcal/mol)
ICN 1229	TNFSF10	−18.793329
ICN 1229	IL4	−20.381943
ICN 1229	ICAM1	−17.373051
Chrysin	TNFSF10	−21.043499
Chrysin	IL4	−17.346796
Chrysin	ICAM1	−19.609625
Chrysin	NT5E	−23.028145
Simvastatin	TNFSF10	−20.781334
Simvastatin	IL4	−19.659033
Simvastatin	ICAM1	−17.294996
Simvastatin	IL17F	−27.784307
AM-630	IL4	−24.125502
AM-630	ICAM1	−21.320253
PYRENE	IL4	−20.845221
PYRENE	ICAM1	−17.221935
N-Acetyl-L-cysteine	IL10RB	−23.861942
N-Acetyl-L-cysteine	TNFSF10	−21.000706
N-Acetyl-L-cysteine	IL4	−22.408953
N-Acetyl-L-cysteine	ICAM1	−17.972317

## Data Availability

All data used in our study can be downloaded from the IEU open GWAS project (https://api.opengwas.io/ [accessed on 20 January 2025]), deCODE (https://www.decode.com/summarydata/ [accessed on 20 January 2025]), Gene Expression Omnibus (GEO) (http://www.ncbi.nlm.nih.gov/geo/ [accessed on 20 January 2025]), DSigDB (http://dsigdb.tanlab.org/DSigDBv1.0/ [accessed on 1 February 2025]), and UKBiobank (https://biobank.ndph.ox.ac.uk/showcase/search.cgi [accessed on 1 February 2025]).

## Supplementary Materials

The following supporting information can be downloaded at https://www.mdpi.com/article/10.3390/biology14081017/s1: Supplementary Material S1: Figure S1: Quality-control metrics and gene expression analysis of single-cell RNA-seq data across different time points. (A) Violin plots showing quality-control metrics for single-cell transcriptomes on days 0, 14, 21, and 60. (B) UMAP visualization of all cells colored by expression of the gene NT5E. Each dot represents a single cell. Red indicates cells with detectable NT5E expression. Figure S2. Dot plot showing expression of proteins across immune-cell types over time. Figure S3. Scatter plots of myocarditis as an outcome, using the proteins responsible for the mediating effect as exposure factors. Figure S4. Forest plots of myocarditis as an outcome using the proteins responsible for the mediating effect as exposure factors. Figure S5. Funnel plots of myocarditis as an outcome using the proteins responsible for the mediating effect as exposure factors. Figure S6. Leave-one-out sensitivity analysis with the proteins responsible for the mediating effect as exposure factors and myocarditis as outcome. Figure S7. Scatter plots for immune cells as exposure factors and myocarditis as outcome. Figure S8. Forest plots for immune cell as exposure factors and myocarditis as outcome. Figure S9. Funnel plots for immune cell as exposure factors and myocarditis as outcome. Figure S10. Leave-one-out sensitivity analysis for immune cell as exposure factors and myocarditis as outcome. Figure S11. PheWAS analysis of proteins and other disease outcomes in UKB. Supplementary Material S2: Table S1: The 4907 pQTLs. Table S2: The 731 immune phenotypes. Table S3: STROBE-MR checklist of recommended items to address in reports of Mendelian randomization studies. Table S4: Results of RNA-seq analysis. Table S5: Upregulated genes. Table S6: Downregulated genes. Table S7: Results of Q-PCR. Table S8: MR analysis results of pQTL on myocarditis. Table S9: Results of Re-MR analysis of myocarditis pQTLs. Table S10: MR analysis results of immune cells on myocarditis. Table S11: MR analysis results of pQTL on immune cells. Table S12: Mediated proportion. Table S13: Drug screening.

## Abbreviations

MR	Mendelian randomization
PheWAS	phenome-wide association studies
NT5E	extracellular-5′-nucleotidase
Reg3β	Regenerating islet-derived protein 3 beta
Arg-1	Arginase-1
Reg3α	Regenerating islet-derived 3 alpha
Reg3γ	Regenerating islet-derived 3 gamma
MyHC-α	α-myosin heavy chain
CK-MB	Creatine Kinase-MB
GWAS	Genome-wide association studies
pQTLs	protein quantitative trait loci
RNA-seq	RNA sequencing
GEO	Gene Expression Omnibus
SNPs	single nucleotide polymorphisms
IVs	instrumental variables
AC	absolute cell counts
RC	relative cell counts
MFI	median fluorescence intensities
MP	morphological parameters
FBS	fetal bovine serum
LPS	lipopolysaccharide
sh-NT5E	knockdown plasmid targeting NT5E
scRNA-seq	single-cell RNA sequencing
SDS–PAGE	sodium dodecyl sulfate-polyacrylamide gel
IVW	inverse variance weighted
GO	Gene Ontology
KEGG	Kyoto Encyclopedia of Genes and Genomes
PPI	Protein-protein interaction
DSigDB	Drug Signatures Database
WB	Western blotting
Q-PCR	quantitative polymerase chain reaction
iNOS	Inducible Nitric Oxide Synthase
SPINK4	Serine Peptidase Inhibitor Kazal Type 4
HRK	Harakiri
CCL26	C-C Motif Chemokine Ligand 26
BP	biological processes
CC	cellular components
MF	molecular functions
JAK-STAT	Janus Kinase-Signal Transducer and Activator of Transcription
STRING	Search Tool for the Retrieval of Interacting Genes/Proteins
CCR2	C-C Chemokine Receptor Type 2
RT–qPCR	Reverse-Transcription Quantitative Polymerase Chain Reaction
ICAM-1	Intercellular adhesion molecule-1
TRAIL	Tumor Necrosis Factor Superfamily Member 10
IL10RA	Interleukin-10 Receptor Alpha
IL10RB	Interleukin-10 Receptor Beta
JAK1	Janus Kinase 1
TYK2	Tyrosine Kinase 2
STAT3	Signal Transducer and Activator of Transcription 3
TNFRSF8	Tumor Necrosis Factor Receptor Superfamily Member 8
CSF3R	Colony-Stimulating Factor 3 Receptor
CVB3	Coxsackievirus B3
UKB	UK Biobank
ICN 1229	ribavirin
