# Supplementary material for: Mechanistic Study of NT5E in Reg3β-Induced Macrophage Polarization and Cooperation with Plasma Proteins in Myocarditis Injury and Repair
Source: Biology (Basel). 2025 Aug 7;14(8):1017. doi: 10.3390/biology14081017 (PMC12383428; doi:10.3390/biology14081017)
Supplement: Supplementary file 1 [file biology-14-01017-s001.zip › Supplementary data 1.pdf]

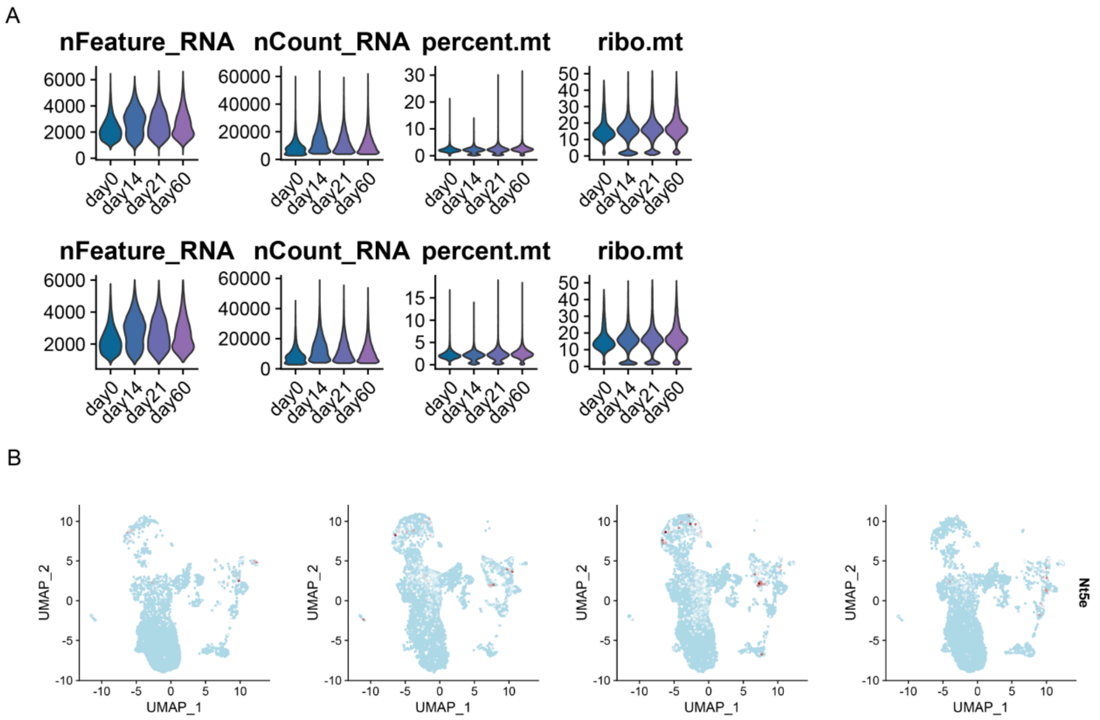

Figure S1. Quality control metrics and gene expression analysis of single-cell RNA-seq data across different time points. (A) Violin plots showing quality control metrics for single-cell transcriptomes at day 0, 14, 21, and 60. (B) UMAP visualization of all cells colored by expression of the gene NT5E. Each dot represents a single cell. Red indicates cells with detectable NT5E expression.

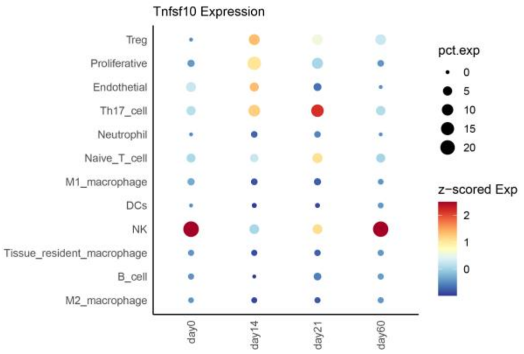

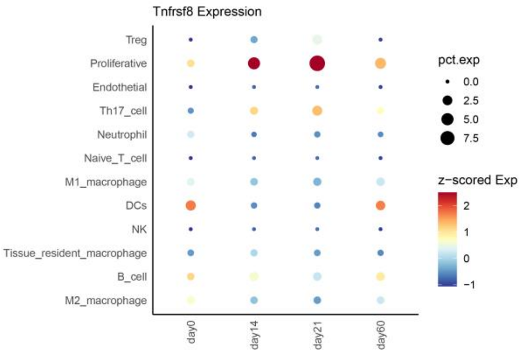

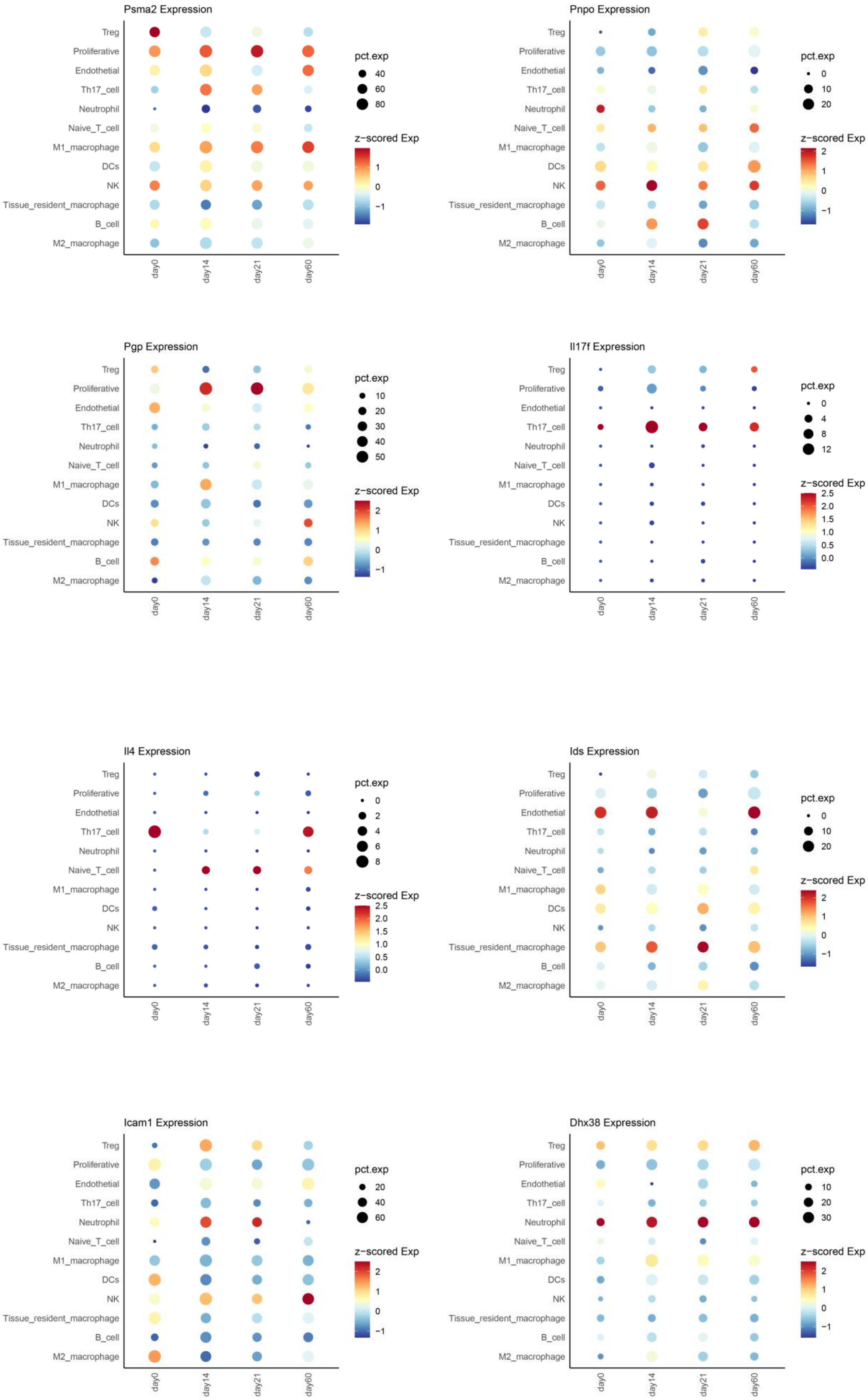
Psma2 Expression
Pnpo Expression
Pgp Expression
Il17f Expression
Il4 Expression
Ids Expression
Icam1 Expression
Dhx38 Expression
Treg
Proliferative
Endothetial
Th17_cell
Neutrophil
Naive_T_cell
M1_macrophage
DCs
NK
Tissue_resident_macrophage
B_cell
M2_macrophage
day0
day14
day21
day60
pct.exp
z−scored Exp

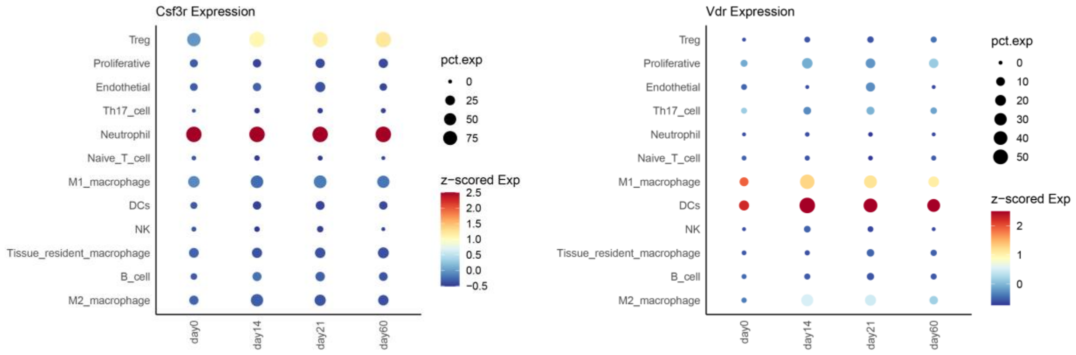

Figure S2. Dot plot showing expression of proteins across immune cell types over time.

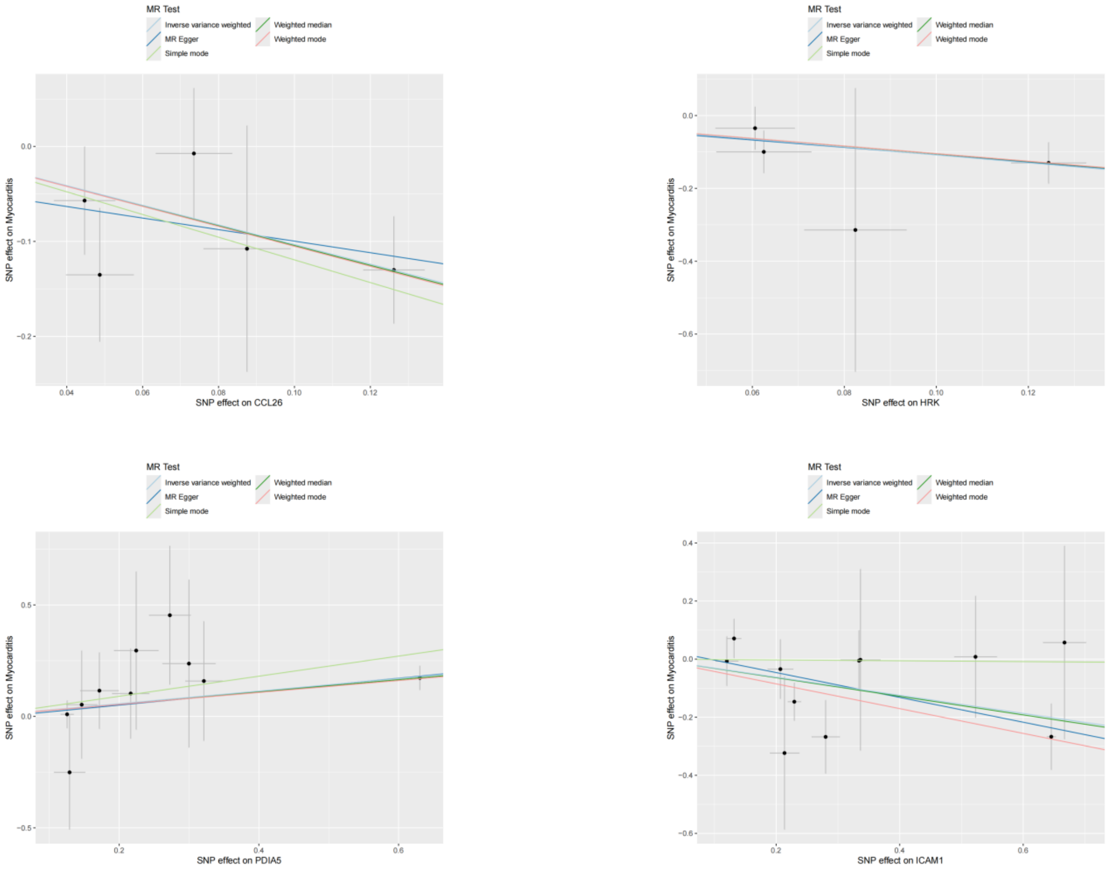

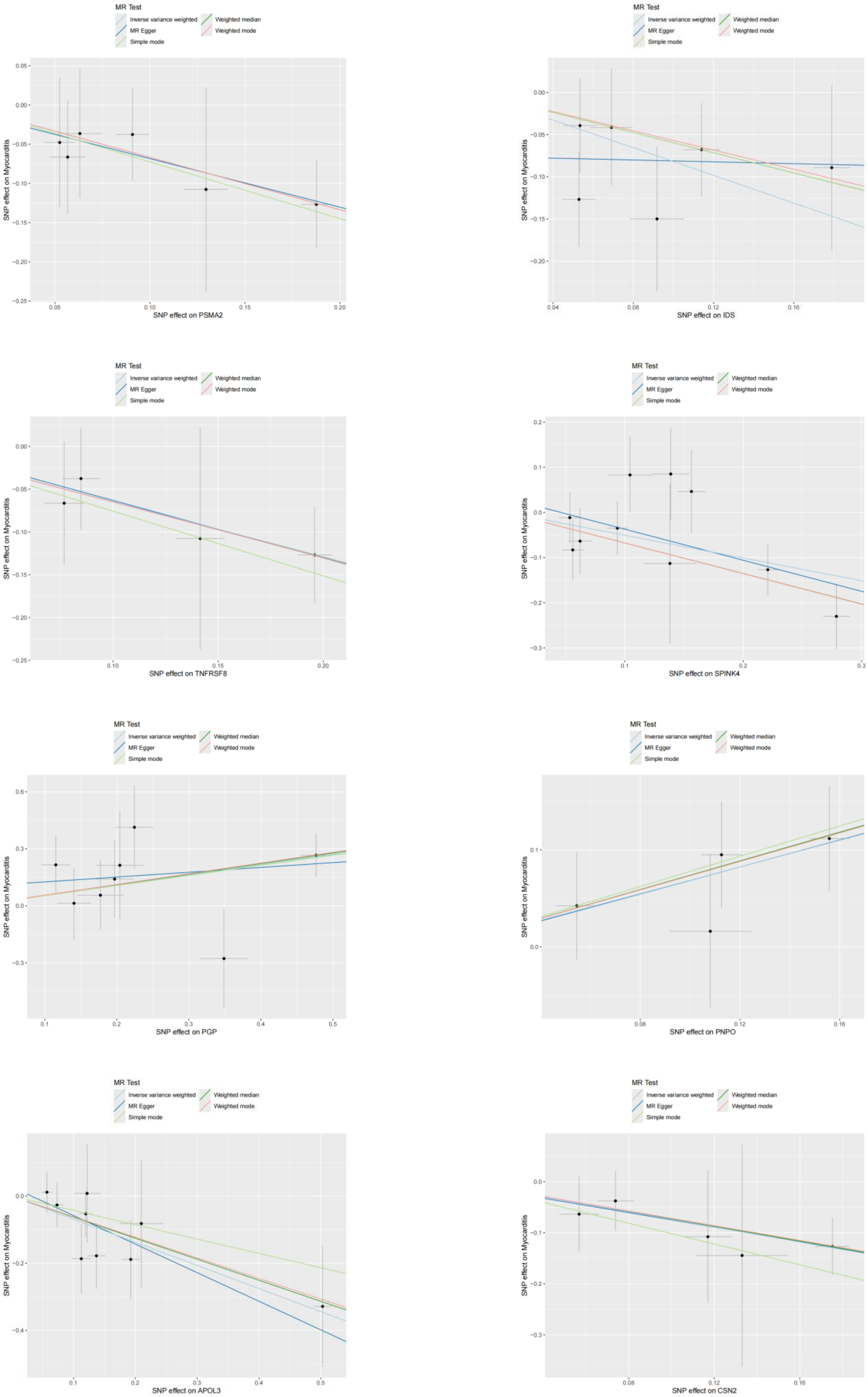
MR Test
Inverse variance weighted
MR Egger
Simple mode
Weighted median
Weighted mode
SNP effect on Myocarditis
SNP effect on PSMA2
SNP effect on IDS
SNP effect on TNFRSF8
SNP effect on SPINK4
SNP effect on PGP
SNP effect on PNPO
SNP effect on APOL3
SNP effect on CSN2

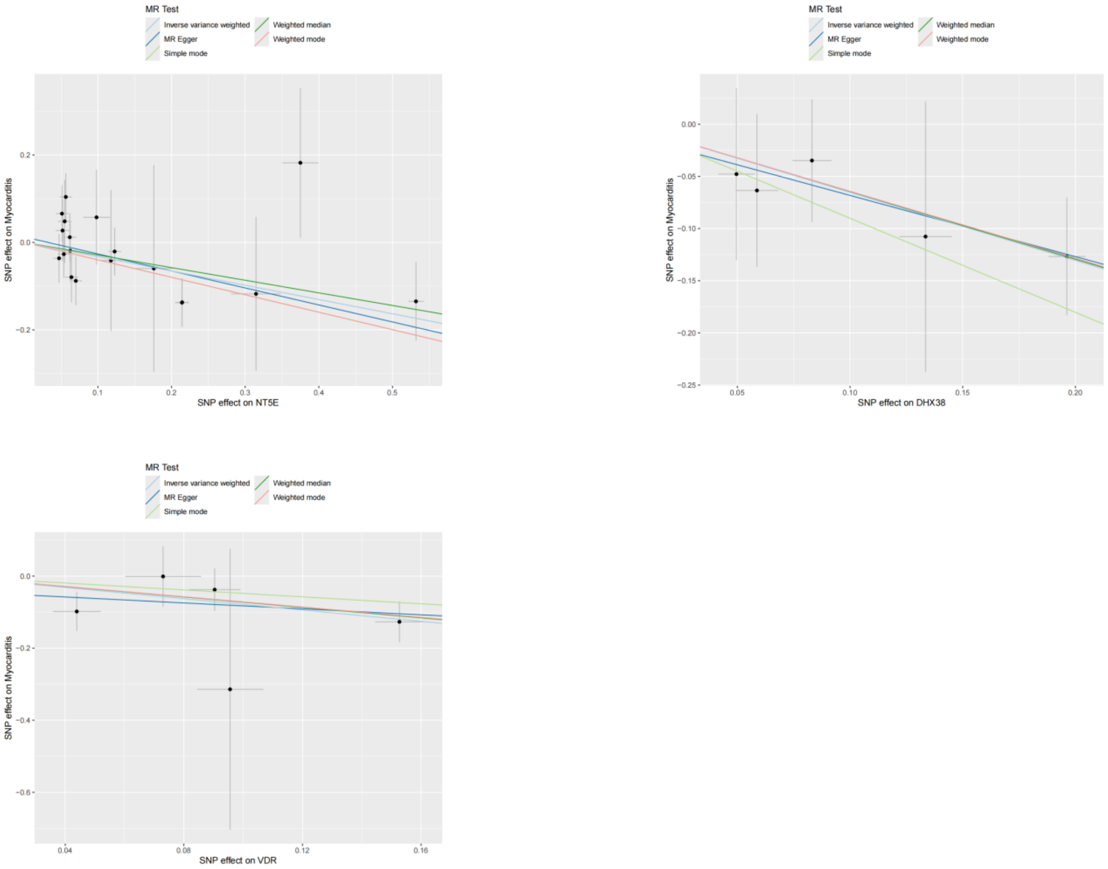

Figure S3. Scatter plots of myocarditis as an outcome using proteins mediating the mediating effect as exposure factors.

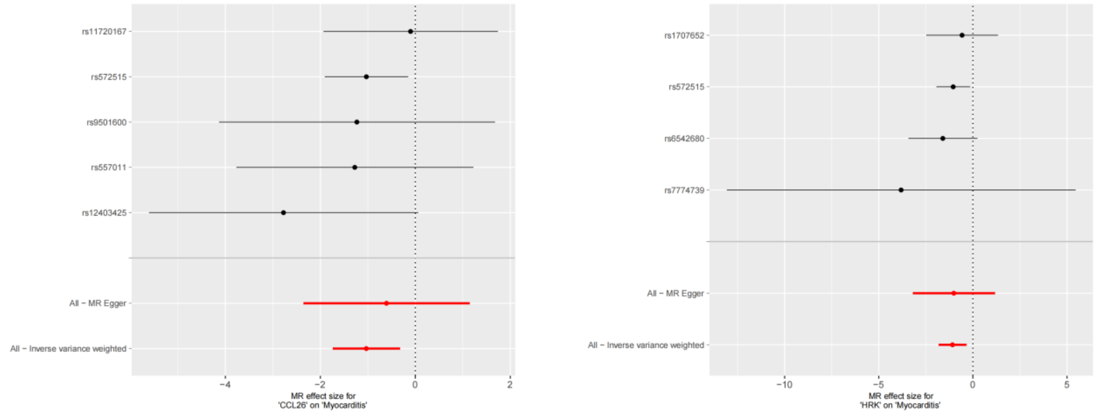

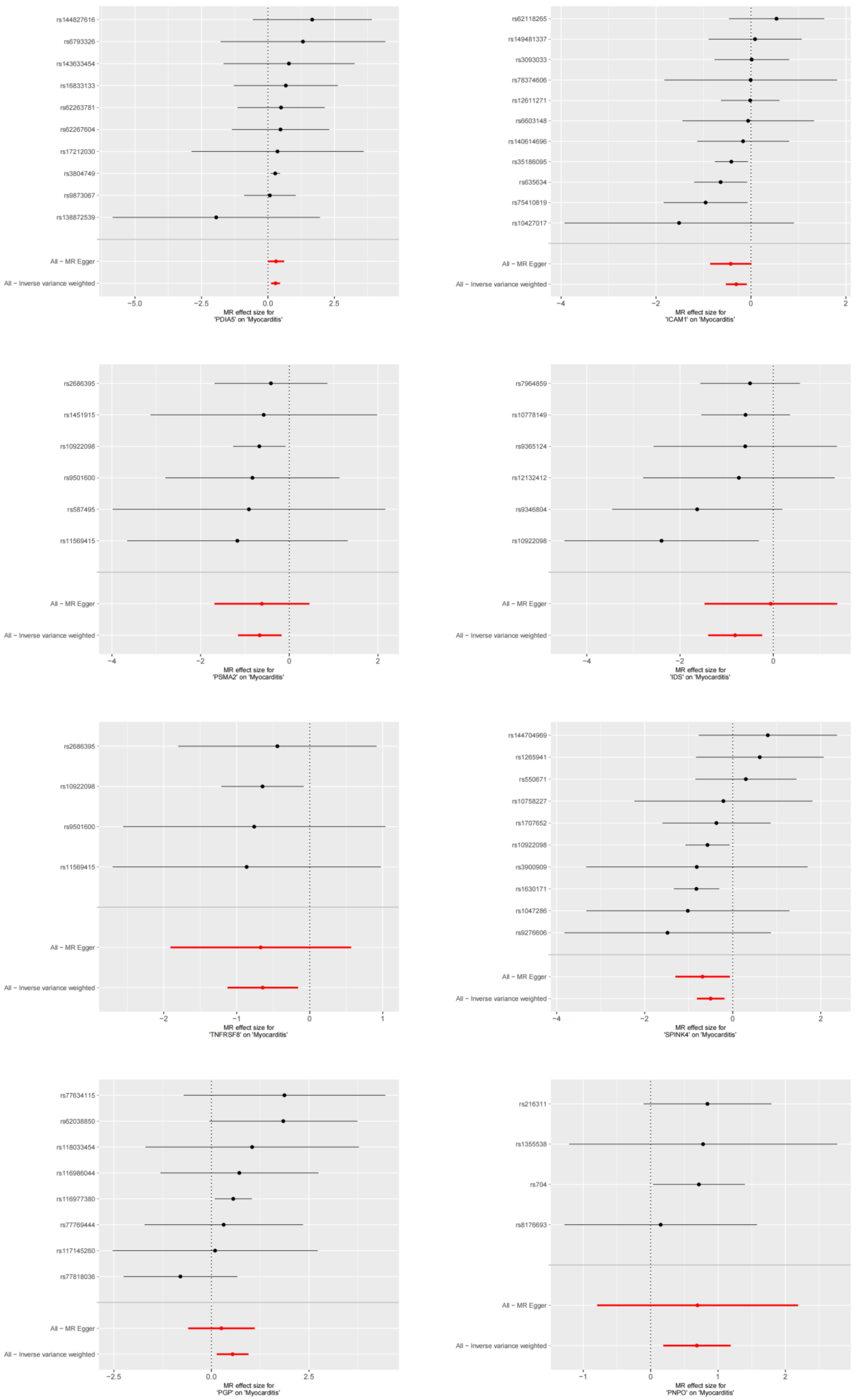
rs144827616
rs6793326
rs143633454
rs16833133
rs62263781
rs62267604
rs17212030
rs3804749
rs9873067
rs138872539
All − MR Egger
All − Inverse variance weighted
−5.0
−2.5
0.0
2.5
MR effect size for
'PDIA5' on 'Myocarditis'
rs62118265
rs149481337
rs3093033
rs78374606
rs12611271
rs6603148
rs140614696
rs35186095
rs635634
rs75410819
rs10427017
All − MR Egger
All − Inverse variance weighted
−4
−2
0
2
MR effect size for
'ICAM1' on 'Myocarditis'
rs2686395
rs1451915
rs10922098
rs9501600
rs587495
rs11569415
All − MR Egger
All − Inverse variance weighted
−4
−2
0
2
MR effect size for
'PSMA2' on 'Myocarditis'
rs7964859
rs10778149
rs9365124
rs12132412
rs9346804
rs10922098
All − MR Egger
All − Inverse variance weighted
−4
−2
0
MR effect size for
'IDS' on 'Myocarditis'
rs2686395
rs10922098
rs9501600
rs11569415
All − MR Egger
All − Inverse variance weighted
−2
−1
0
1
MR effect size for
'TNFRSF8' on 'Myocarditis'
rs144704969
rs1265941
rs550671
rs10758227
rs1707652
rs10922098
rs3900909
rs1630171
rs1047286
rs9276606
All − MR Egger
All − Inverse variance weighted
−4
−2
0
2
MR effect size for
'SPINK4' on 'Myocarditis'
rs77634115
rs62038850
rs118033454
rs116986044
rs116977380
rs77769444
rs117145260
rs77818036
All − MR Egger
All − Inverse variance weighted
−2.5
0.0
2.5
MR effect size for
'PGP' on 'Myocarditis'
rs216311
rs1355538
rs704
rs8176693
All − MR Egger
All − Inverse variance weighted
−1
0
1
2
MR effect size for
'PNPO' on 'Myocarditis'

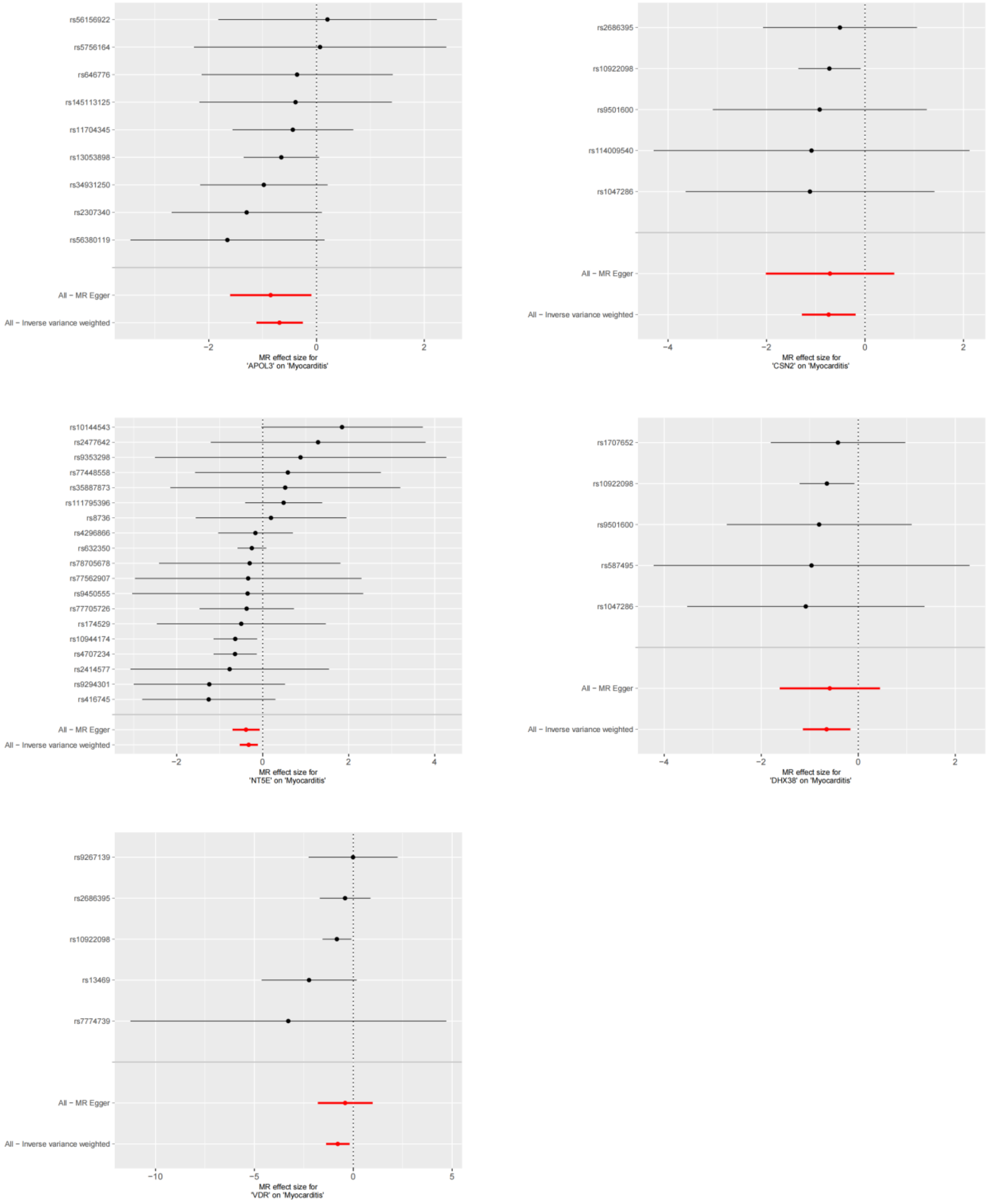

Figure S4. Forest plots of myocarditis as an outcome using proteins mediating the mediating effect as exposure factors.

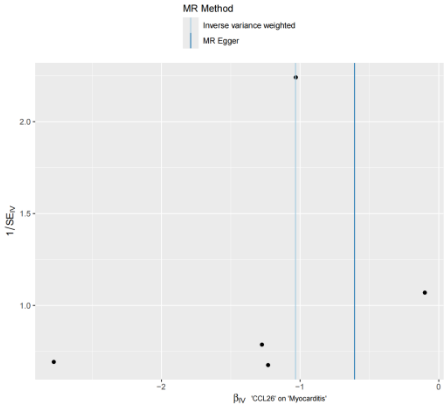
MR Method
Inverse variance weighted
MR Egger
1/SE_IV
β_IV 'CCL26' on 'Myocarditis'

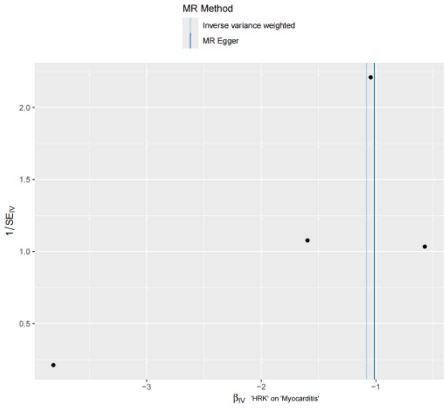
MR Method
Inverse variance weighted
MR Egger
1/SE_IV
β_IV 'HRK' on 'Myocarditis'

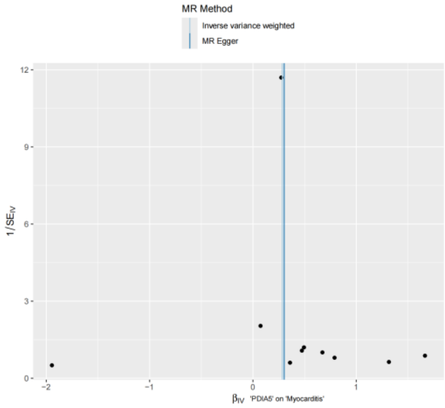
MR Method
Inverse variance weighted
MR Egger
1/SE_IV
β_IV 'PDIA5' on 'Myocarditis'

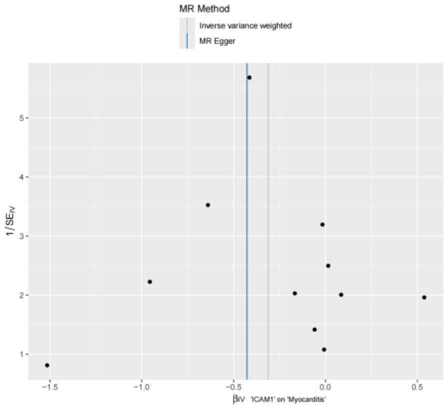
MR Method
Inverse variance weighted
MR Egger
1/SE_IV
β_IV 'ICAM1' on 'Myocarditis'

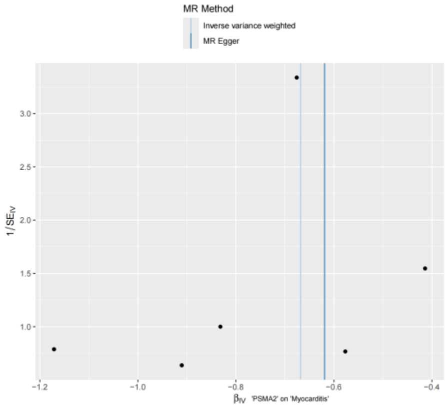
MR Method
Inverse variance weighted
MR Egger
1/SE_IV
β_IV 'PSMA2' on 'Myocarditis'

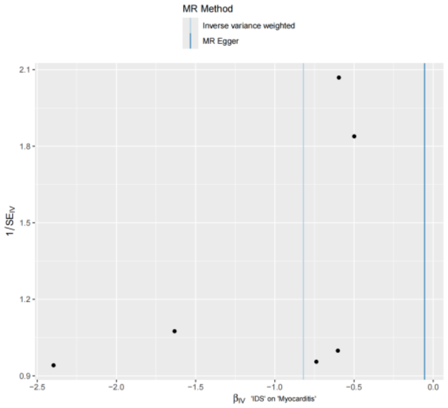
MR Method
Inverse variance weighted
MR Egger
1/SE_IV
β_IV 'IDS' on 'Myocarditis'

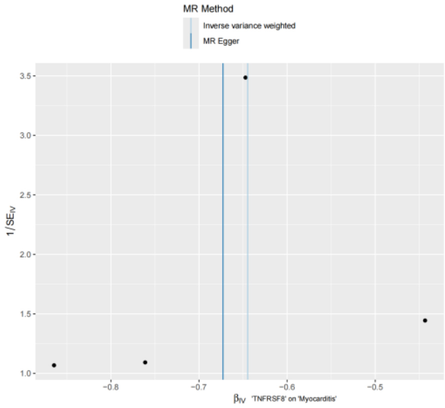
MR Method
Inverse variance weighted
MR Egger
1/SE_IV
β_IV 'TNFRSF8' on 'Myocarditis'

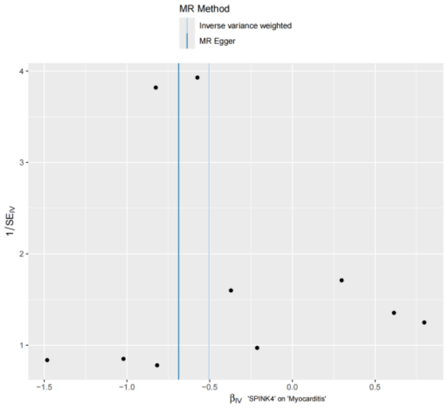
MR Method
Inverse variance weighted
MR Egger
1/SE_IV
β_IV 'SPINK4' on 'Myocarditis'

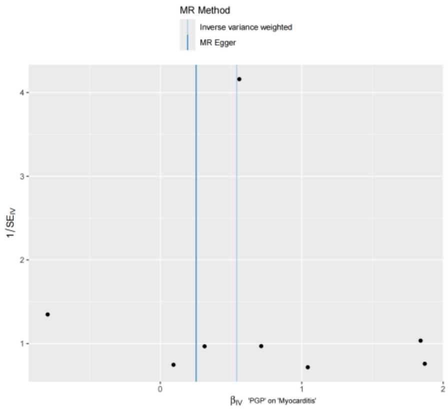
MR Method
Inverse variance weighted
MR Egger
1/SE$_{IV}$
β_{IV} 'PGP' on 'Myocarditis'

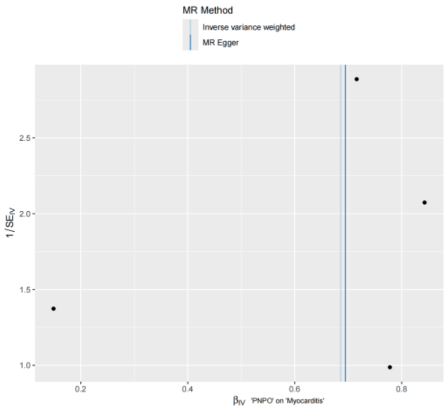
MR Method
Inverse variance weighted
MR Egger
1/SE$_{IV}$
β_{IV} 'PNPO' on 'Myocarditis'

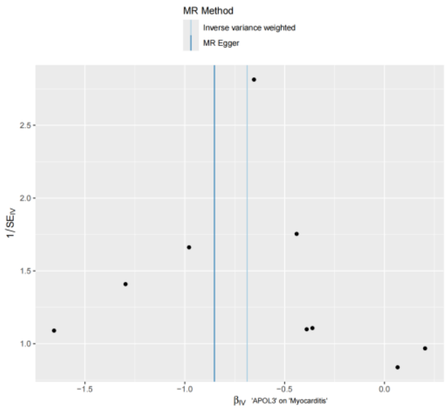
MR Method
Inverse variance weighted
MR Egger
1/SE$_{IV}$
β_{IV} 'APOL3' on 'Myocarditis'

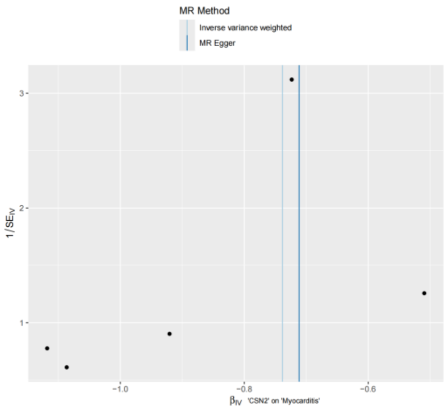
MR Method
Inverse variance weighted
MR Egger
1/SE$_{IV}$
β_{IV} 'CSN2' on 'Myocarditis'

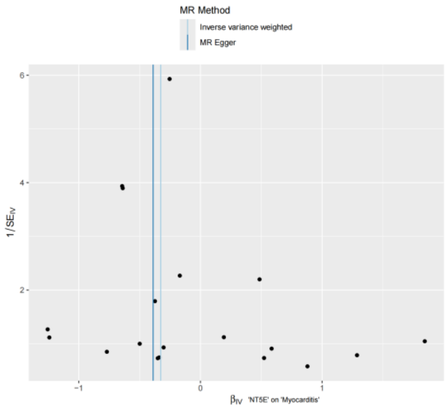
MR Method
Inverse variance weighted
MR Egger
1/SE$_{IV}$
β_{IV} 'NT5E' on 'Myocarditis'

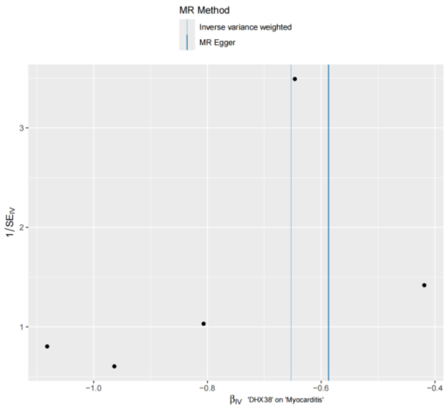
MR Method
Inverse variance weighted
MR Egger
1/SE$_{IV}$
β_{IV} 'DHX38' on 'Myocarditis'

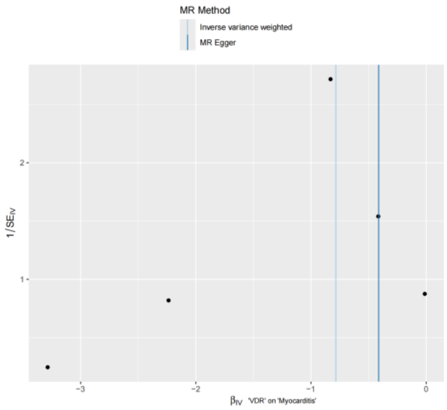
MR Method
Inverse variance weighted
MR Egger
1/SE$_{IV}$
β_{IV} 'VDR' on 'Myocarditis'

Figure S5. Funnel plots of myocarditis as an outcome using proteins mediating the mediating effect as exposure factors.

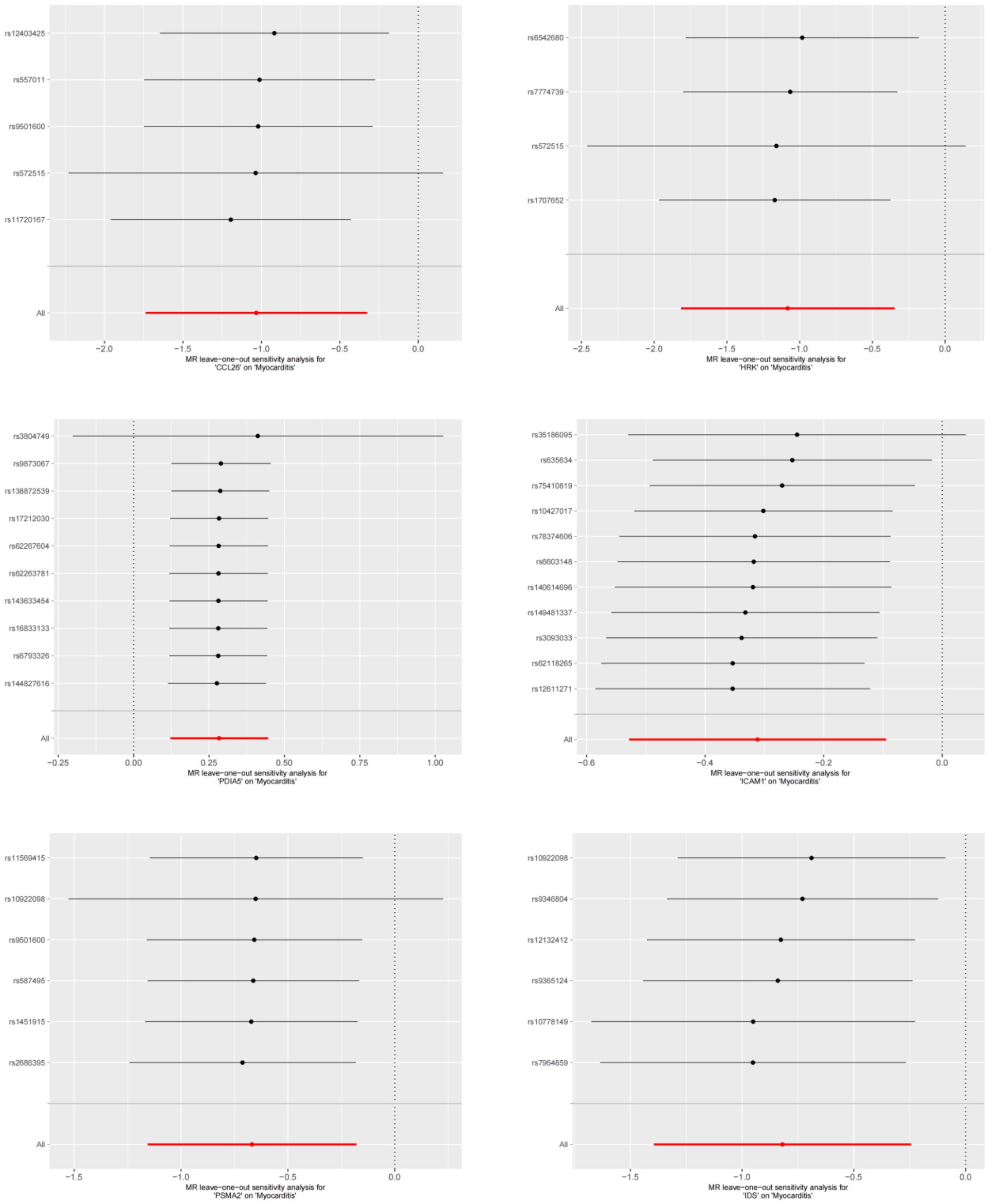

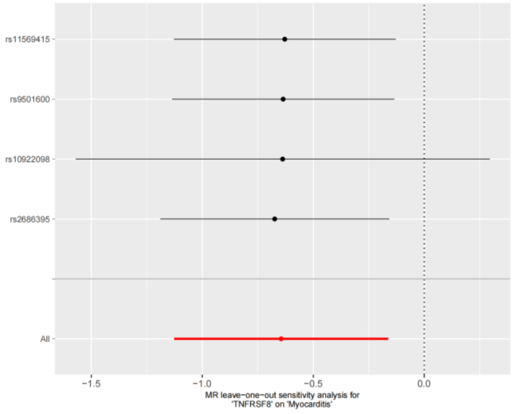
rs11569415
rs9501600
rs10922098
rs2686395
All
−1.5
−1.0
−0.5
0.0
MR leave−one−out sensitivity analysis for
'TNFRSF8' on 'Myocarditis'

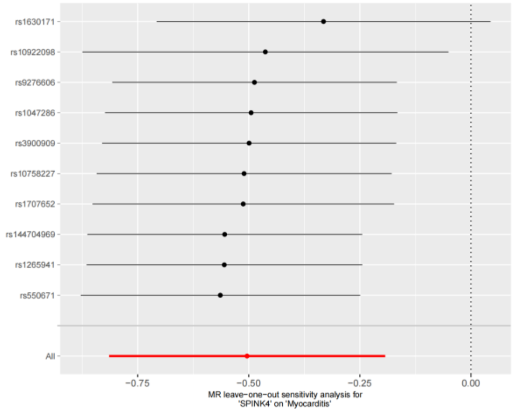
rs1630171
rs10922098
rs9276606
rs1047286
rs3900909
rs10758227
rs1707652
rs144704969
rs1265941
rs550671
All
−0.75
−0.50
−0.25
0.00
MR leave−one−out sensitivity analysis for
'SPINK4' on 'Myocarditis'

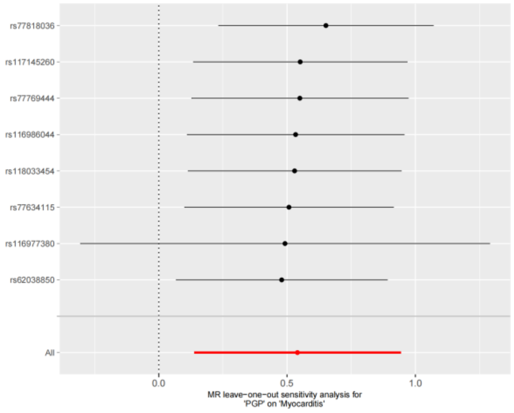
rs77818036
rs117145260
rs77769444
rs116986044
rs118033454
rs77634115
rs116977380
rs62038850
All
0.0
0.5
1.0
MR leave−one−out sensitivity analysis for
'PGP' on 'Myocarditis'

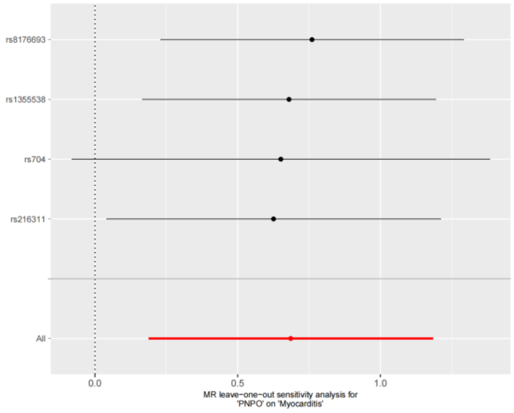
rs8176693
rs1355538
rs704
rs216311
All
0.0
0.5
1.0
MR leave−one−out sensitivity analysis for
'PNPO' on 'Myocarditis'

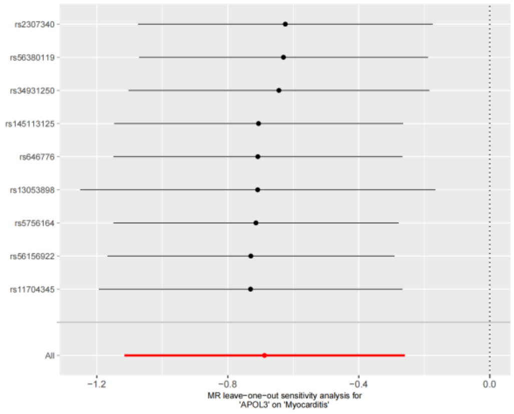
rs2307340
rs56380119
rs34931250
rs145113125
rs646776
rs13053898
rs5756164
rs56156922
rs11704345
All
−1.2
−0.8
−0.4
0.0
MR leave−one−out sensitivity analysis for
'APOL3' on 'Myocarditis'

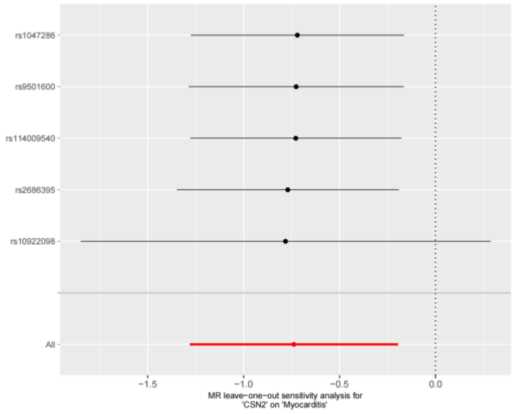
rs1047286
rs9501600
rs114009540
rs2686395
rs10922098
All
−1.5
−1.0
−0.5
0.0
MR leave−one−out sensitivity analysis for
'CSN2' on 'Myocarditis'

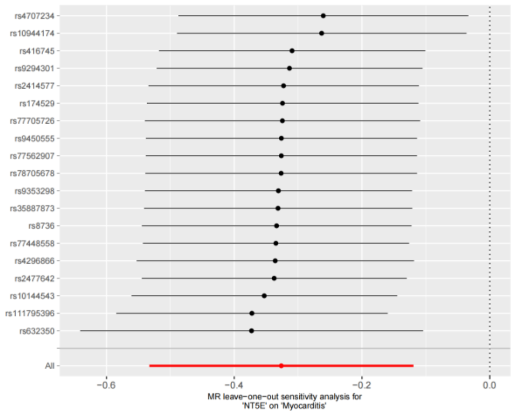
rs4707234
rs10944174
rs416745
rs9294301
rs2414577
rs174529
rs77705726
rs9450555
rs77562907
rs78705678
rs9353298
rs35887873
rs8736
rs77448558
rs4296866
rs2477642
rs10144543
rs111795396
rs632350
All
−0.6
−0.4
−0.2
0.0
MR leave−one−out sensitivity analysis for
'NT5E' on 'Myocarditis'

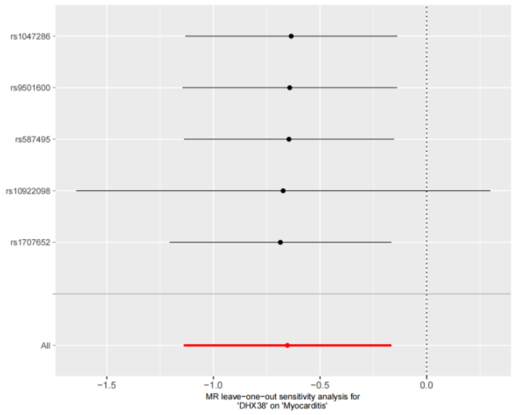
rs1047286
rs9501600
rs587495
rs10922098
rs1707652
All
−1.5
−1.0
−0.5
0.0
MR leave−one−out sensitivity analysis for
'DHX38' on 'Myocarditis'

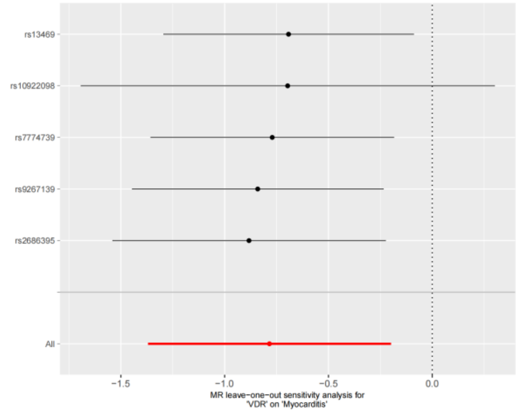

Figure S6. Leave-one-out sensitivity analysis with proteins mediating the mediating effect as exposure factors and myocarditis as outcome.

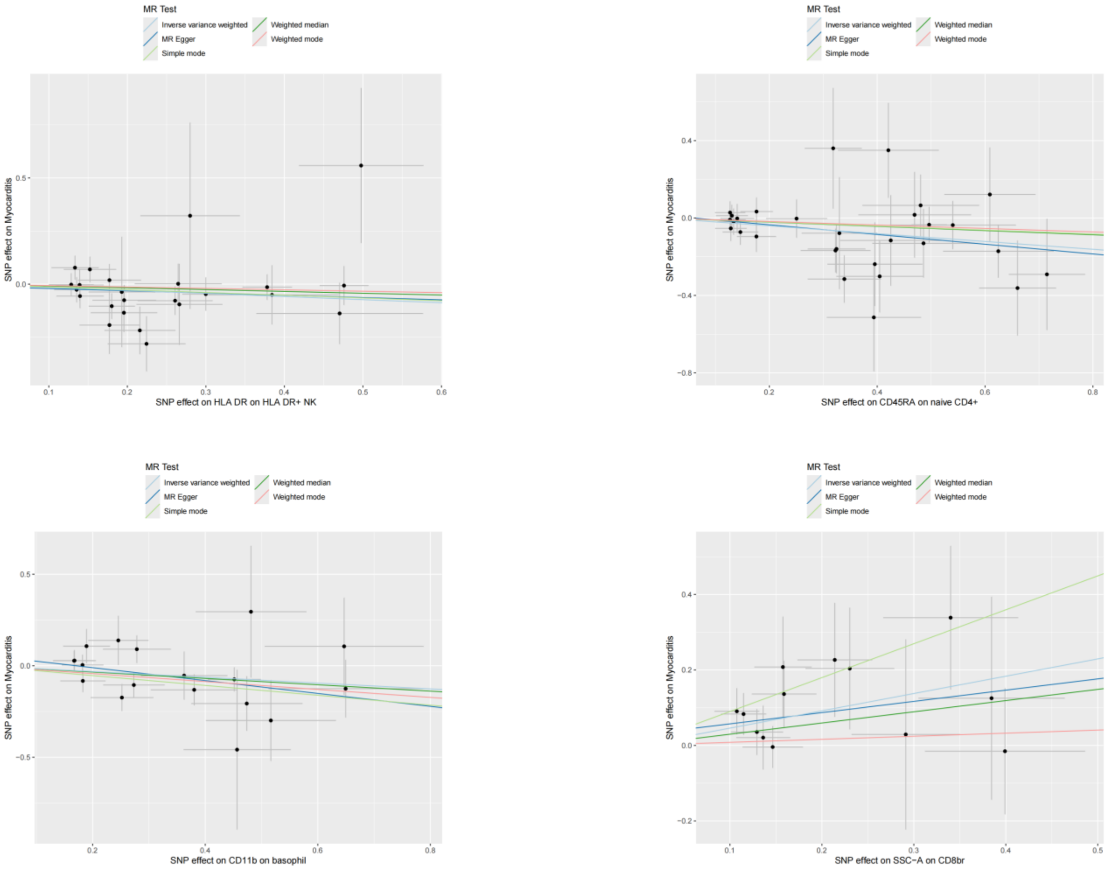

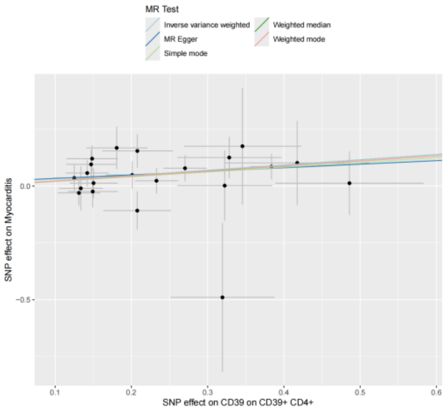
MR Test
Inverse variance weighted
MR Egger
Simple mode
Weighted median
Weighted mode
SNP effect on Myocarditis
SNP effect on CD39 on CD39+ CD4+

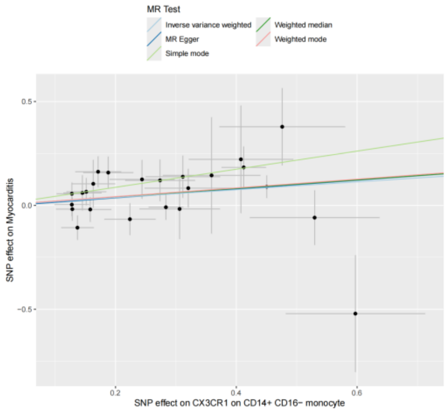
MR Test
Inverse variance weighted
MR Egger
Simple mode
Weighted median
Weighted mode
SNP effect on Myocarditis
SNP effect on CX3CR1 on CD14+ CD16− monocyte

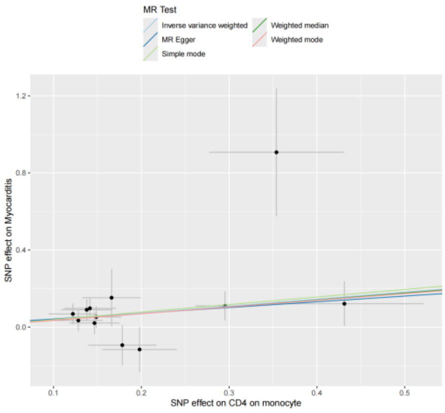
MR Test
Inverse variance weighted
MR Egger
Simple mode
Weighted median
Weighted mode
SNP effect on Myocarditis
SNP effect on CD4 on monocyte

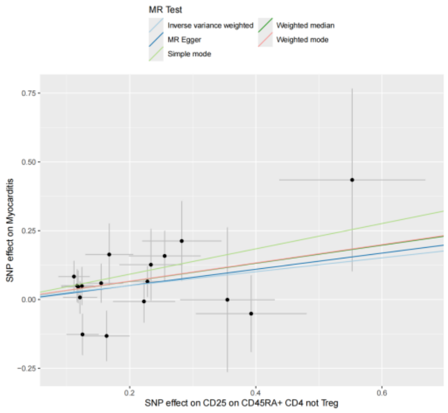
MR Test
Inverse variance weighted
MR Egger
Simple mode
Weighted median
Weighted mode
SNP effect on Myocarditis
SNP effect on CD25 on CD45RA+ CD4 not Treg

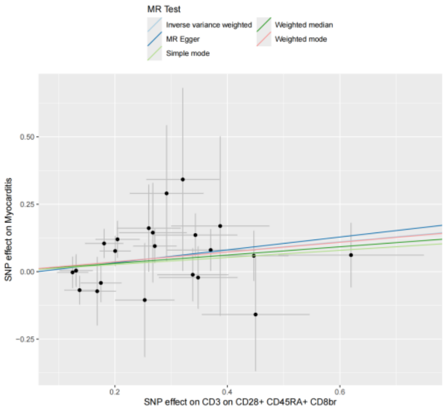
MR Test
Inverse variance weighted
MR Egger
Simple mode
Weighted median
Weighted mode
SNP effect on Myocarditis
SNP effect on CD3 on CD28+ CD45RA+ CD8br

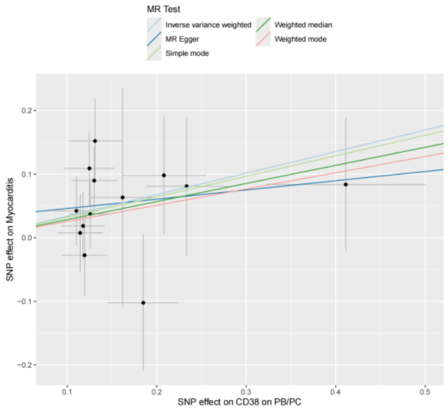
MR Test
Inverse variance weighted
MR Egger
Simple mode
Weighted median
Weighted mode
SNP effect on Myocarditis
SNP effect on CD38 on PB/PC

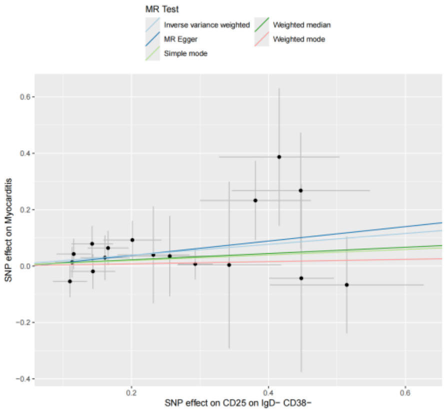
MR Test
Inverse variance weighted
MR Egger
Simple mode
Weighted median
Weighted mode
SNP effect on Myocarditis
SNP effect on CD25 on IgD− CD38−

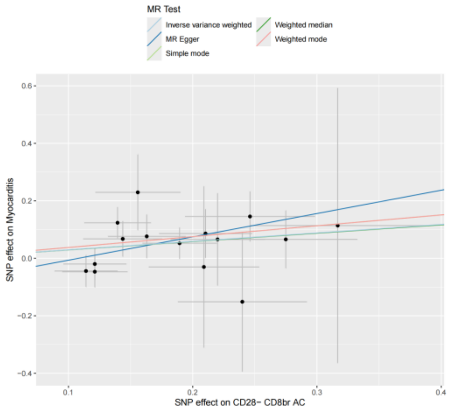
MR Test
Inverse variance weighted
MR Egger
Simple mode
Weighted median
Weighted mode
SNP effect on Myocarditis
SNP effect on CD28− CD8br AC

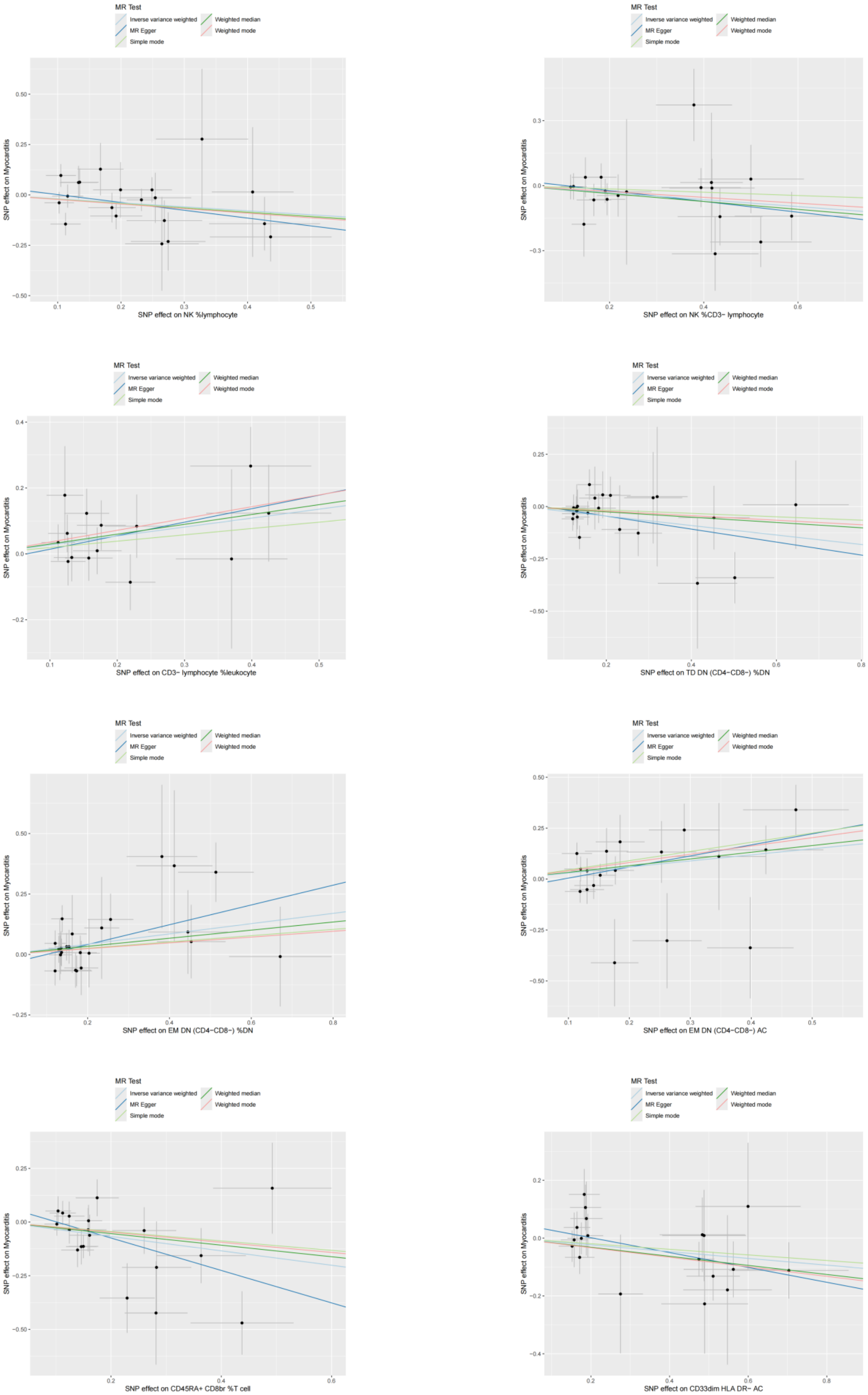
MR Test
Inverse variance weighted
MR Egger
Simple mode
Weighted median
Weighted mode
SNP effect on Myocarditis
SNP effect on NK %lymphocyte
SNP effect on NK %CD3− lymphocyte
SNP effect on CD3− lymphocyte %leukocyte
SNP effect on TD DN (CD4−CD8−) %DN
SNP effect on EM DN (CD4−CD8−) %DN
SNP effect on EM DN (CD4−CD8−) AC
SNP effect on CD45RA+ CD8br %T cell
SNP effect on CD33dim HLA DR− AC

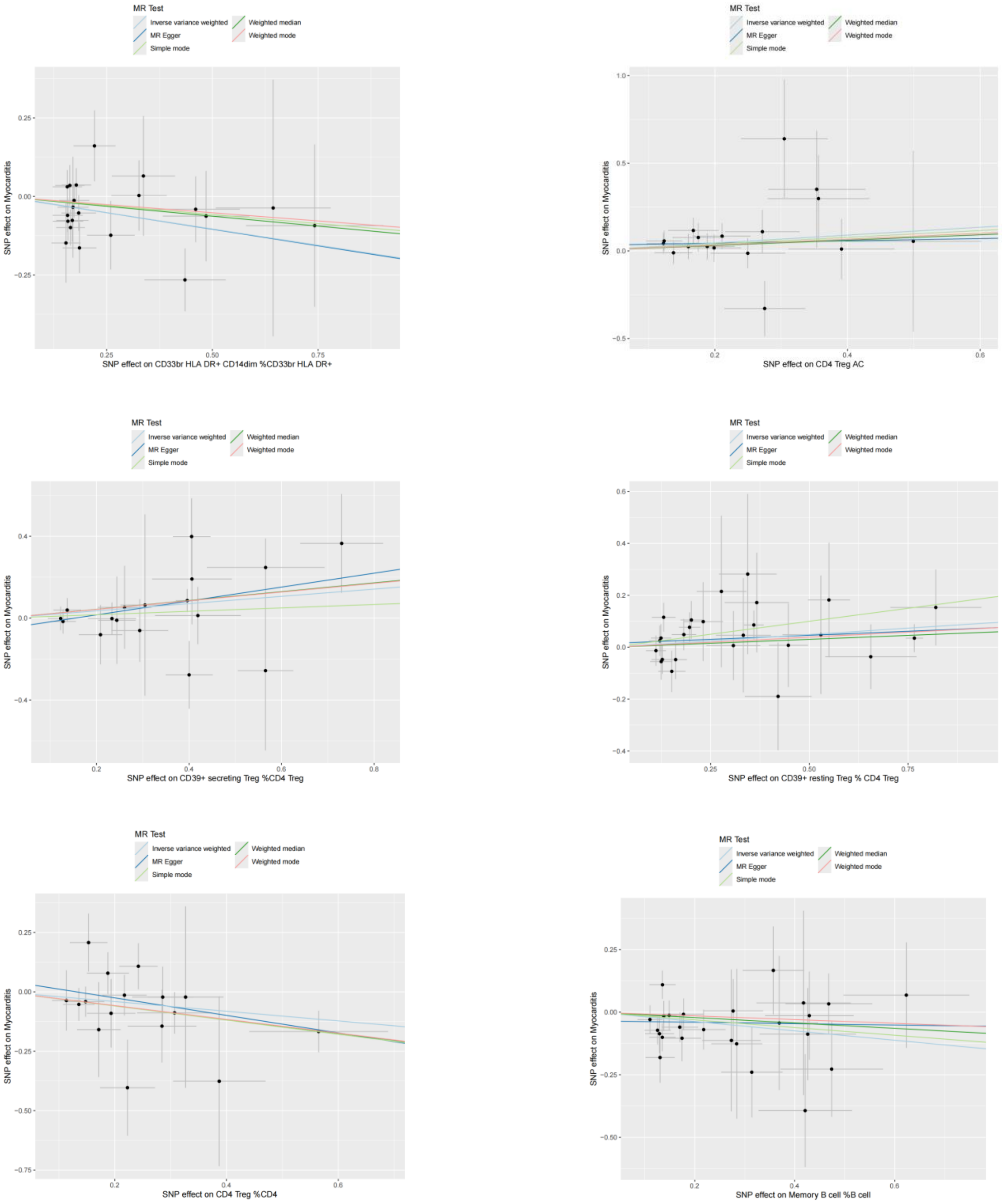

Figure S7. Scatter plots for immune cell as exposures and myocarditis as outcomes.

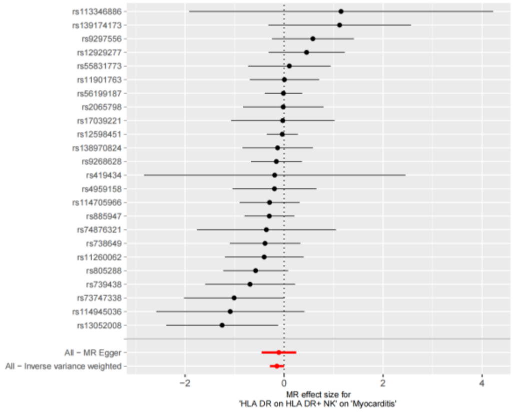
rs113346886
rs139174173
rs9297556
rs12929277
rs55831773
rs11901763
rs56199187
rs2065798
rs17039221
rs12598451
rs138970824
rs9268628
rs419434
rs4959158
rs114705966
rs885947
rs74876321
rs738649
rs11260062
rs805288
rs739438
rs73747338
rs114945036
rs13052008
All − MR Egger
All − Inverse variance weighted
MR effect size for
'HLA DR on HLA DR+ NK' on 'Myocarditis'

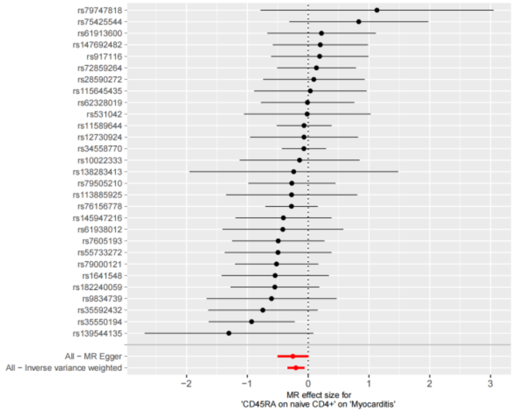
rs79747818
rs75425544
rs61913600
rs147692482
rs917116
rs72859264
rs28590272
rs115645435
rs62328019
rs531042
rs11589644
rs12730924
rs34558770
rs10022333
rs138283413
rs79505210
rs113885925
rs76156778
rs145947216
rs61938012
rs7605193
rs55733272
rs79000121
rs1641548
rs182240059
rs9834739
rs35592432
rs35550194
rs139544135
All − MR Egger
All − Inverse variance weighted
MR effect size for
'CD45RA on naive CD4+' on 'Myocarditis'

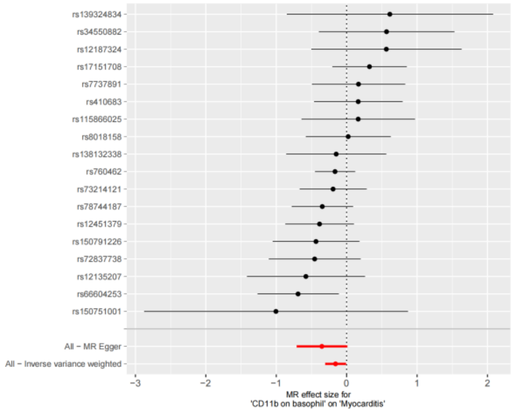
rs139324834
rs34550882
rs12187324
rs17151708
rs7737891
rs410683
rs115866025
rs8018158
rs138132338
rs760462
rs73214121
rs78744187
rs12451379
rs150791226
rs72837738
rs12135207
rs66604253
rs150751001
All − MR Egger
All − Inverse variance weighted
MR effect size for
'CD11b on basophil' on 'Myocarditis'

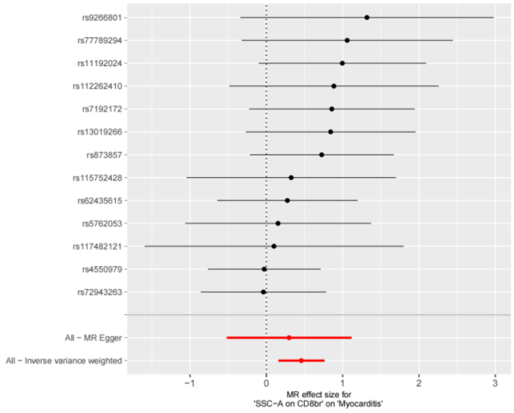
rs9266801
rs77789294
rs11192024
rs112262410
rs7192172
rs13019266
rs873857
rs115752428
rs62435615
rs5762053
rs117482121
rs4550979
rs72943263
All − MR Egger
All − Inverse variance weighted
MR effect size for
'SSC−A on CD8br' on 'Myocarditis'

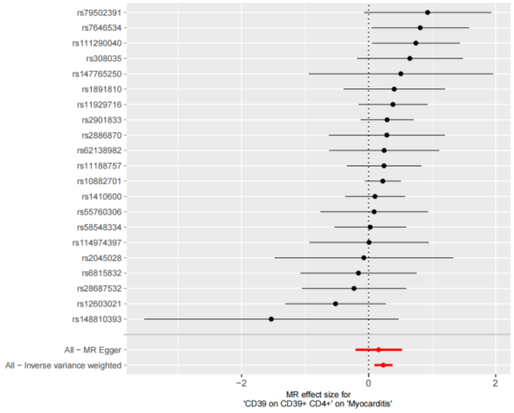
rs79502391
rs7646534
rs111290040
rs308035
rs147765250
rs1891810
rs11929716
rs2901833
rs2886870
rs62138982
rs11188757
rs10882701
rs1410600
rs55760306
rs58548334
rs114974397
rs2045028
rs6815832
rs28687532
rs12603021
rs148810393
All − MR Egger
All − Inverse variance weighted
MR effect size for
'CD39 on CD39+ CD4+' on 'Myocarditis'

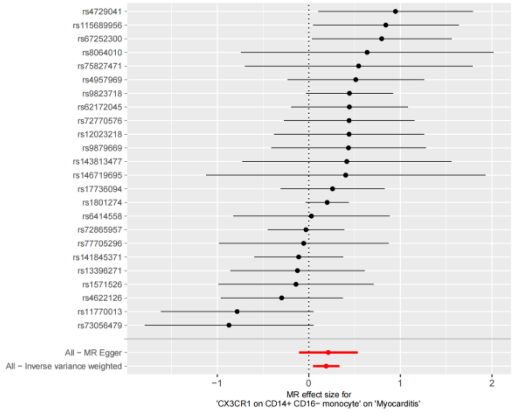
rs4729041
rs115689956
rs67252300
rs8064010
rs75827471
rs4957969
rs9823718
rs62172045
rs72770576
rs12023218
rs9879669
rs143813477
rs146719695
rs17736094
rs1801274
rs6414558
rs72865957
rs77705296
rs141845371
rs13396271
rs1571526
rs4622126
rs11770013
rs73056479
All − MR Egger
All − Inverse variance weighted
MR effect size for
'CX3CR1 on CD14+ CD16− monocyte' on 'Myocarditis'

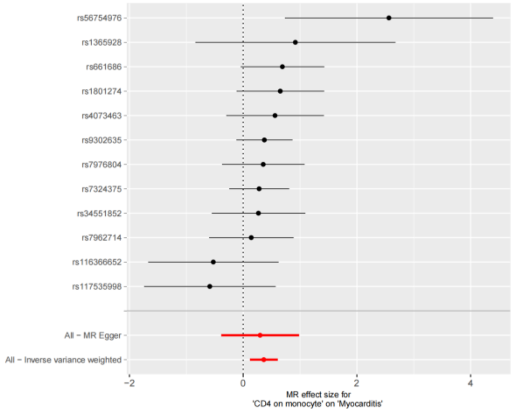
rs56754976
rs1365928
rs661686
rs1801274
rs4073463
rs9302635
rs7976804
rs7324375
rs34551852
rs7962714
rs116366652
rs117535998
All − MR Egger
All − Inverse variance weighted
MR effect size for
'CD4 on monocyte' on 'Myocarditis'

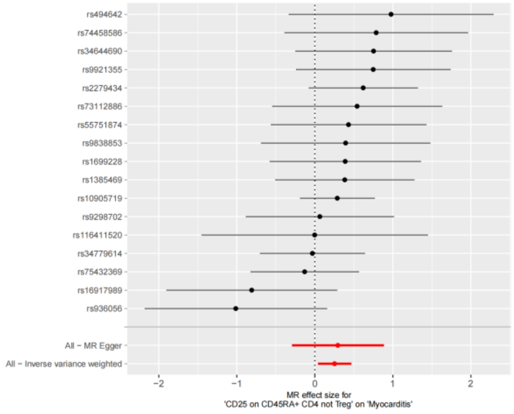
rs494642
rs74458586
rs34644690
rs9921355
rs2279434
rs73112886
rs55751874
rs9838853
rs1699228
rs1385469
rs10905719
rs9298702
rs116411520
rs34779614
rs75432369
rs16917989
rs936056
All − MR Egger
All − Inverse variance weighted
MR effect size for
'CD25 on CD45RA+ CD4 not Treg' on 'Myocarditis'

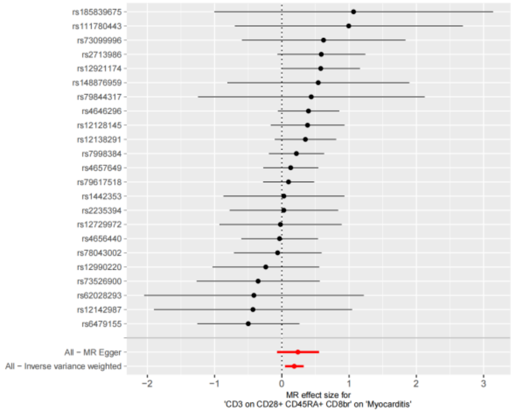
rs185839675
rs111780443
rs73099996
rs2713986
rs12921174
rs148876959
rs79844317
rs4646296
rs12128145
rs12138291
rs7998384
rs4657649
rs79617518
rs1442353
rs2235394
rs12729972
rs4656440
rs78043002
rs12990220
rs73526900
rs62028293
rs12142987
rs6479155
All − MR Egger
All − Inverse variance weighted
−2
−1
0
1
2
3
MR effect size for
'CD3 on CD28+ CD45RA+ CD8br' on 'Myocarditis'

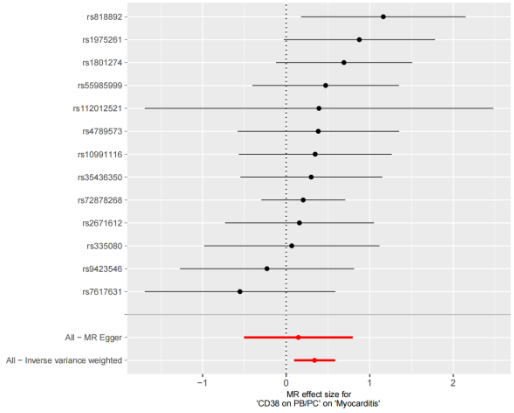
rs818892
rs1975261
rs1801274
rs55985999
rs112012521
rs4789573
rs10991116
rs35436350
rs72878268
rs2671612
rs335080
rs9423546
rs7617631
All − MR Egger
All − Inverse variance weighted
−1
0
1
2
MR effect size for
'CD38 on PB/PC' on 'Myocarditis'

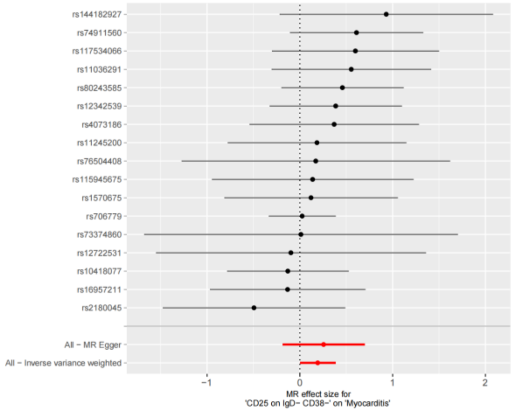
rs144182927
rs74911560
rs117534066
rs11036291
rs80243585
rs12342539
rs4073186
rs11245200
rs76504408
rs115945675
rs1570675
rs706779
rs73374860
rs12722531
rs10418077
rs16957211
rs2180045
All − MR Egger
All − Inverse variance weighted
−1
0
1
2
MR effect size for
'CD25 on IgD− CD38−' on 'Myocarditis'

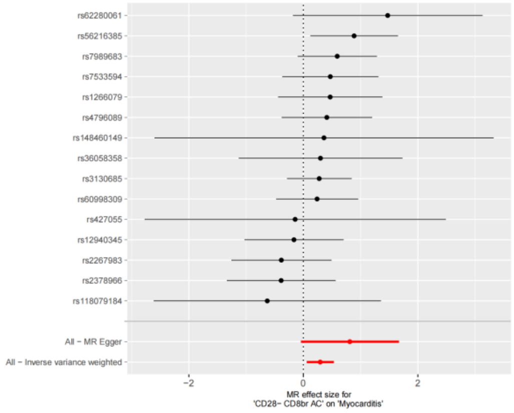
rs62280061
rs56216385
rs7989683
rs7533594
rs1266079
rs4796089
rs148460149
rs36058358
rs3130685
rs60998309
rs427055
rs12940345
rs2267983
rs2378966
rs118079184
All − MR Egger
All − Inverse variance weighted
−2
0
2
MR effect size for
'CD28− CD8br AC' on 'Myocarditis'

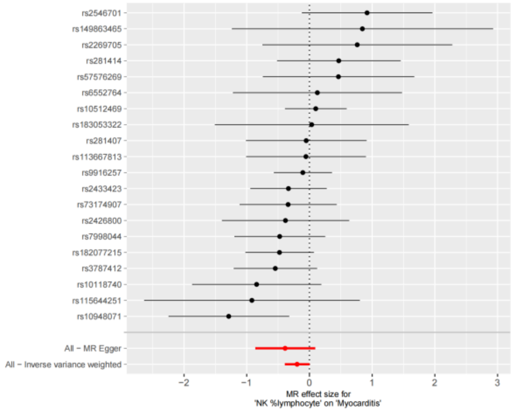
rs2546701
rs149863465
rs2269705
rs281414
rs57576269
rs6552764
rs10512469
rs183053322
rs281407
rs113667813
rs9916257
rs2433423
rs73174907
rs2426800
rs7998044
rs182077215
rs3787412
rs10118740
rs115644251
rs10948071
All − MR Egger
All − Inverse variance weighted
−2
−1
0
1
2
3
MR effect size for
'NK %lymphocyte' on 'Myocarditis'

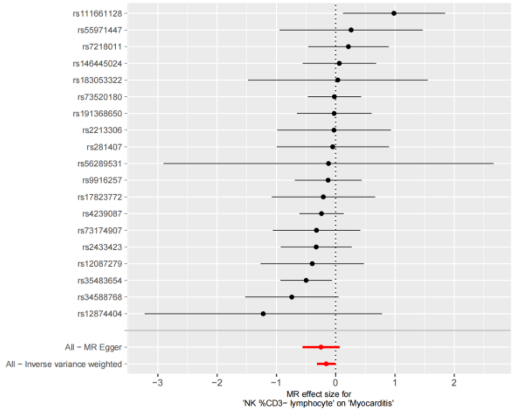
rs111661128
rs55971447
rs7218011
rs146445024
rs183053322
rs73520180
rs191368650
rs2213306
rs281407
rs56289531
rs9916257
rs17823772
rs4239087
rs73174907
rs2433423
rs12087279
rs35483654
rs34588768
rs12874404
All − MR Egger
All − Inverse variance weighted
−3
−2
−1
0
1
2
MR effect size for
'NK %CD3− lymphocyte' on 'Myocarditis'

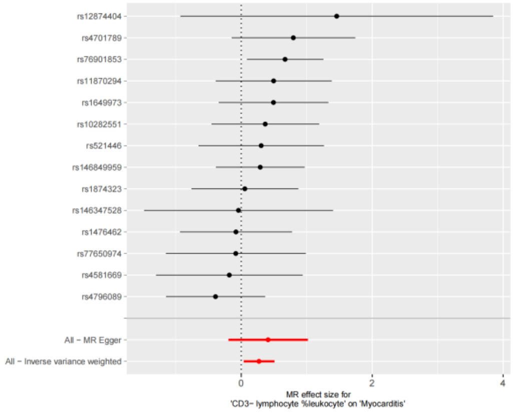
rs12874404
rs4701789
rs76901853
rs11870294
rs1649973
rs10282551
rs521446
rs146849959
rs1874323
rs146347528
rs1476462
rs77650974
rs4581669
rs4796089
All − MR Egger
All − Inverse variance weighted
0
2
4
MR effect size for
'CD3− lymphocyte %leukocyte' on 'Myocarditis'

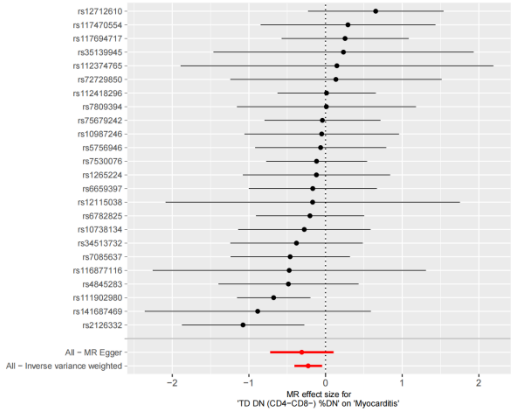
rs12712610
rs117470554
rs117694717
rs35139945
rs112374765
rs72729850
rs112418296
rs7809394
rs75679242
rs10987246
rs5756946
rs7530076
rs1265224
rs6659397
rs12115038
rs6782825
rs10738134
rs34513732
rs7085637
rs116877116
rs4845283
rs111902980
rs141687469
rs2126332
All − MR Egger
All − Inverse variance weighted
−2
−1
0
1
2
MR effect size for
'TD DN (CD4−CD8−) %DN' on 'Myocarditis'

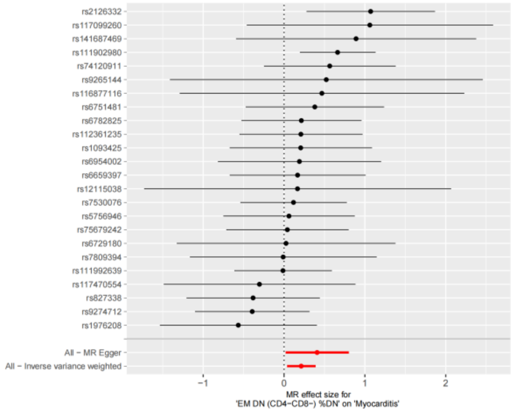
rs2126332
rs117099260
rs141687469
rs111902980
rs74120911
rs9265144
rs116877116
rs6751481
rs6782825
rs112361235
rs1093425
rs6954002
rs6659397
rs12115038
rs7530076
rs5756946
rs75679242
rs6729180
rs7809394
rs111992639
rs117470554
rs827338
rs9274712
rs1976208
All − MR Egger
All − Inverse variance weighted
−1
0
1
2
MR effect size for
'EM DN (CD4−CD8−) %DN' on 'Myocarditis'

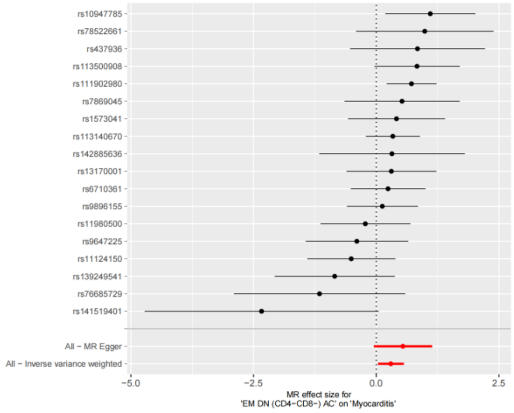
rs10947785
rs78522661
rs437936
rs113500908
rs111902980
rs7869045
rs1573041
rs113140670
rs142885636
rs13170001
rs6710361
rs9896155
rs11980500
rs9647225
rs11124150
rs139249541
rs76685729
rs141519401
All − MR Egger
All − Inverse variance weighted
−5.0
−2.5
0.0
2.5
MR effect size for
'EM DN (CD4−CD8−) AC' on 'Myocarditis'

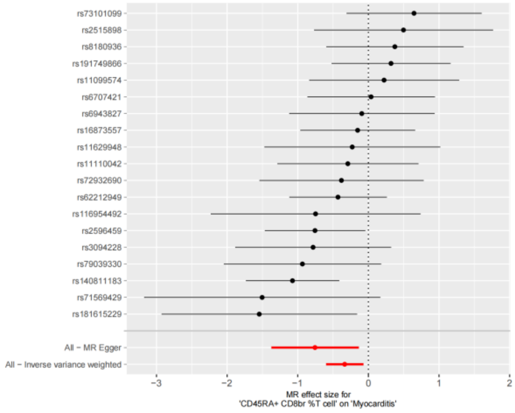
rs73101099
rs2515898
rs8180936
rs191749866
rs11099574
rs6707421
rs6943827
rs16873557
rs11629948
rs11110042
rs72932690
rs62212949
rs116954492
rs2596459
rs3094228
rs79039330
rs140811183
rs71569429
rs181615229
All − MR Egger
All − Inverse variance weighted
−3
−2
−1
0
1
2
MR effect size for
'CD45RA+ CD8br %T cell' on 'Myocarditis'

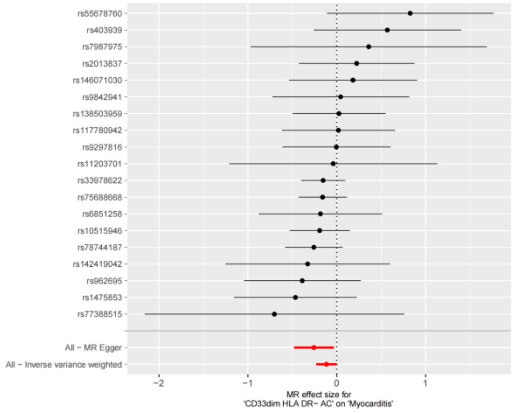
rs55678760
rs403939
rs7987975
rs2013837
rs146071030
rs9842941
rs138503959
rs117780942
rs9297816
rs11203701
rs33978622
rs75688668
rs6851258
rs10515946
rs78744187
rs142419042
rs962695
rs1475853
rs77388515
All − MR Egger
All − Inverse variance weighted
−2
−1
0
1
MR effect size for
'CD33dim HLA DR− AC' on 'Myocarditis'

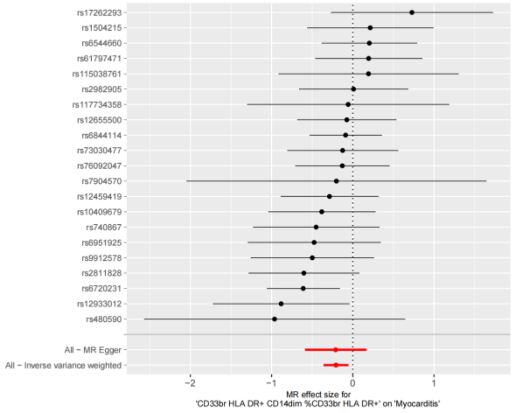
rs17262293
rs1504215
rs6544660
rs61797471
rs115038761
rs2982905
rs117734358
rs12655500
rs6844114
rs73030477
rs76092047
rs7904570
rs12459419
rs10409679
rs740867
rs6951925
rs9912578
rs2811828
rs6720231
rs12933012
rs480590
All − MR Egger
All − Inverse variance weighted
−2
−1
0
1
MR effect size for
'CD33br HLA DR+ CD14dim %CD33br HLA DR+' on 'Myocarditis'

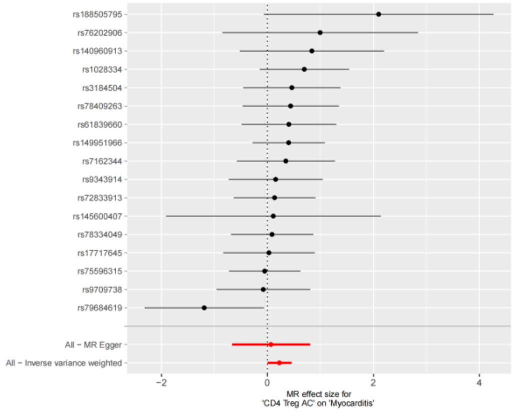
rs188505795
rs76202906
rs140960913
rs1028334
rs3184504
rs78409263
rs61839660
rs149951966
rs7162344
rs9343914
rs72833913
rs145600407
rs78334049
rs17717645
rs75596315
rs9709738
rs79684619
All − MR Egger
All − Inverse variance weighted
−2
0
2
4
MR effect size for
'CD4 Treg AC' on 'Myocarditis'

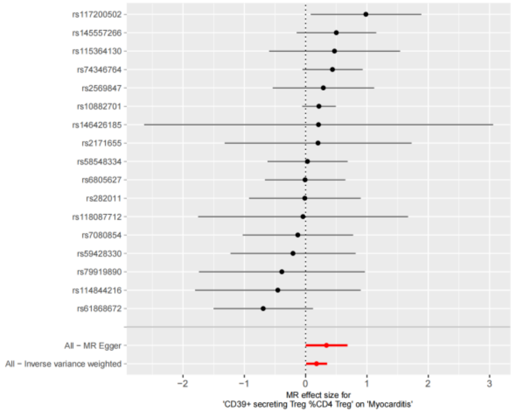
rs117200502
rs145557266
rs115364130
rs74346764
rs2569847
rs10882701
rs146426185
rs2171655
rs58548334
rs6805627
rs282011
rs118087712
rs7080854
rs59428330
rs79919890
rs114844216
rs61868672
All − MR Egger
All − Inverse variance weighted
−2
−1
0
1
2
3
MR effect size for
'CD39+ secreting Treg %CD4 Treg' on 'Myocarditis'

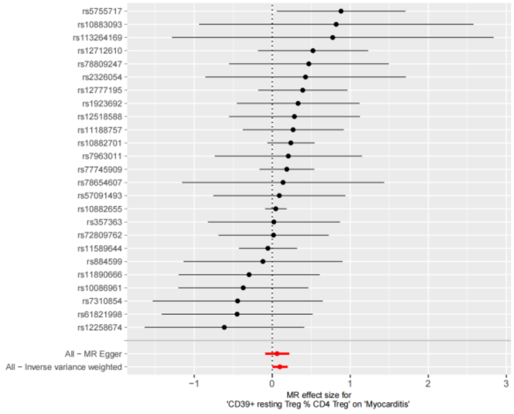
rs5755717
rs10883093
rs113264169
rs12712610
rs78809247
rs2326054
rs12777195
rs1923692
rs12518588
rs11188757
rs10882701
rs7963011
rs77745909
rs78654607
rs57091493
rs10882655
rs357363
rs72809762
rs11589644
rs884599
rs11890666
rs10086961
rs7310854
rs61821998
rs12258674
All − MR Egger
All − Inverse variance weighted
−1
0
1
2
3
MR effect size for
'CD39+ resting Treg % CD4 Treg' on 'Myocarditis'

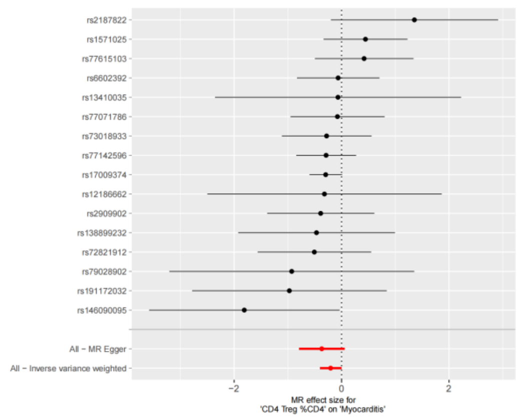

Figure S8. Forest plots for immune cell as exposures and myocarditis as outcomes.

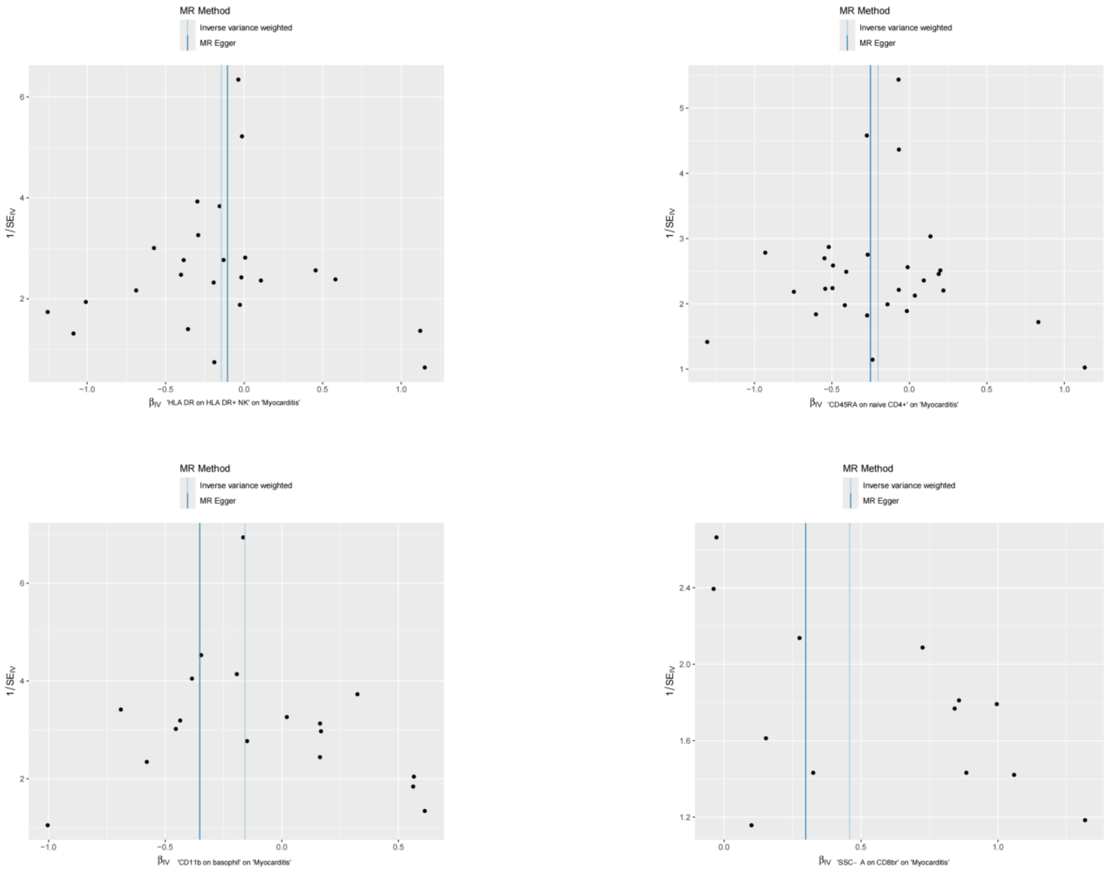

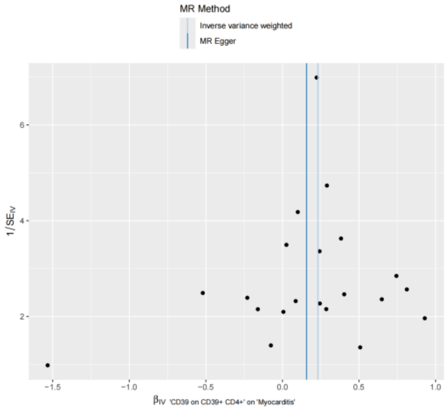
MR Method
Inverse variance weighted
MR Egger
1/SE_IV
βIV 'CD39 on CD39+ CD4+' on 'Myocarditis'

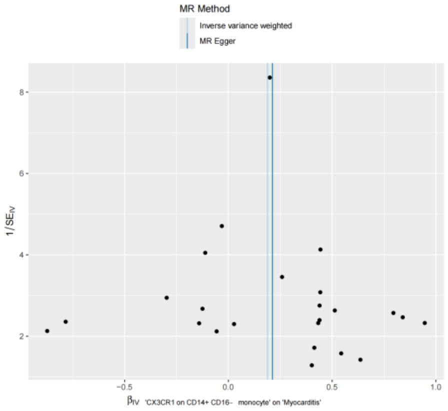
MR Method
Inverse variance weighted
MR Egger
1/SE_IV
βIV 'CX3CR1 on CD14+ CD16− monocyte' on 'Myocarditis'

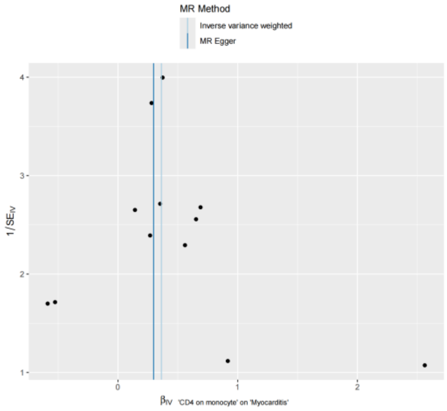
MR Method
Inverse variance weighted
MR Egger
1/SE_IV
βIV 'CD4 on monocyte' on 'Myocarditis'

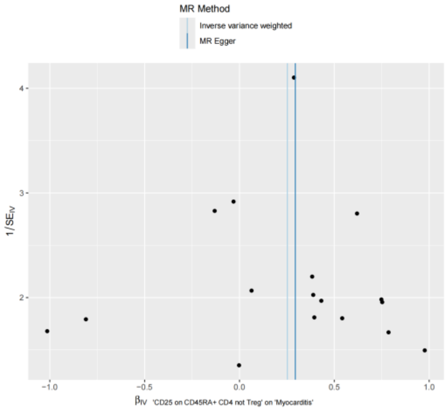
MR Method
Inverse variance weighted
MR Egger
1/SE_IV
βIV 'CD25 on CD45RA+ CD4 not Treg' on 'Myocarditis'

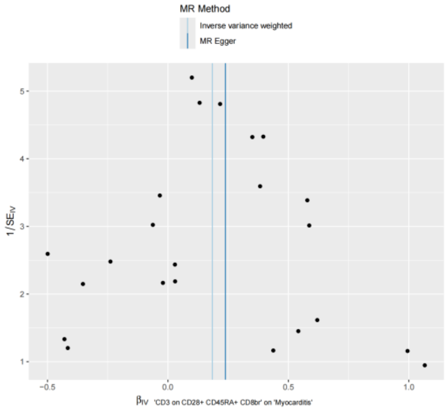
MR Method
Inverse variance weighted
MR Egger
1/SE_IV
βIV 'CD3 on CD28+ CD45RA+ CD8br' on 'Myocarditis'

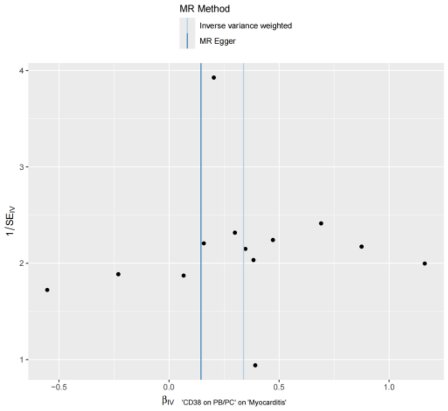
MR Method
Inverse variance weighted
MR Egger
1/SE_IV
βIV 'CD38 on PB/PC' on 'Myocarditis'

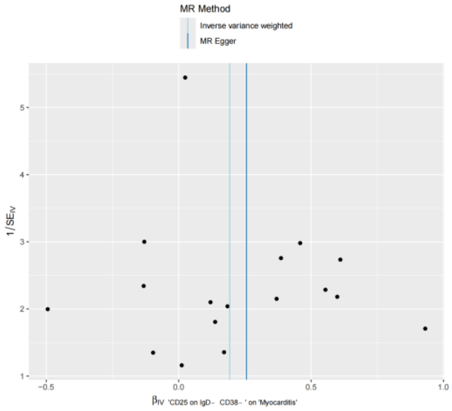
MR Method
Inverse variance weighted
MR Egger
1/SE_IV
βIV 'CD25 on IgD− CD38− ' on 'Myocarditis'

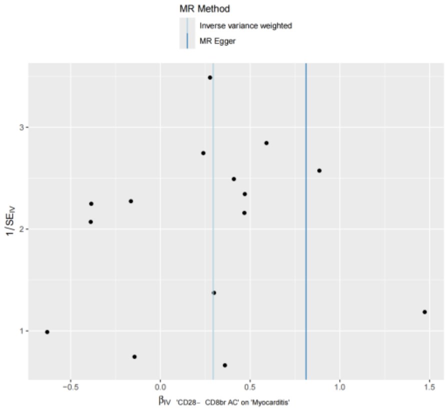
MR Method
Inverse variance weighted
MR Egger
1/SE_IV
βIV 'CD28− CD8br AC' on 'Myocarditis'

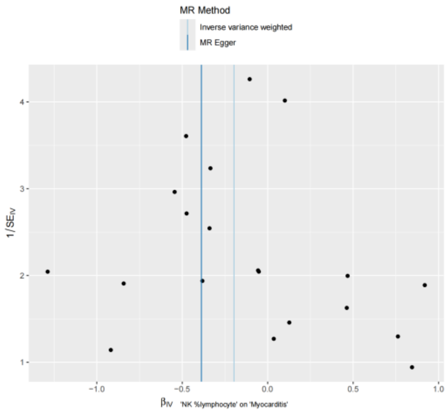
MR Method
Inverse variance weighted
MR Egger
1/SE_IV
β_IV 'NK %lymphocyte' on 'Myocarditis'

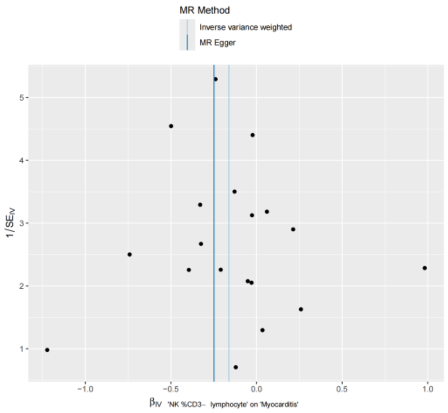
MR Method
Inverse variance weighted
MR Egger
1/SE_IV
β_IV 'NK %CD3- lymphocyte' on 'Myocarditis'

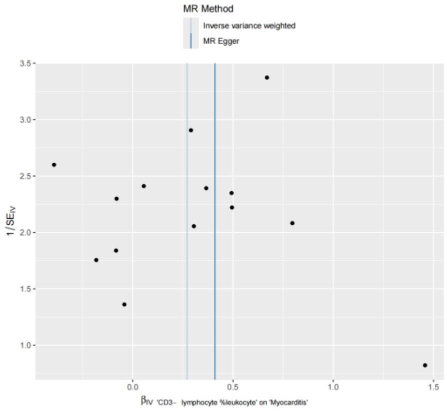
MR Method
Inverse variance weighted
MR Egger
1/SE_IV
β_IV 'CD3- lymphocyte %leukocyte' on 'Myocarditis'

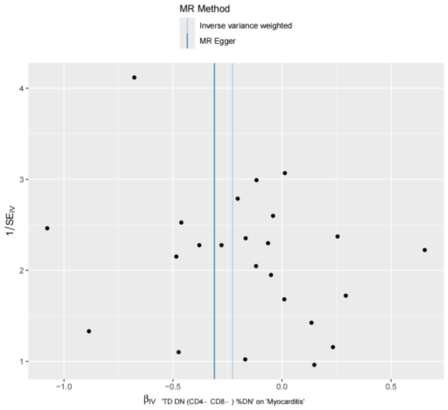
MR Method
Inverse variance weighted
MR Egger
1/SE_IV
β_IV 'TD DN (CD4- CD8-) %DN' on 'Myocarditis'

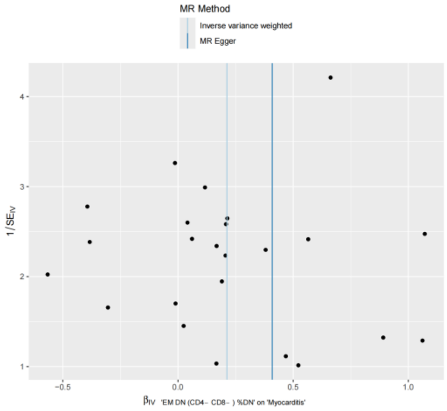
MR Method
Inverse variance weighted
MR Egger
1/SE_IV
β_IV 'EM DN (CD4- CD8-) %DN' on 'Myocarditis'

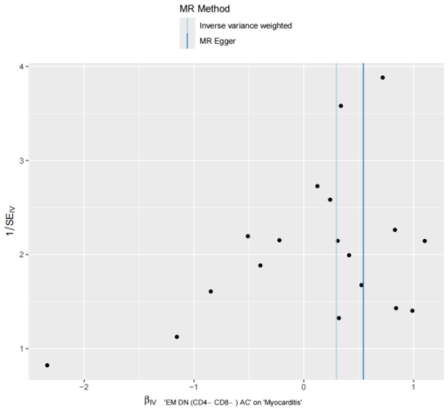
MR Method
Inverse variance weighted
MR Egger
1/SE_IV
β_IV 'EM DN (CD4- CD8-) AC' on 'Myocarditis'

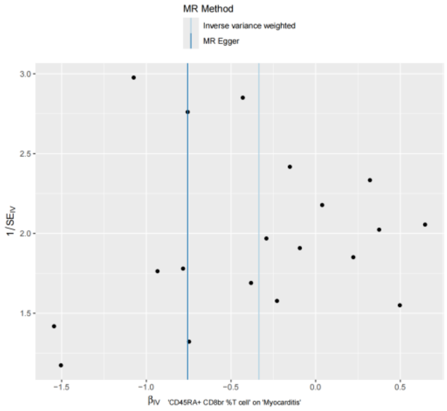
MR Method
Inverse variance weighted
MR Egger
1/SE_IV
β_IV 'CD45RA+ CD8br %T cell' on 'Myocarditis'

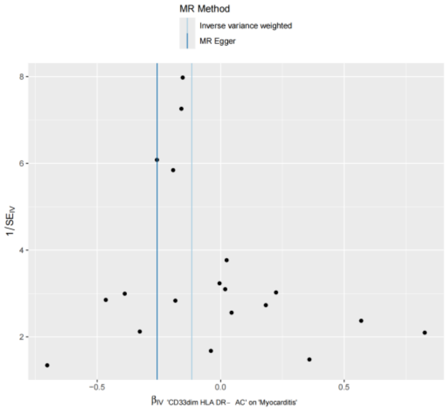
MR Method
Inverse variance weighted
MR Egger
1/SE_IV
β_IV 'CD33dim HLA DR- AC' on 'Myocarditis'

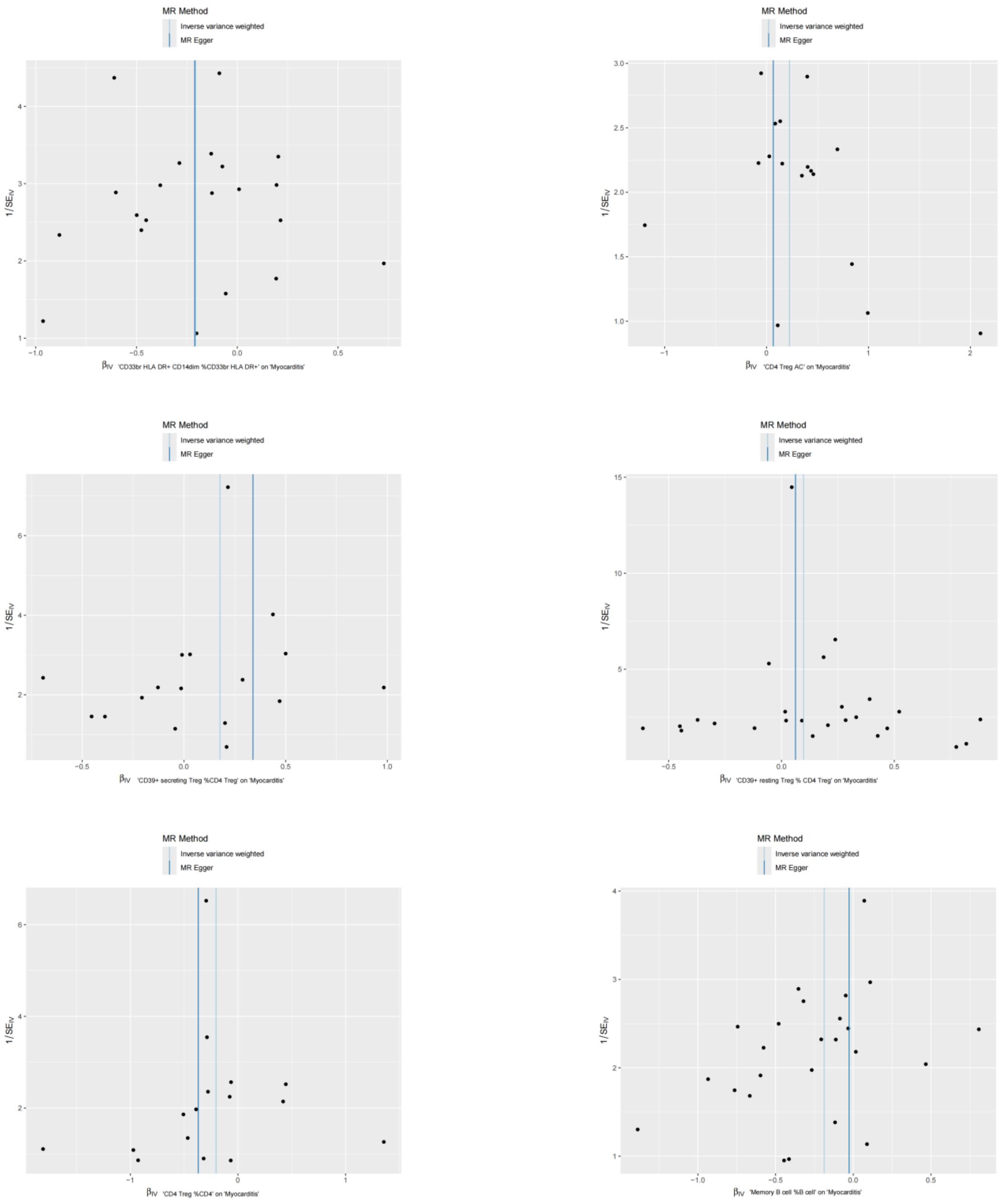

Figure S9. Funnel plots for immune cell as exposures and myocarditis as outcomes.

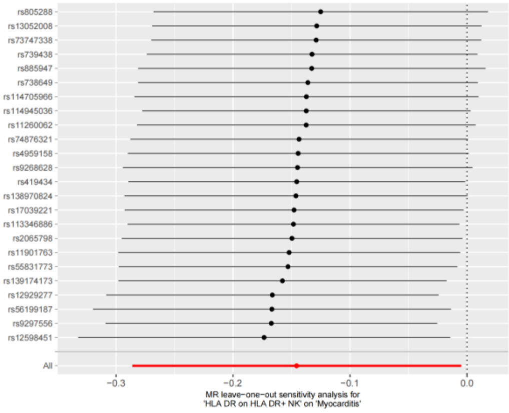
MR leave−one−out sensitivity analysis for
'HLA DR on HLA DR+ NK' on 'Myocarditis'

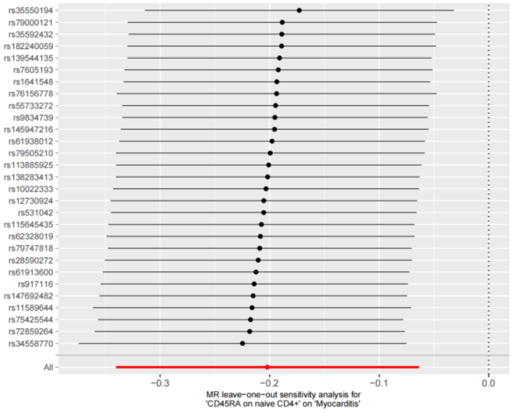
MR leave−one−out sensitivity analysis for
'CD45RA on naive CD4+' on 'Myocarditis'

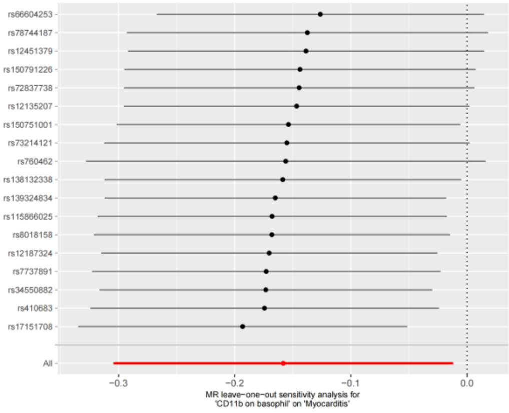
MR leave−one−out sensitivity analysis for
'CD11b on basophil' on 'Myocarditis'

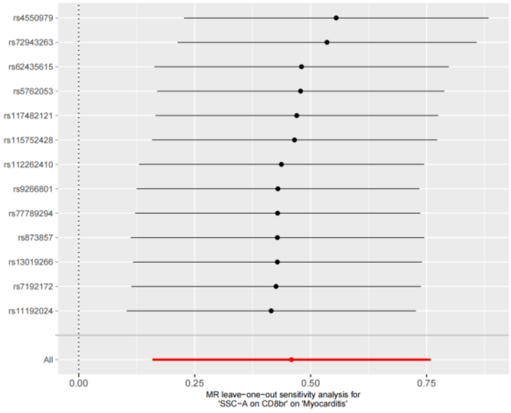
MR leave−one−out sensitivity analysis for
'SSC-A on CD8br' on 'Myocarditis'

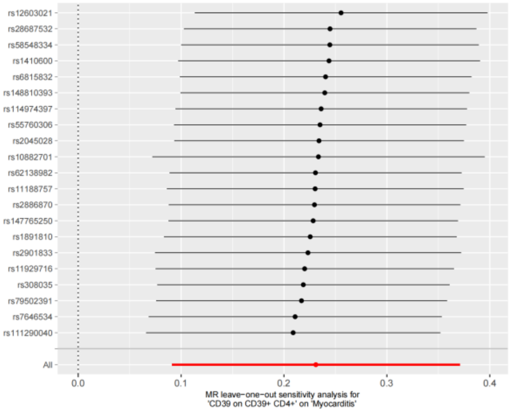
MR leave−one−out sensitivity analysis for
'CD39 on CD39+ CD4+' on 'Myocarditis'

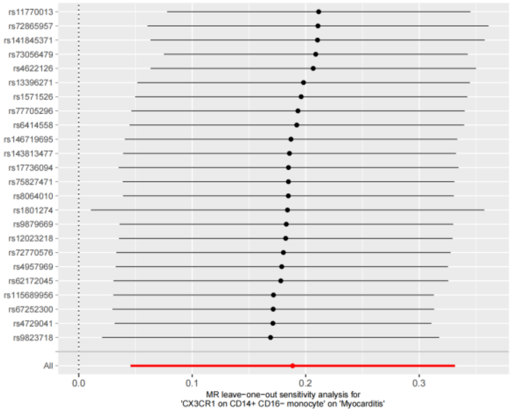
MR leave−one−out sensitivity analysis for
'CX3CR1 on CD14+ CD16− monocyte' on 'Myocarditis'

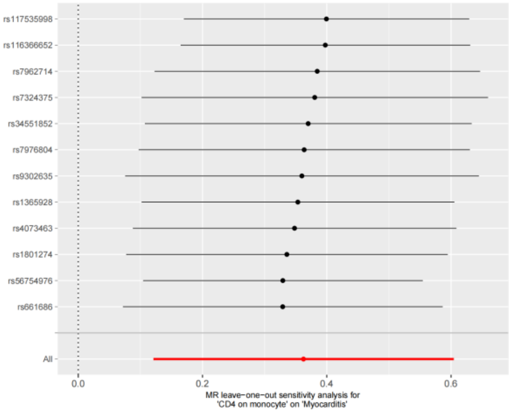
MR leave−one−out sensitivity analysis for
'CD4 on monocyte' on 'Myocarditis'

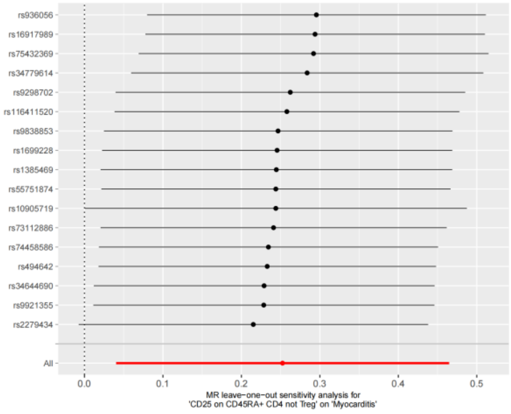
MR leave−one−out sensitivity analysis for
'CD25 on CD45RA+ CD4 not Treg' on 'Myocarditis'

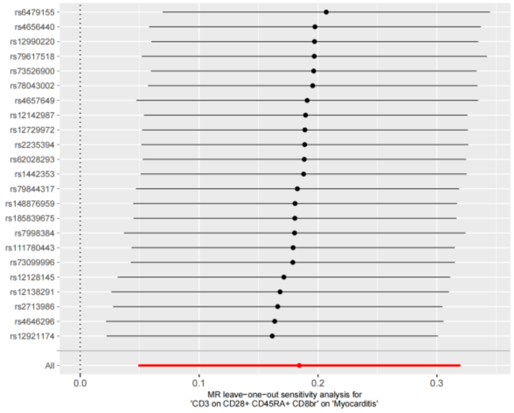
rs6479155
rs4656440
rs12990220
rs79617518
rs73526900
rs78043002
rs4657649
rs12142987
rs12729972
rs2235394
rs62028293
rs1442353
rs79844317
rs148876959
rs185839675
rs7998384
rs111780443
rs73099996
rs12128145
rs12138291
rs2713986
rs4646296
rs12921174
All
0.0
0.1
0.2
0.3
MR leave−one−out sensitivity analysis for
'CD3 on CD28+ CD45RA+ CD8br' on 'Myocarditis'

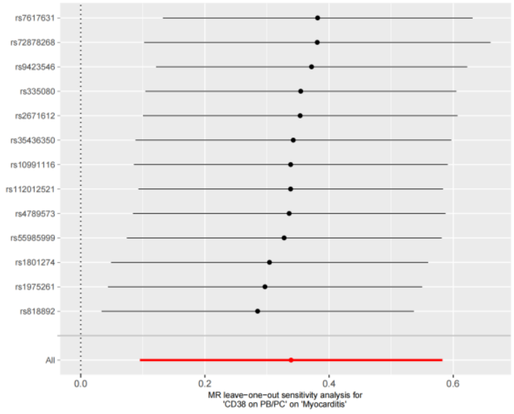
rs7617631
rs72878268
rs9423546
rs335080
rs2671612
rs35436350
rs10991116
rs112012521
rs4789573
rs55985999
rs1801274
rs1975261
rs818892
All
0.0
0.2
0.4
0.6
MR leave−one−out sensitivity analysis for
'CD38 on PB/PC' on 'Myocarditis'

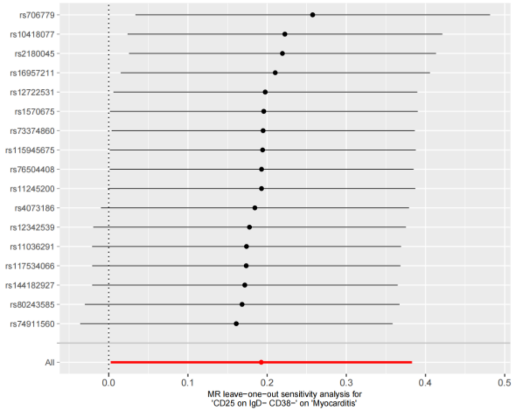
rs706779
rs10418077
rs2180045
rs16957211
rs12722531
rs1570675
rs73374860
rs115945675
rs76504408
rs11245200
rs4073186
rs12342539
rs11036291
rs117534066
rs144182927
rs80243585
rs74911560
All
0.0
0.1
0.2
0.3
0.4
0.5
MR leave−one−out sensitivity analysis for
'CD25 on IgD− CD38−' on 'Myocarditis'

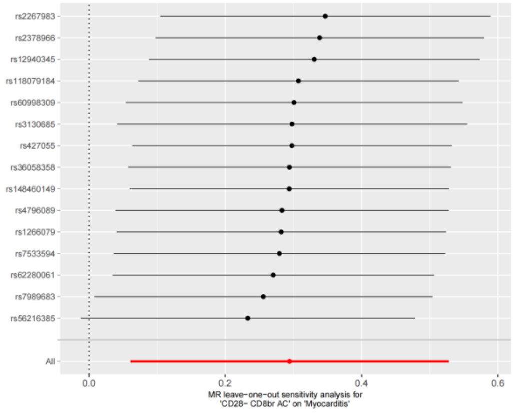
rs2267983
rs2378966
rs12940345
rs118079184
rs60998309
rs3130685
rs427055
rs36058358
rs148460149
rs4796089
rs1266079
rs7533594
rs62280061
rs7989683
rs56216385
All
0.0
0.2
0.4
0.6
MR leave−one−out sensitivity analysis for
'CD28− CD8br AC' on 'Myocarditis'

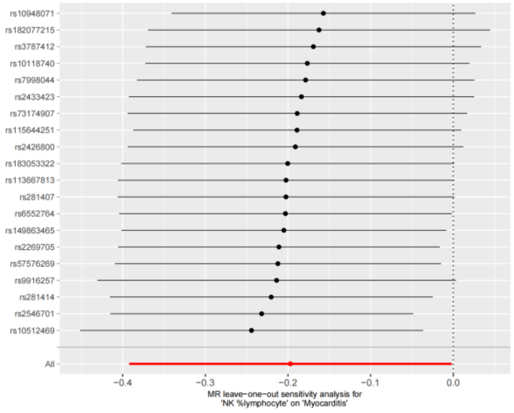
rs10948071
rs182077215
rs3787412
rs10118740
rs7998044
rs2433423
rs73174907
rs115644251
rs2426800
rs183053322
rs113667813
rs281407
rs6552764
rs149863465
rs2269705
rs57576269
rs9916257
rs281414
rs2546701
rs10512469
All
−0.4
−0.3
−0.2
−0.1
0.0
MR leave−one−out sensitivity analysis for
'NK %lymphocyte' on 'Myocarditis'

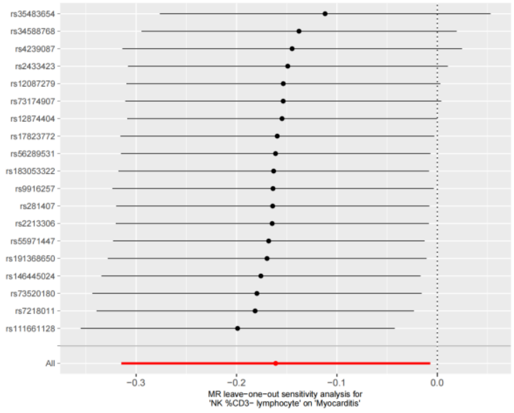
rs35483654
rs34588768
rs4239087
rs2433423
rs12087279
rs73174907
rs12874404
rs17823772
rs56289531
rs183053322
rs9916257
rs281407
rs2213306
rs55971447
rs191368650
rs146445024
rs73520180
rs7218011
rs111661128
All
−0.3
−0.2
−0.1
0.0
MR leave−one−out sensitivity analysis for
'NK %CD3− lymphocyte' on 'Myocarditis'

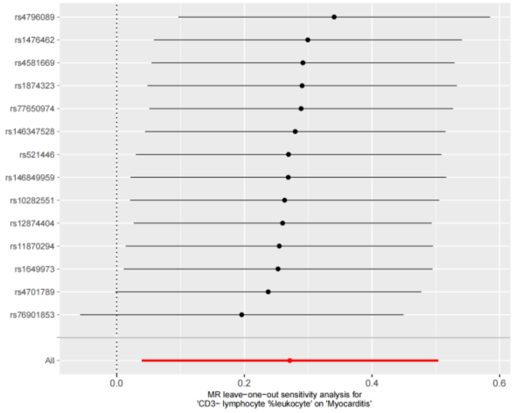
rs4796089
rs1476462
rs4581669
rs1874323
rs77650974
rs146347528
rs521446
rs146849959
rs10282551
rs12874404
rs11870294
rs1649973
rs4701789
rs76901853
All
0.0
0.2
0.4
0.6
MR leave−one−out sensitivity analysis for
'CD3− lymphocyte %leukocyte' on 'Myocarditis'

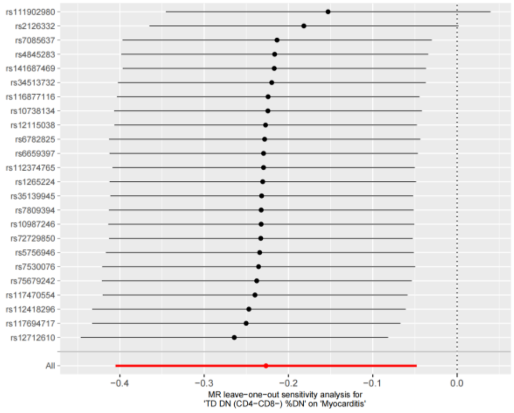
rs111902980
rs2126332
rs7085637
rs4845283
rs141687469
rs34513732
rs116877116
rs10738134
rs12115038
rs6782825
rs6659397
rs112374765
rs1265224
rs35139945
rs7809394
rs10987246
rs72729850
rs5756946
rs7530076
rs75679242
rs117470554
rs112418296
rs117694717
rs12712610
All
−0.4
−0.3
−0.2
−0.1
0.0
MR leave−one−out sensitivity analysis for
'TD DN (CD4−CD8−) %DN' on 'Myocarditis'

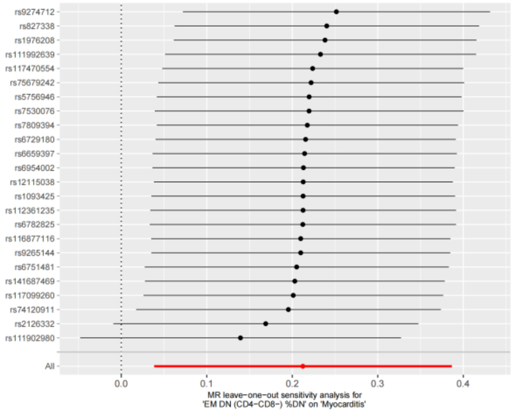
rs9274712
rs827338
rs1976208
rs111992639
rs117470554
rs75679242
rs5756946
rs7530076
rs7809394
rs6729180
rs6659397
rs6954002
rs12115038
rs1093425
rs112361235
rs6782825
rs116877116
rs9265144
rs6751481
rs141687469
rs117099260
rs74120911
rs2126332
rs111902980
All
0.0
0.1
0.2
0.3
0.4
MR leave−one−out sensitivity analysis for
'EM DN (CD4−CD8−) %DN' on 'Myocarditis'

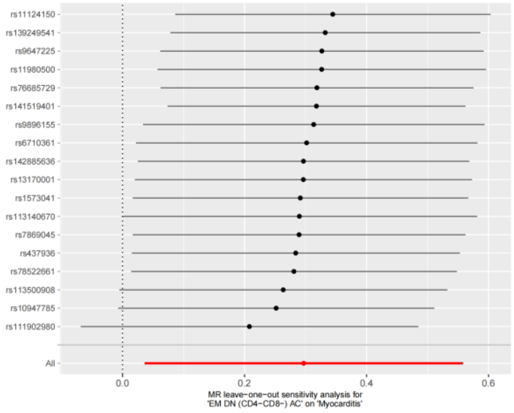
rs11124150
rs139249541
rs9647225
rs11980500
rs76685729
rs141519401
rs9896155
rs6710361
rs142885636
rs13170001
rs1573041
rs113140670
rs7869045
rs437936
rs78522661
rs113500908
rs10947785
rs111902980
All
0.0
0.2
0.4
0.6
MR leave−one−out sensitivity analysis for
'EM DN (CD4−CD8−) AC' on 'Myocarditis'

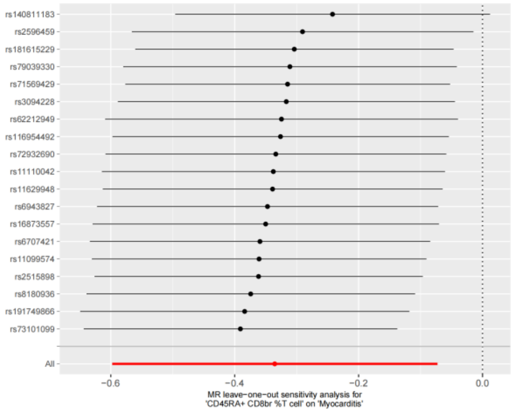
rs140811183
rs2596459
rs181615229
rs79039330
rs71569429
rs3094228
rs62212949
rs116954492
rs72932690
rs11110042
rs11629948
rs6943827
rs16873557
rs6707421
rs11099574
rs2515898
rs8180936
rs191749866
rs73101099
All
−0.6
−0.4
−0.2
0.0
MR leave−one−out sensitivity analysis for
'CD45RA+ CD8br %T cell' on 'Myocarditis'

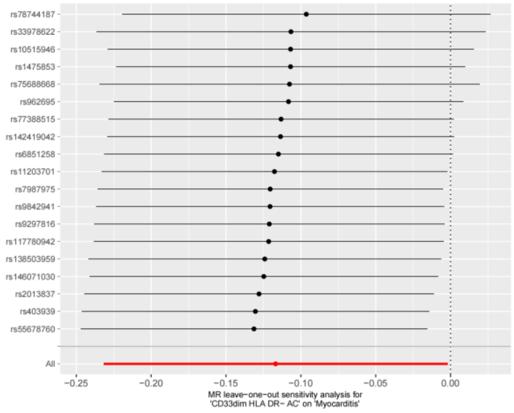
rs78744187
rs33978622
rs10515946
rs1475853
rs75688668
rs962695
rs77388515
rs142419042
rs6851258
rs11203701
rs7987975
rs9842941
rs9297816
rs117780942
rs138503959
rs146071030
rs2013837
rs403939
rs55678760
All
−0.25
−0.20
−0.15
−0.10
−0.05
0.00
MR leave−one−out sensitivity analysis for
'CD33dim HLA DR− AC' on 'Myocarditis'

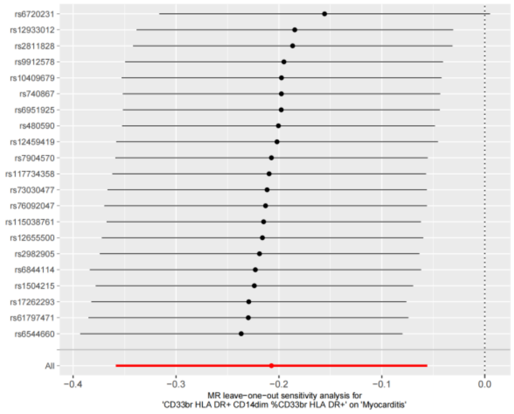
rs6720231
rs12933012
rs2811828
rs9912578
rs10409679
rs740867
rs6951925
rs480590
rs12459419
rs7904570
rs117734358
rs73030477
rs76092047
rs115038761
rs12655500
rs2982905
rs6844114
rs1504215
rs17262293
rs61797471
rs6544660
All
−0.4
−0.3
−0.2
−0.1
0.0
MR leave−one−out sensitivity analysis for
'CD33br HLA DR+ CD14dim %CD33br HLA DR+' on 'Myocarditis'

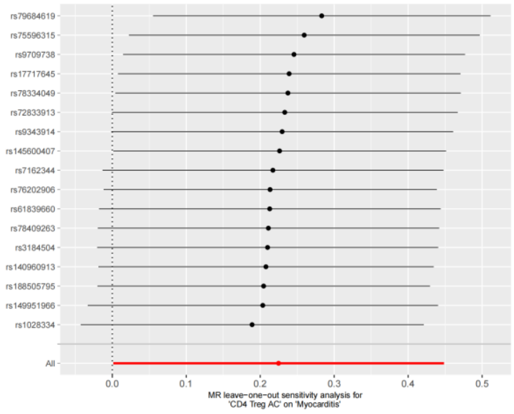
rs79684619
rs75596315
rs9709738
rs17717645
rs78334049
rs72833913
rs9343914
rs145600407
rs7162344
rs76202906
rs61839660
rs78409263
rs3184504
rs140960913
rs188505795
rs149951966
rs1028334
All
0.0
0.1
0.2
0.3
0.4
0.5
MR leave−one−out sensitivity analysis for
'CD4 Treg AC' on 'Myocarditis'

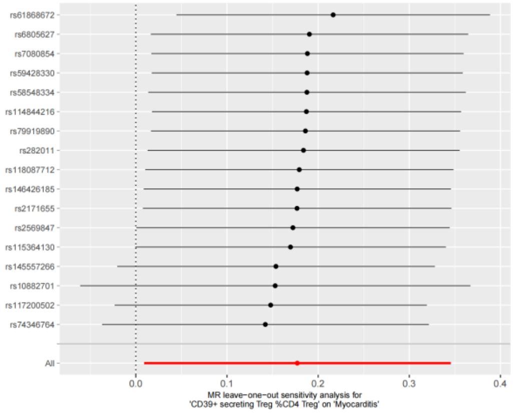
rs61868672
rs6805627
rs7080854
rs59428330
rs58548334
rs114844216
rs79919890
rs282011
rs118087712
rs146426185
rs2171655
rs2569847
rs115364130
rs145557266
rs10882701
rs117200502
rs74346764
All
0.0
0.1
0.2
0.3
0.4
MR leave−one−out sensitivity analysis for
'CD39+ secreting Treg %CD4 Treg' on 'Myocarditis'

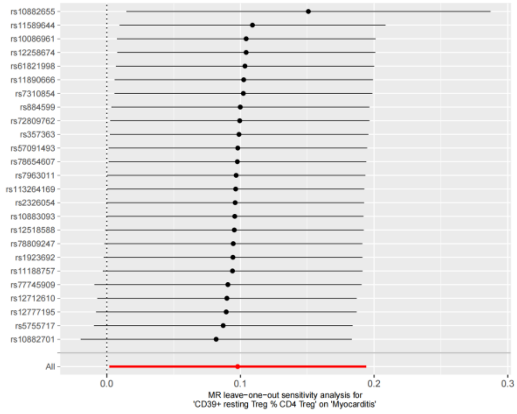
rs10882655
rs11589644
rs10086961
rs12258674
rs61821998
rs11890666
rs7310854
rs884599
rs72809762
rs357363
rs57091493
rs78654607
rs7963011
rs113264169
rs2326054
rs10883093
rs12518588
rs78809247
rs1923692
rs11188757
rs77745909
rs12712610
rs12777195
rs5755717
rs10882701
All
0.0
0.1
0.2
0.3
MR leave−one−out sensitivity analysis for
'CD39+ resting Treg % CD4 Treg' on 'Myocarditis'

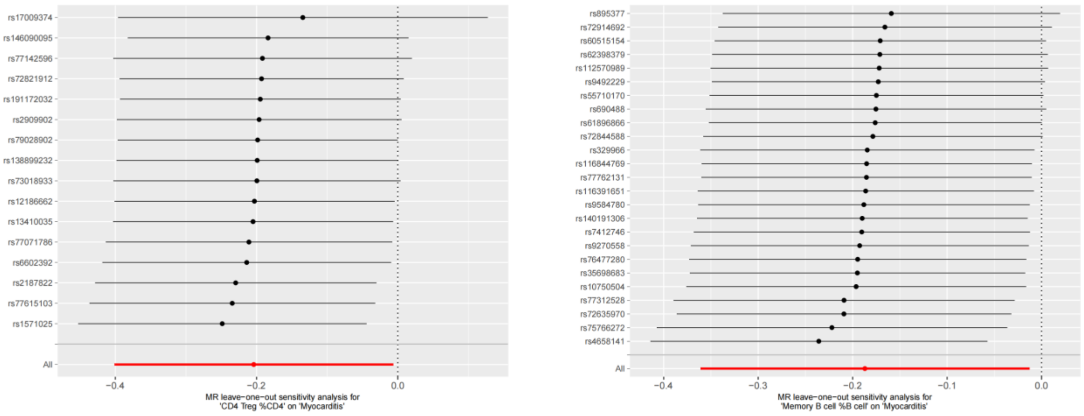

Figure S10. Leave-one-out sensitivity analysis for immune cell as exposures and myocarditis as outcomes.

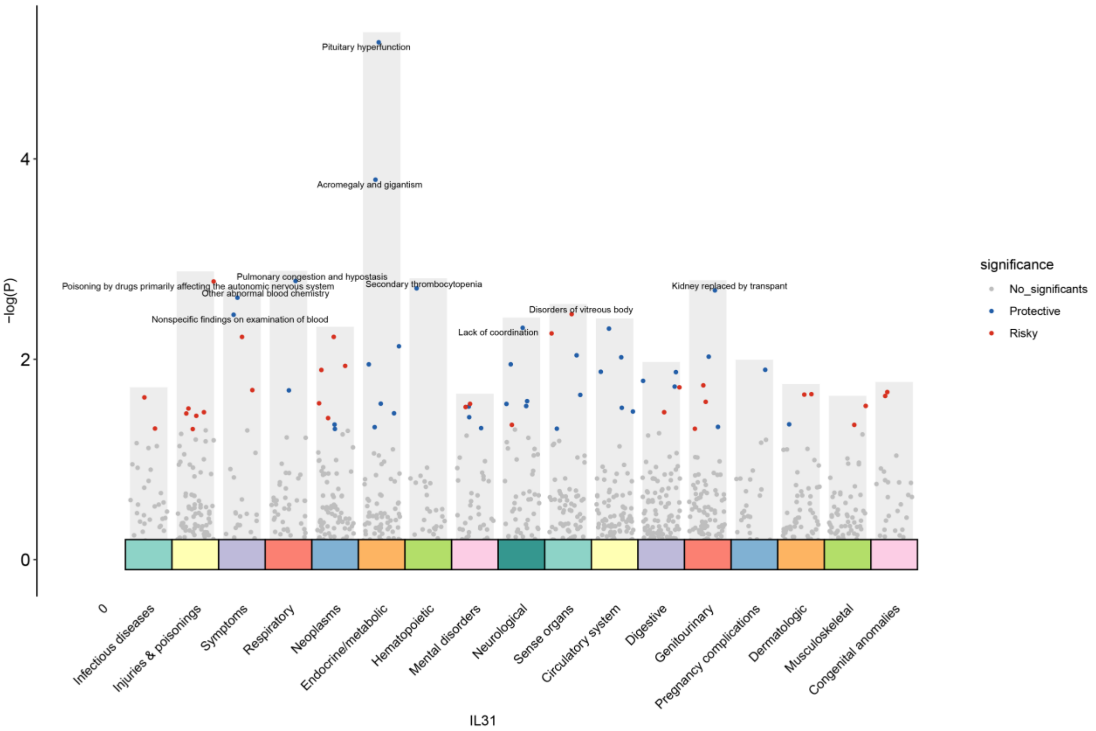

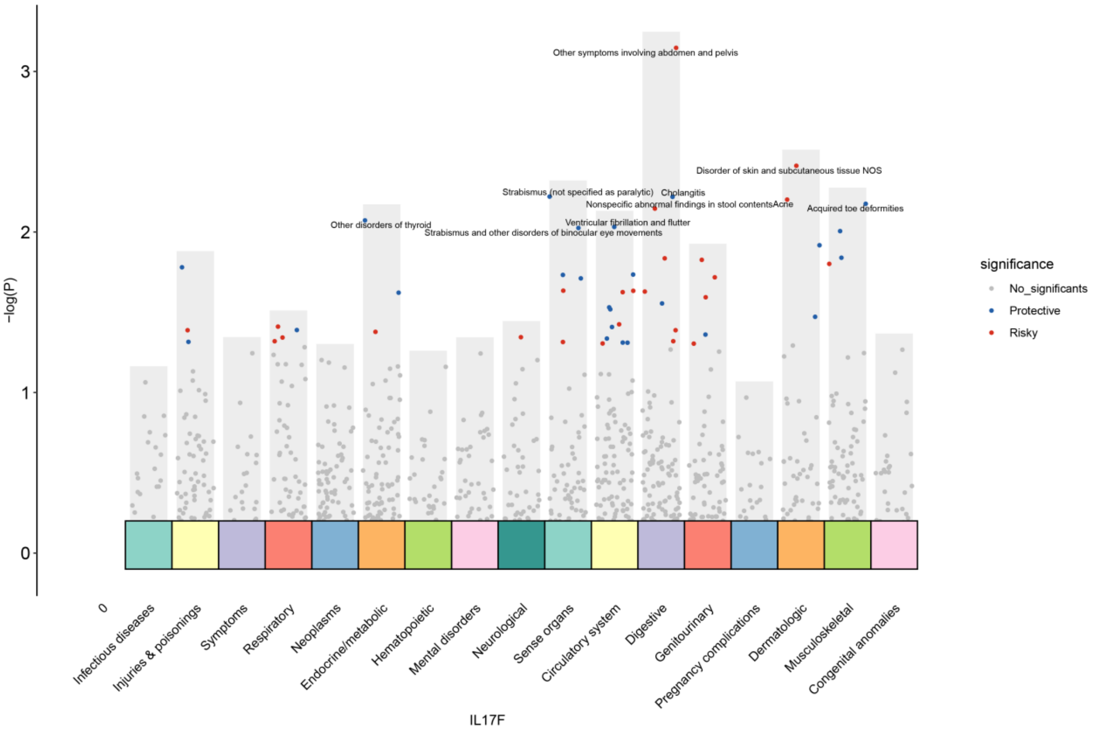

Other symptoms involving abdomen and pelvis
Disorder of skin and subcutaneous tissue NOS
Strabismus (not specified as paralytic)
Cholangitis
Nonspecific abnormal findings in stool contents
Acne
Acquired toe deformities
Other disorders of thyroid
Ventricular fibrillation and flutter
Strabismus and other disorders of binocular eye movements
significance
No_significants
Protective
Risky
-log(P)
0
1
2
3
Infectious diseases
Injuries & poisonings
Symptoms
Respiratory
Neoplasms
Endocrine/metabolic
Hematopoietic
Mental disorders
Neurological
Sense organs
Circulatory system
Digestive
Genitourinary
Pregnancy complications
Dermatologic
Musculoskeletal
Congenital anomalies
IL17F

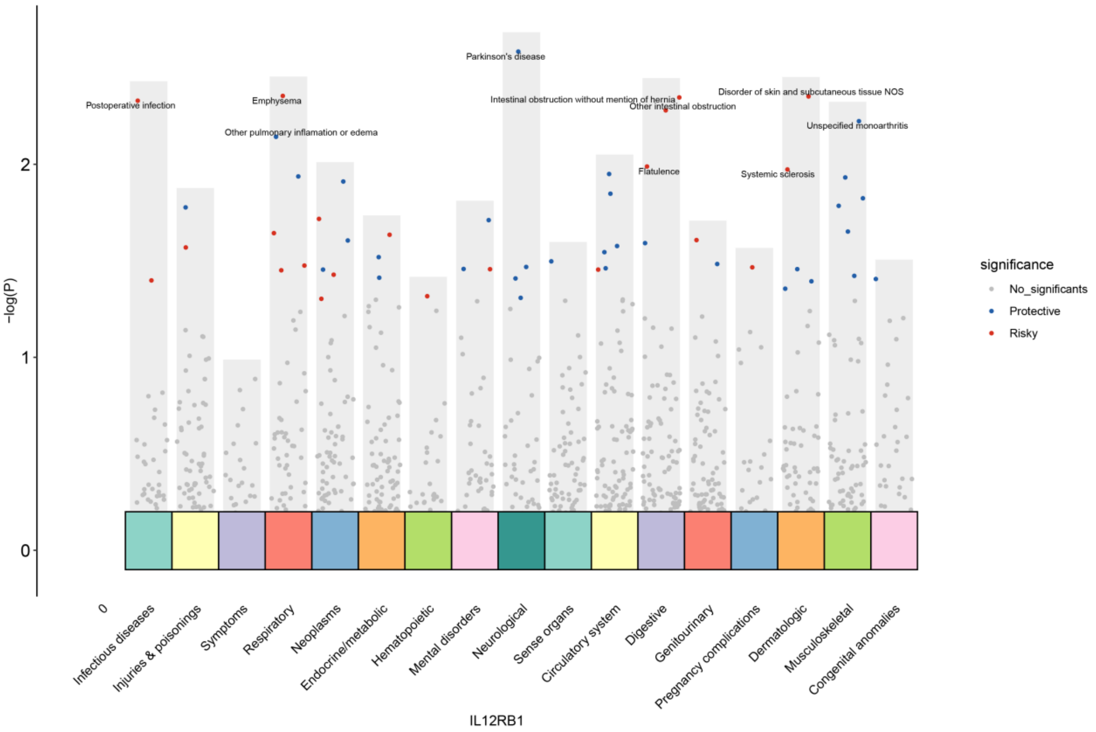

Parkinson's disease
Postoperative infection
Emphysema
Intestinal obstruction without mention of hernia
Other intestinal obstruction
Disorder of skin and subcutaneous tissue NOS
Unspecified monoarthritis
Other pulmonary inflammation or edema
Flatulence
Systemic sclerosis
significance
No_significants
Protective
Risky
-log(P)
0
1
2
Infectious diseases
Injuries & poisonings
Symptoms
Respiratory
Neoplasms
Endocrine/metabolic
Hematopoietic
Mental disorders
Neurological
Sense organs
Circulatory system
Digestive
Genitourinary
Pregnancy complications
Dermatologic
Musculoskeletal
Congenital anomalies
IL12RB1

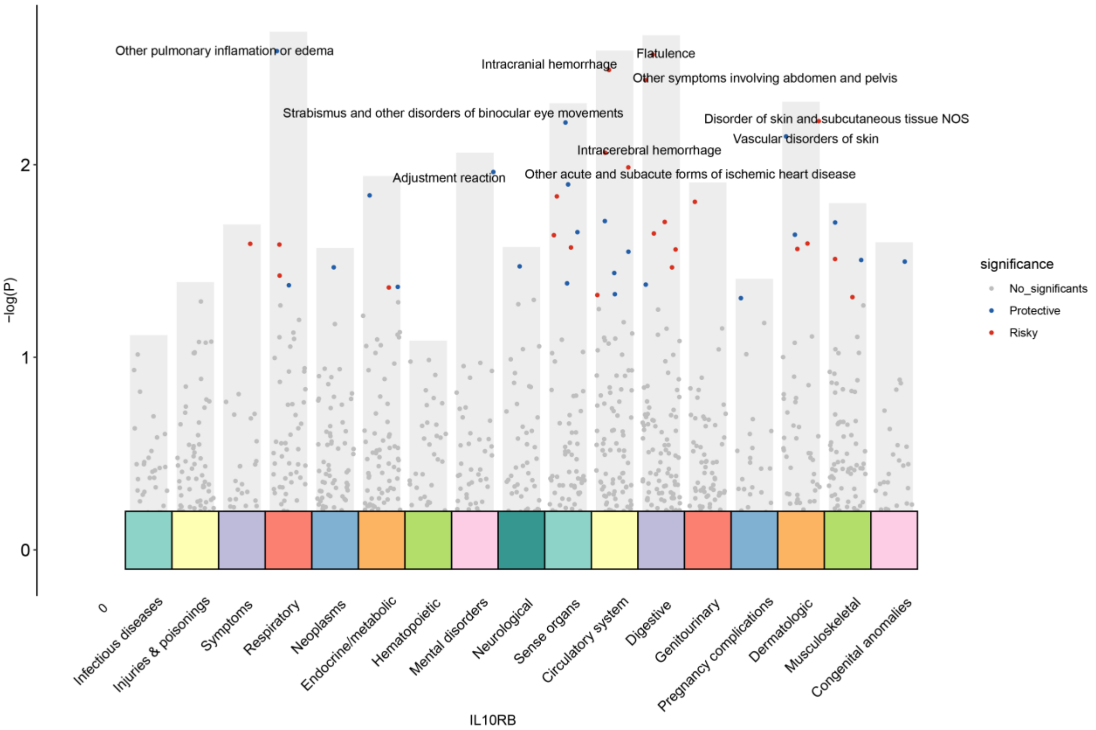

Other pulmonary inflamation or edema
Intracranial hemorrhage
Flatulence
Other symptoms involving abdomen and pelvis
Strabismus and other disorders of binocular eye movements
Disorder of skin and subcutaneous tissue NOS
Vascular disorders of skin
Intracerebral hemorrhage
Adjustment reaction
Other acute and subacute forms of ischemic heart disease
−log(P)
significance
No_significants
Protective
Risky
0
1
2
0
Infectious diseases
Injuries & poisonings
Symptoms
Respiratory
Neoplasms
Endocrine/metabolic
Hematopoietic
Mental disorders
Neurological
Sense organs
Circulatory system
Digestive
Genitourinary
Pregnancy complications
Dermatologic
Musculoskeletal
Congenital anomalies
IL10RB

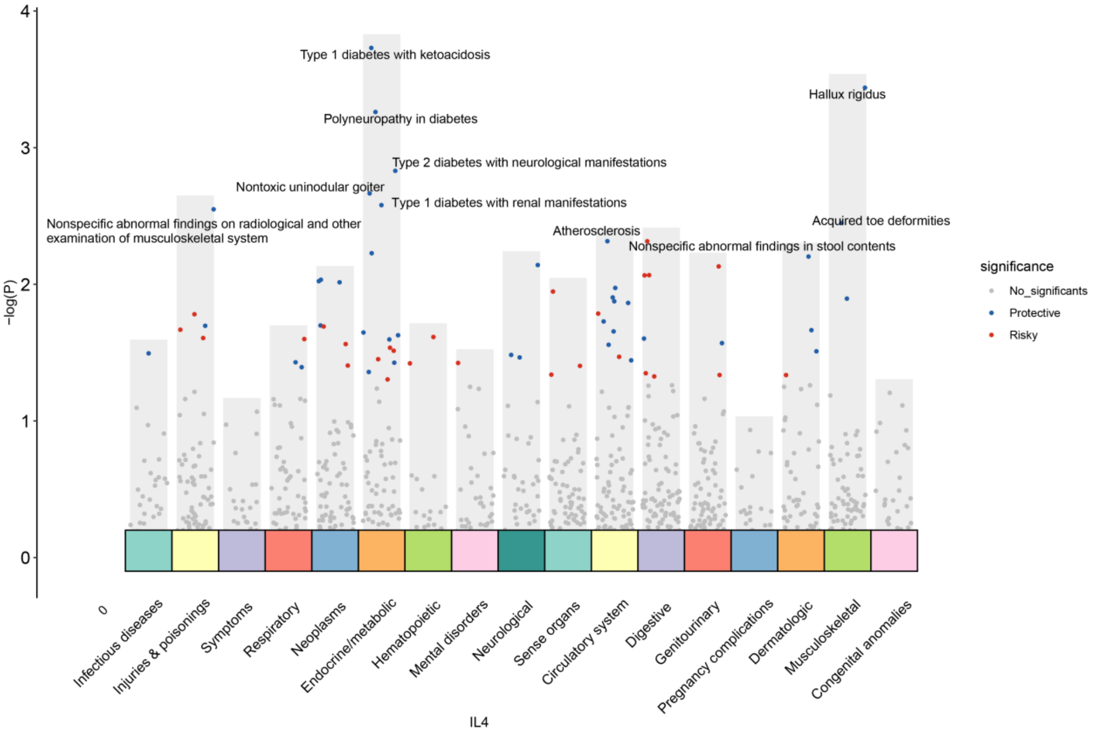

Type 1 diabetes with ketoacidosis
Hallux rigidus
Polyneuropathy in diabetes
Type 2 diabetes with neurological manifestations
Nontoxic uninodular goiter
Type 1 diabetes with renal manifestations
Nonspecific abnormal findings on radiological and other examination of musculoskeletal system
Acquired toe deformities
Atherosclerosis
Nonspecific abnormal findings in stool contents
−log(P)
significance
No_significants
Protective
Risky
0
1
2
3
4
0
Infectious diseases
Injuries & poisonings
Symptoms
Respiratory
Neoplasms
Endocrine/metabolic
Hematopoietic
Mental disorders
Neurological
Sense organs
Circulatory system
Digestive
Genitourinary
Pregnancy complications
Dermatologic
Musculoskeletal
Congenital anomalies
IL4

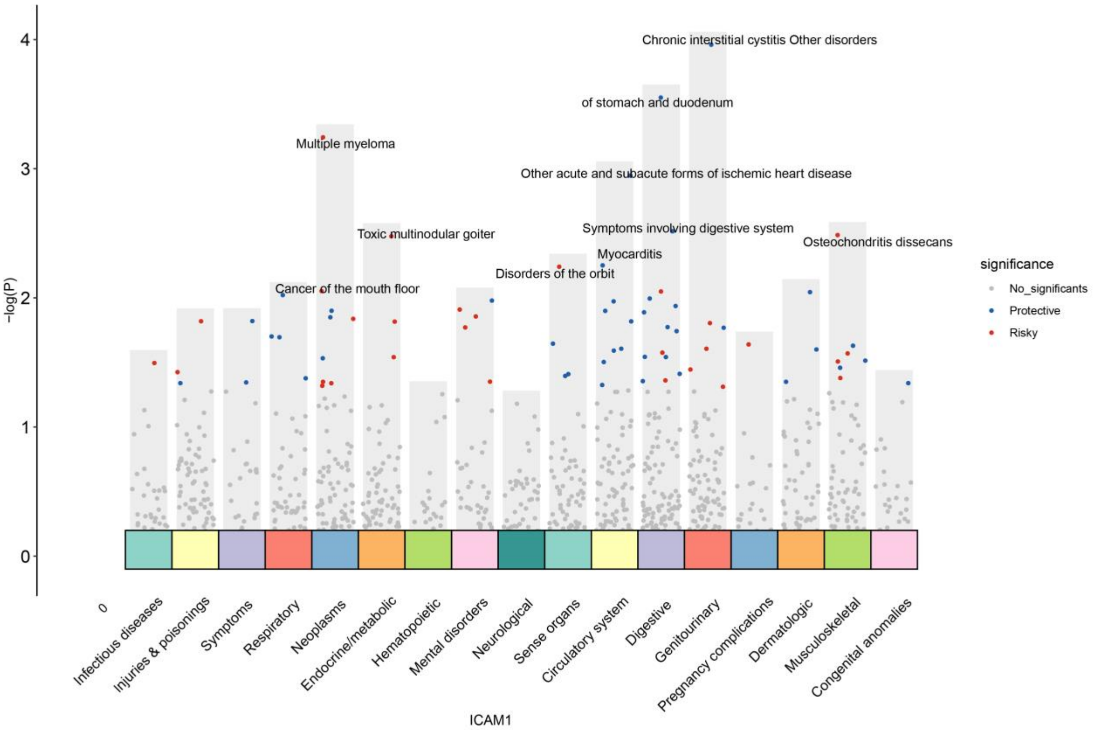

−log(P)
Chronic interstitial cystitis Other disorders
of stomach and duodenum
Multiple myeloma
Other acute and subacute forms of ischemic heart disease
Toxic multinodular goiter
Symptoms involving digestive system
Osteochondritis dissecans
Myocarditis
Disorders of the orbit
Cancer of the mouth floor
significance
No_significants
Protective
Risky
0
Infectious diseases
Injuries & poisonings
Symptoms
Respiratory
Neoplasms
Endocrine/metabolic
Hematopoietic
Mental disorders
Neurological
Sense organs
Circulatory system
Digestive
Genitourinary
Pregnancy complications
Dermatologic
Musculoskeletal
Congenital anomalies
ICAM1

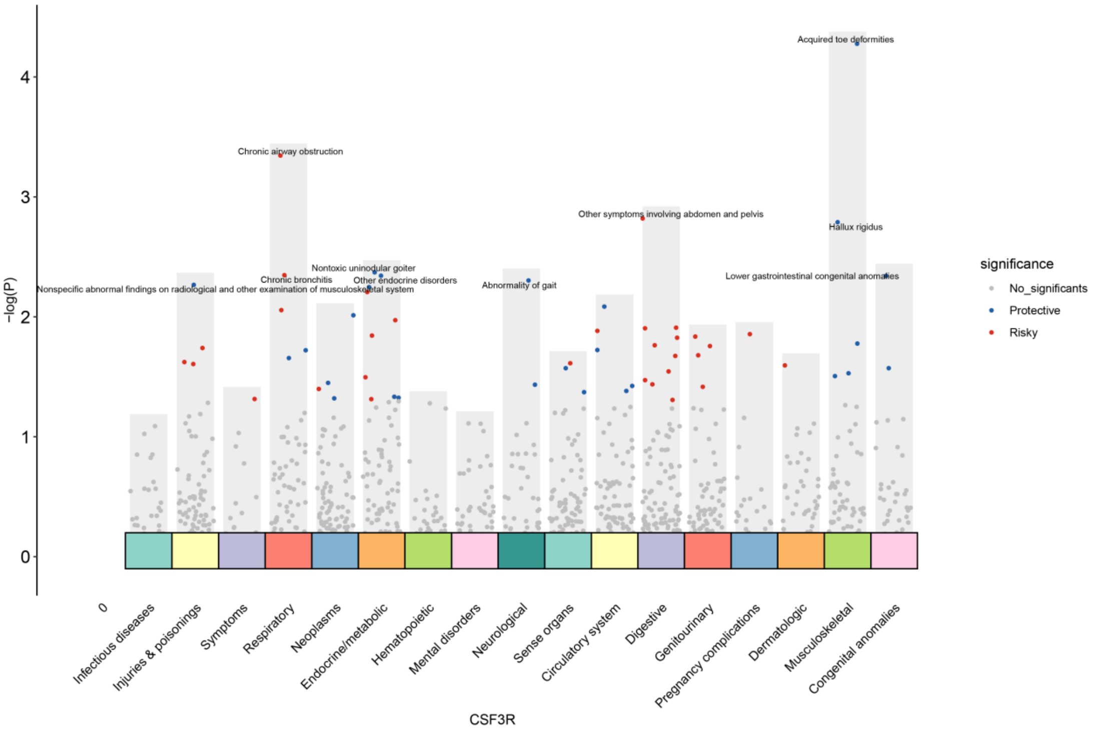

−log(P)
Acquired toe deformities
Chronic airway obstruction
Other symptoms involving abdomen and pelvis
Hallux rigidus
Nontoxic uninodular goiter
Chronic bronchitis
Other endocrine disorders
Nonspecific abnormal findings on radiological and other examination of musculoskeletal system
Abnormality of gait
Lower gastrointestinal congenital anomalies
significance
No_significants
Protective
Risky
0
Infectious diseases
Injuries & poisonings
Symptoms
Respiratory
Neoplasms
Endocrine/metabolic
Hematopoietic
Mental disorders
Neurological
Sense organs
Circulatory system
Digestive
Genitourinary
Pregnancy complications
Dermatologic
Musculoskeletal
Congenital anomalies
CSF3R

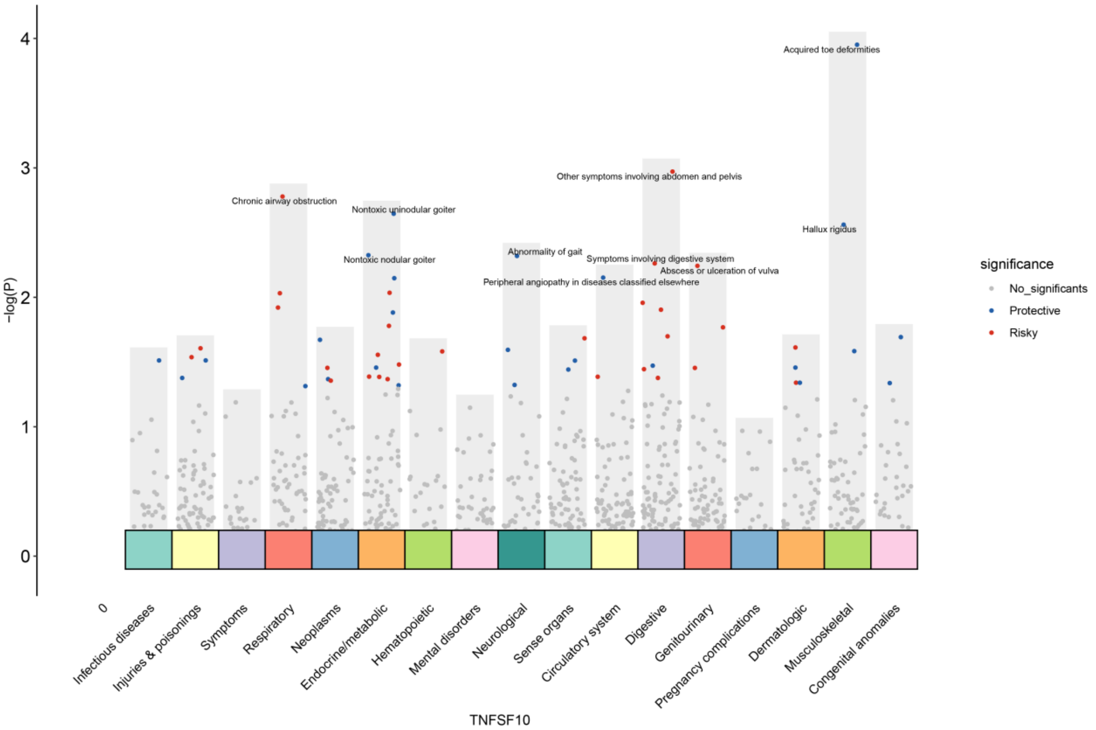

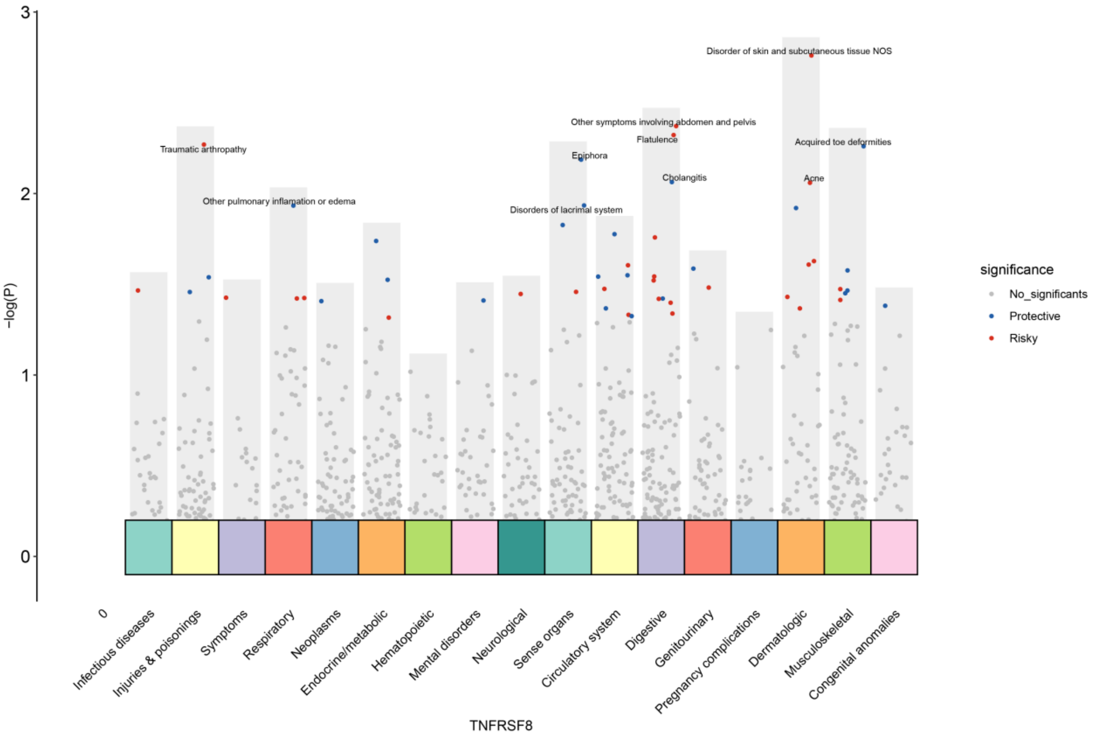

Figure S11. PheWAS analysis of proteins and other disease outcomes in UKB
